# Supplementary material for: KG-Microbe: Building modular and scalable knowledge graphs for microbiome and microbial sciences
Source: Gigascience. 2026 Jul 10;15:giag077. doi: 10.1093/gigascience/giag077 (PMC13536490; doi:10.1093/gigascience/giag077)
Supplement: Supplementary file 1 [file giag077_authors_response_to_reviewer_comments_original_submission.pdf]

Author's Response To Reviewer Comments

Manuscript Reviews:
—--------------------------------------
Reviewer #1: While the paper tackles a relevant issue and could serve as a valuable resource for the community, I have som
• I found the biological background insufficient for readers unfamiliar with key biological terms like "taxon" or "strain." Includ
publicly available sources. Microbial traits and functional attributes can be discovered with laboratory experiments or computa

We appreciate this important suggestion from the reviewer. We have adjusted the introduction to be more clear about introdu

• Additionally, the comparison between KG-Microbe and existing microbial KGs is not sufficiently clear. A comparative table h

This is a very helpful critique, and we have therefore introduced a new supplementary table (Supplementary Table 1) that co
chemicals, proteins, etc) as a comparison of the coverage of these sources. We refer to this supplementary table in Section 2

• The organization of the "Data Description" section somehow overlaps with the "Methods" section, leading sometimes to red
• Furthermore, some paragraphs in Section 2.1.2 would benefit from including concrete examples to enhance clarity. For exa
isolation source, are also modeled."

We thank the reviewer for these suggestions that help clarify and clean up the methods section. Throughout the Data Descrip
to the Methods Section 6.2.1, BacDive.

• I have several concerns about the evaluation.
• The approach to CQs is unclear. Typically, CQs are formulated in natural language, translated into formal graph queries (e.g
and detail the evaluation process.

This is a very helpful suggestion, and we thank the reviewers for this feedback. We have elaborated on the butyrate compete
taxa that exist in the gut microbiome. Three new competencies evaluated the (1) counts of HMP taxa found in the KG, (2) co
Questions, and new code is added to the kg-microbe-paper repository, where all queries for competencies are defined: https:

• The evaluation through ML also raises questions. Generating semantic representation using binary features over more recen

Thank you for providing this consideration. We have included more analyses of the choice of ML models used for evaluation a

• Additionally, while the SHAP value analysis is promising, Figure 7 is not easily interpretable — displaying enzyme labels inst

Thank you for these comments. The structure and labeling of Figure 7 have been reworked, resulting in an improvement in la
the Methods Section 6.5, Temperature Classification Model.

• Justifications for certain methodological choices are missing. For example, the exclusion of textual information (subsection

We thank the reviewer for this suggestion. We added a justification to our methodological choice of excluding free text from t
effectively categorized this pathway information. We now provide specific examples of this in the text (e.g. methane oxidatio

• The rationale for excluding negative information — critical in KGs operating under an open-world assumption — should be a

We added sections at the end of Section 2.1, KG-Microbe Content, and Section 4, Discussion explaining the open-world assum
make such assertions reliably. In keeping with standard practice for knowledge graphs developed under an open-world assum

• The scalability and reliability of manual mappings between taxonomies are concerning, given their inherent limitations. Mor

We appreciate the reviewer's feedback on this point. We added more of a discussion of this in Section 4, Discussion which ad

• While the methods are detailed, some subsections feel overly exhaustive. Summarizing parts of the methods and incorpora

We thank the reviewer for this suggestion. We attempted to remove unimportant information from Section 2, Data Descriptio

• Finally, as a broader consideration, it is unclear to me whether KG-Microbe should be considered a framework or solely a KG

We thank the reviewers for this feedback. We have updated Section 6.1, KG-Microbe Architecture to elaborate on the nature

Minor (TODOs)

• Figure 2 shows nodes like phRange and temperature without connections. Are these nodes meant to be isolated?

We have improved the structure of these figures and use gray bubbles to group entities which share predicates and data sour

• Pages 6, 10, and 26 have excessive blank spaces.

We ensured there are no excessive blank spaces.

• The hyperlink in reference 12 seems broken (I think the correct link is
https://nam02.safelinks.protection.outlook.com/?url=https%3A%2F%2Fzenodo.org%2Frecords%2F15026977&data=05%7C
LjAuMDAwMCIsIlAiOiJXaW4zMiIsIkFOIjoiTWFpbCIsIldUIjoyfQ%3D%3D%7C0%7C%7C%7C&sdata=gn6Gq6K%2FkTZB8hFUu
We updated this hyperlink for what is now reference 15.

• Figure 4 is split across pages.

We ensured that figure 4 is contained to one page.

• Formatting issues are present on page 33.
We believe we have resolved formatting issues.

• The title of Section 2.1.5 appears overly generic.

We updated this to be "Biomedically Relevant Insights: Drug, Disease, and Human Content"

• An overview of the methodology before Section 6.1 could help readers.

We included several sentences introducing section 6 to readers.

• There is repetition in Section 6.2.7: "Information about whether a given protein is reviewed or unreviewed is ingested direc

We removed the first reference to this.

• - On page 38, correct the following "Biological process, molecular function, and cellular component GO terms are extracted

We reviewed this text and could not find any inaccuracies, please expand on this if necessary.

• - On page 42, the sentence "Another semantic representation involving UniProt data was microbes capable of defined sets o

We updated this sentence to "Another semantic representation involving UniProt data showed which microbes were capable o

• I would reorganize the subsections in Section 6 to clarify that Sections 6.1 and 6.2 cover KG construction while Sections 6.3

This is a very helpful suggestion, and we have therefore organized the Methods subsections accordingly: 6.1 KG-Microbe Arch

—---------------------------------------

Reviewer #2:

- While the authors provide compelling competency questions, a comparative analysis with alternative microbial KG implemen

This is a very helpful critique, and we have therefore introduced a new supplementary table (Supplementary Table 1) that co
(microbes, chemicals, proteins, etc.) as a comparison of the coverage of these sources. We refer to this supplementary table

• Information on query response times, KG size impact on performance, or processing scalability when updating the KG (part

We appreciate the suggestion from the reviewer. We agree that query times can be a helpful metric for determining the scala
the kg-microbe github repository.

• The manuscript acknowledges potential inaccuracies in UniProt's predicted annotations, particularly butyrate production. Mo
Microbe predictions.

We appreciate this reviewer's suggestion. We added further discussion about the challenges of UniProt's functional annotation
knowledge in KG-Microbe to access the most accurate predictions. In Section 4 we clarify that in addition to the inclusion of c
of the assertion found.

Minor

• Figures are generally clear, but some visualizations (e.g., Figures 4 and 5) would benefit from better resolution or simplified
• - Although method details are robustly described, usability documentation such as example queries or a brief tutorial could

This is a very useful suggestion. We intend to continuously improve the user facing documentation (ReadMe and tutorials) an

• - Clearly highlighting more explicitly how KG-Microbe might evolve, particularly integration with other pathway-centric reso

Thank you for this feedback, we have elaborated on KG-Microbe applications, sustainability, and future direction in Section 4,
—---------------------------------------

Reviewer #3:

• In the first application, the authors assess the accuracy of KG-Microbe by comparing its annotations with those from Vital e
explicit.

We thank the review for this suggestion for further clarity. We added an explicit mention of the false negatives from these res
exclusive taxa as false positives, we consider these to be the more current set and focus on comparisons to BacDive results.

• Furthermore, the issue of redundant relations in UniProt is briefly mentioned, but requires a more in-depth discussion—part

We appreciate the reviewer's comment on this issue of redundant relations. We clarified the text in Section 4, Discussion to r
that collapsing equivalent nodes in the future may streamline some path analyses, however would not affect the results prese

• The third application is difficult to interpret. The authors should clearly describe the types of data extracted from the KG an
comprehension.

We thank the reviewer for this feedback and have worked to make this application clearer and more interpretable. The data u
EC nodes) and biochemical reactions (as Rhea nodes), we trained a gradient boosted decision tree classification model to pre
biolink:participates_in, enzymatic activity" and "protein, biolink:derives_from, microbe" exist, the corresponding "enzymatic a

• Although the authors reference other microbial knowledge graphs, a more systematic comparison is warranted. A comparat

This is a very helpful critique, and we have therefore introduced a new supplementary table (Supplementary Table 1) that co
knowledge (microbes, chemicals, proteins, etc) as a comparison of the coverage of these sources. We refer to this supplemen

4. To enhance usability, the software documentation should include several example queries to help users effectively explore

This is a very useful suggestion. We have updated user documentation to be more clear. We intend to continuously improve t

—---------------------------------------

Reviewer #4: Reproducibility
The study was reproducible because of access to the data uploaded in the Github repository or shared by email. To ensure th

• The authors should upload all required files to the GitHub repository.

We added instructions to download the ncbitaxon_nodes.tsv file to the ReadMe, and updated the notebook for Figure 7 in the

• All required packages required to run the script should be included, the package duckdb is missing from the readme file.

We added duckdb to the dependencies list in the ReadMe.

• The required Python package 'catboost' is currently not compatible with Python 3.13 or more recent versions. A note about

We added a note about this after the dependencies list in the ReadMe.

• The script requires a lot of memory to run. For computers with 24 GB ram or less the script is not able to execute. To impro

We thank the reviewer for this suggestion. We included the hardware requirements in the ReadMe.

• It would be helpful to include accompanying metadata files, for the datasets used or generated by the scripts, that explain:
• -The definition of each variable name.
• -The origin of each dataset (raw, processed, etc).
• -Any preprocessing steps applied before analysis.

Data sources are listed along with locations in download.yaml and stored in the data/raw subdirectory in the kg-microbe githu
custom transform code. Refer to Section 6.2, KG Construction and Modeling for detailed methods for how all data was proces
additional ontology trimming as in the case of the NCBI Taxonomy). The results of each transform are stored in the data/trar
subdirectory.

• register any new software application in the bio.tools and SciCrunch.org databases to receive RRID (Research Resource Ide
tracking, reproducibility and re-use of your tool.

We have registered the KG-Microbe framework and data resources at SciCrunch and Zenodo (10.5281/zenodo.14990848).

Editorial Manager®

Cookie Preference Center

We use cookies which are necessary to make our site work. We may also use additional cookies to analyze, improve and personalize our content and your digital experience. For more information, see our Cookie Policy.

You may choose not to allow some types of cookies. However, blocking some types may impact your experience of our site and the services we are able to offer. See the different category headings below to find out more or change your settings.

Allow all

Manage Consent Preferences

Strictly Necessary Cookies

Always active

These cookies are necessary for the website to function and cannot be switched off in our systems. They are usually only set in response to actions made by you which amount to a request for services, such as setting your privacy preferences, logging in or filling in forms. You can set your browser to block or alert you about these cookies, but some parts of the site will not then work.

Functional Cookies

These cookies enable the website to provide enhanced functionality and personalisation. They may be set by us or by third party providers whose services we have added to our pages. If you do not allow these cookies then some or all of these services may not function properly.

Performance Cookies

These cookies allow us to count visits and traffic sources so we can measure and improve the performance of our site. They help us to know which pages are the most and least popular and see how visitors move around the site.

Targeting Cookies

These cookies may be set through our site by our advertising partners. They may be used by those companies to build a profile of your interests and show you relevant adverts on other sites. If you do not allow these cookies, you will experience less targeted advertising.

Cookie List

Clear

Apply Cancel

Consent Leg.Interest

Confirm my choices

Power
