# Supplementary material for: KG-Microbe: Building modular and scalable knowledge graphs for microbiome and microbial sciences
Source: Gigascience. 2026 Jul 10;15:giag077. doi: 10.1093/gigascience/giag077 (PMC13536490; doi:10.1093/gigascience/giag077)
Supplement: Supplementary file 2 [file giag077_authors_response_to_reviewer_comments_revision_1.pdf]

Author's Response To Reviewer Comments

Manuscript Reviews:
—---------------------------------------
Reviewer #1: I thank the authors for their revisions and responses to my previous comments. The revised manuscript shows improvement in clarity and structure. However, the paper is quite long in its current form, which may affect readability. The authors might want to consider moving some of the more detailed content to the supplementary materials.

We appreciate this suggestion from the reviewer. We have moved this detailed content into the Supplementary Methods section 1.1-1.11.

Minor comments:
- Correct the reference in the sentence: "SHAP values for all features can be found in XXXX."

Thank you for catching this, we have revised the sentence.

- Regarding the sentence "Biological process, molecular function, and cellular component GO terms are extracted from ...", my earlier comment concerned sentence structure (not content). I suggest revising it to something like "GO terms for biological process, molecular function, and cellular component are extracted from ..."

We appreciate the stylistic recommendations, we have updated this sentence to: "GO terms for biological process, molecular function, and cellular component are extracted from the "go" field, excluding any text or brackets, and linked to microbes via the predicate "biolink:participates_in", "biolink:participates_in", or "biolink:located_in", respectively."

Reviewer #4:

-- Uploading files in the Github repository. The authors have added the necessary files to the GitHub or instructions for how to download them. However, However, several intermediate files required to reproduce the full workflow remain unavailable. For example, the intermediate file "Gold_Standard_Species_Overlap_butyrate_produces.csv" could not be reproduced due to computational limitations and was shared by the authors via email instead of being made publicly available.To allow independent reproduction of the analyses, including those in the kg-microbe-paper repository, we recommend that all intermediate files be shared in the public repository or that clear instructions be provided to generate them.
-- Updating the Readme file with environment information. The authors have updated the readme file with the required Python packages. But they did not add information on hardware requirements (for computers with 24 GB ram or less the script is not able to execute). And we encourage the authors to include the hardware requirements in the README file.

We appreciate the reviewer ensuring reproducibility here. We uploaded the mentioned intermediate file (Gold_Standard_Species_Overlap_butyrate_produces.csv) and added a statement in the ReadMe of how to access this file if the script is unable to run to completion in Step 6 of the ReadMe. In addition we improved the organization of the primary input and intermediate files, now all located in specific subdirs in data/. A few optimizations were added to the make file and python scripts and verified to produce the exact results. Here is the summary:

1. Package Management Migration

- Poetry → uv: ~10x faster dependency resolution and installation

- Reduced environment setup time from minutes to seconds

2. Memory Optimization

- Created Classification_gold_standard_comparison_optimized.py
- Fixes OOM errors on systems with <200 GB RAM
- Uses DuckDB in-memory queries instead of loading entire 62 GB dataset into pandas

3. Files Moved to data/Input_Files/

All primary input files are now organized as follows:

```
data/Input_Files/
├── Gold_Standard_Species_Overlap_butyrate_produces.csv (373 KB - tracked in git)
├── KGMicrobe-biomedical-function-20250222.tar.gz (4.3 GB - git-ignored)
├── kg-microbe-biomedical-function-cat/ (directory - git-ignored)
│   ├── merged-kg_edges.tsv (29 GB)
│   ├── merged-kg_edges_noEC.tsv (9.5 GB)
│   ├── merged-kg_edges_competency_specific_ec.tsv (9.2 GB)
│   ├── merged-kg_edges_ncbitaxon.tsv (45 MB)
│   └── merged-kg_nodes.tsv (15 GB)
├── ncbitaxon_nodes.tsv (130 MB - git-ignored)
└── equilibrator_cache/ (directory - git-ignored)
├── compounds.sqlite (130 MB - downloaded on first run)
└── cc_params.npz (50 MB - downloaded on first run)
```

4. Python Scripts Updated (12 files)

All references updated from ./data/ to ./data/Input_Files/ and from ./src/ to ./data/:

- src/Classification_gold_standard_comparison.py
- src/Classification_gold_standard_comparison_optimized.py
- src/Competencies.py (also added appdirs monkey-patch for equilibrator cache configuration)
- src/Functional_Annotations.py
- src/Functional_Annotations_EC.py
- src/Gold_standard_Competency_analysis.py
- src/Process_competency_questions.py
- src/classification.py
- src/classification_permutations.py
- src/classification_utils.py (fixed makedirs path)
- src/gut_microbes_competencies.py
- src/ncbi_phylogeny_search.py

5. .gitignore Updated

Added multiple exclusions:
- *.tar.gz - Exclude all tar archives
- data/Input_Files/* - Exclude all files in Input_Files
- data/Input_Files/*/ - Exclude all subdirectories in Input_Files
- !data/Input_Files/Gold_Standard_Species_Overlap_butyrate_produces.csv - Exception to track Gold Standard file
- data/Input_Files/equilibrator_cache/ - Exclude equilibrator cache

6. Makefile Updated

- Updated clean target to remove data/Input_Files/equilibrator_cache

7. Equilibrator Cache Configuration

src/Competencies.py now includes:
- Patch of appdirs.user_cache_dir() to redirect equilibrator cache to data/Input_Files/equilibrator_cache/
- This ensures both compounds.sqlite and cc_params.npz download to the project directory

8. Documentation Updated

- README.md: Updated path references:
- './Phylogeny_Search' → './data/Phylogeny_Search'
- './src/Intermediate_Files' → './data/Intermediate_Files'
- Added note about equilibrator cache auto-download

9. Final Directory Structure

```
data/
├── Input_Files/ # All downloaded/provided input files (mostly git-ignored)
│  ├── Gold_Standard_*.csv # Tracked in git
│  ├── *.tar.gz, *.tsv # Git-ignored downloads
│  ├── kg-microbe-biomedical-function-cat/ # Git-ignored KG files
│  └── equilibrator_cache/ # Git-ignored equilibrator cache
├── Intermediate_Files/ # Processing results (tracked in git)
│  ├── HMP_Microbes_Mapped.csv
│  ├── HMP_Microbes_Mapped_Summary.csv
│  ├── IBD_Classification_butyrate_producers_summary.csv
│  └── PD_Classification_butyrate_producers_summary.csv
├── Intermediate_Files_Competencies/ # Competency analysis (tracked in git)
├── hmp_supplementary/ # HMP supplementary Excel files (tracked in git)
├── ML_model_shap/ # SHAP feature importance (tracked in git)
└── Phylogeny_Search/ # Cached taxonomy (tracked in git)
```

10. Key Improvements

a. Complete path consolidation: All data files now under data/, no references to src/ data directories
b. Equilibrator cache control: Downloads to project directory instead of home directory (platform-agnostic)
c. Documentation path updates: README.md and CLAUDE.md fully synchronized
d. Proper git tracking: Small reference files tracked, large downloads excluded
e. Generated outputs committed: 156 analysis output files (~80 MB) now tracked in git

-- Add metadata to the dataset. The information provided by the authors mainly describes the data processing workflow and file structure, not the metadata describing the datasets. For better transparency and FAIR compliance, we recommend that the authors include explicit metadata documentation (for example, a data dictionary or metadata table describing each dataset, variable names, formats, and data provenance).

We appreciate the suggestion. Section 2, Data Description, gives a high level explanation of each resource and its content. In Section 6.2 is Table 1, which describes the type of data included for each dataset. These descriptions ensure that we've explained the purpose of each resource and how it fits into the broader KG resource. Finally, we have moved the detailed documentation of how we used each dataset, variable names, and formats to Supplementary Methods 1.1-1.11 to ensure that all metadata included from each dataset is clear.

- Summary of the second computational reproducibility review
The results of the original study were reproduced during the first review with a minor numerical error. The result was consistent for inflammatory bowel disease, but differed

slightly for Parkinson's disease (PD):
- The reproduced p-value for PD was 8e-293 as opposed to the reported p-value of 2e-288

- The reproduced value of the test statistic for PD was 1337 whereas the reported value was 1317.

These numerical discrepancies are minor. We verified the chi-squared results with the reported contingency table (figure 6C) in R and obtained the same chi-squared statistic and p-value as reported in the manuscript, which suggests that the difference observed in the first review likely resulted from an issue when running the original code to generate the contingency table. However, as intermediate files are not shared in the repository, we were unable to re-run the complete analysis to confirm the exact source of the discrepancy. We therefore recommend that the authors double-check this part of the analysis to ensure full computational reproducibility.

We greatly appreciate the reviewer's thorough investigations of the reproducibility of this analysis. We recognize slight numerical differences may arise due to data and software dependencies changing over time, though we ensured that the results themselves as well as the scientific interpretation of these results have not changed given these p-value and chi-square statistics. We determined that these changes are due to updates to the NCBITaxon owl file through the OwlReady package, which is accessed in the 4th script of the pipeline (Classification_gold_standard_comparison.py), and updates to the Equilibrator API in the second script of the pipeline (Process_competency_questions.py). In addition extreme p-value calculations may have platform and package version-specific sensitivity. In the latest reproduction, one less extreme p-value matched exactly; the other (more extreme) p-value shifted by 5 in the base-10 exponent. We made additional code edits to further optimize the competencies and also cache the Equilibriator results.

We appreciate this reviewers' detailed analysis of the change of these results. After reviewing the results reported in the manuscript and our own analysis, we confirm that these are correctly stated in the manuscript.

Author Decision Letter

In addition, please register any new software application in the bio.tools and SciCrunch.org databases to receive RRID (Research Resource Identification Initiative ID) and biotoolsID identifiers, and include these in your manuscript. Computational workflows should be registered in workflowhub.eu and the DOIs cited in the relevant places in the manuscript. These will facilitate tracking, reproducibility and re-use of your tool.

The KG-Microbe framework has been registered at these resources:
1. https://rrid.site/data/record/nlx_144509-1/SCR_026589/resolver
2. https://bio.tools/kg-microbe
3. https://workflowhub.eu/workflows/2044
4. https://doi.org/10.48546/WORKFLOWHUB.WORKFLOW.2044.1

All web links and URLs should be given a reference number and included in the reference list rather than within the text of the manuscript. Please remove the URLs, cite them as reference and adjust the order of the reference accordingly. Except for “Abstract” and "Availability of Source Code and Requirements"

We thank the editor for noticing this. We have updated all citations and removed any URLs from the main manuscript in the relevant sections.

Cookie Preference Center

We use cookies which are necessary to make our site work. We may also use additional cookies to analyze, improve and personalize our content and your digital experience. For more information, see our Cookie Policy.

You may choose not to allow some types of cookies. However, blocking some types may impact your experience of our site and the services we are able to offer. See the different category headings below to find out more or change your settings.

Allow all

Manage Consent Preferences

Strictly Necessary Cookies

Always active

These cookies are necessary for the website to function and cannot be switched off in our systems. They are usually only set in response to actions made by you which amount to a request for services, such as setting your privacy preferences, logging in or filling in forms. You can set your browser to block or alert you about these cookies, but some parts of the site will not then work.

Functional Cookies

These cookies enable the website to provide enhanced functionality and personalisation. They may be set by us or by third party providers whose services we have added to our pages. If you do not allow these cookies then some or all of these services may not function properly.

Performance Cookies

These cookies allow us to count visits and traffic sources so we can measure and improve the performance of our site. They help us to know which pages are the most and least popular and see how visitors move around the site.

Targeting Cookies

These cookies may be set through our site by our advertising partners. They may be used by those companies to build a profile of your interests and show you relevant adverts on other sites. If you do not allow these cookies, you will experience less targeted advertising.

Cookie List

Clear

Apply Cancel

Consent Leg.Interest

Confirm my choices

Powe
