# Supplementary material for: KG-Microbe: Building modular and scalable knowledge graphs for microbiome and microbial sciences
Source: Gigascience. 2026 Jul 10;15:giag077. doi: 10.1093/gigascience/giag077 (PMC13536490; doi:10.1093/gigascience/giag077)
Supplement: Supplementary file 3 [file giag077_authors_response_to_reviewer_comments_revision_2.pdf]

Author's Response To Reviewer Comments

Response to Reviewer #4
We thank Reviewer #4 for a third careful round of computational reproducibility assessment, and in particular for the detailed step-by-step reproduction protocol in Section 5.2. Acting on that protocol, we identified and fixed a stale-state contamination bug in the disease-classification strain-enumeration step, resolving the run-to-run instability observed across review rounds, as described in Section 1 below. Separately, in re-examining the contingency table flagged by the reviewer, we identified a way to improve the applicability and confidence of the statistical test by deduplicating the strain counts underlying Figure 6C. We describe and adopt this revision in Section 2 below. The fixes and validation work described below are a direct result of the reviewer's protocol for which we are very grateful.
1. Overall assessment and what changed since round 2
Reviewer's comments:
"Summary of the third computational reproducibility review
The main computational workflow could be executed and the expected output files for the analysis were generated.
The results were not fully reproducible even if they confirmed a highly statistically significant association for both inflammatory bowel disease and Parkinson's disease. The reproduced numerical results were not identical to those reported in the manuscript. For both conditions, the counts of "decreased" and "increased" values in the contingency table reproduced differ from the values in figure 6C, which may explain the observed differences in the test results.
For inflammatory bowel disease, the reproduced chi-squared statistic was 672 with p-value 4e-148, compared with the reported chi-squared statistic of 647 and p-value of 1e-142. For Parkinson's disease, the reproduced chi-squared statistic was 1064 with p-value 3e-233, compared with the reported chi-squared statistic of 1317 and p-value of 2e-288.
The reproduced numerical results in the present review also differed from those obtained in the previous round reviews, where the reproduced values obtained were a chi-squared statistic of 647 with p-value 1e-142 for IBD and chi-squared statistic of 1338 with p-value 8e-293 for PD.
This indicates that, although the qualitative conclusion of a highly significant association is robust, the exact numerical results are not fully stable across computational reproductions.
- Follow-up on previous recommendations:
The authors addressed several of the previous comments appropriately. In particular, the previously missing intermediate file is now publicly available in the repository, the file structure and documentation were improved, and a memory-optimized classification script was added. These changes substantially improved reproducibility compared with the previous round.
We recommend that the authors rerun the analysis for Figure 6C, verify the dataset and intermediate outputs underlying the reported table values, and revise the manuscript table if needed so that the published values match the reproducible code output."
To resolve the discrepancies, we re-ran the entire Figure 6C analysis end-to-end with three reproducibility-critical changes from the originally published pipeline: (1) Corrected code — we fixed the stale-state strain-enumeration bug described below (src/ncbi_phylogeny_search.py). (2) Frozen NCBI Taxonomy — the taxonomic rank table is now shipped in-repo (data/Phylogeny_Search/ncbitaxon_rank.tsv.gz) instead of being re-fetched from the live NCBITaxon OWL, eliminating ontology-version drift between runs. (3) Cached eQuilibrator results — the thermodynamic reaction-direction predictions are now persisted in data/Input_Files/equilibrator_cache/ instead of being recomputed against the live eQuilibrator API, eliminating remote-service drift. The input knowledge graph (KG-Microbe-Biomedical-Function, release 2025-03-07), the disease-association sources (Disbiome and Wallen et al.), the original per-parent contingency-table construction used for this reproducibility self-check, and the chi-square test itself were unchanged. All intermediate strain/species JSONs were regenerated from scratch in an empty output directory (i.e., no cached state inherited from prior runs). The resulting chi-square values are shown in the "Revised values" row of the table below, alongside the originally published values and Reviewer #4's reproductions from rounds 2 and 3:
Source
IBD χ^2
IBD p-value

PD χ^2
PD p-value
Manuscript Figure 6C (original)
647
1×10^{-142}
1317
2×10^{-288}
Reviewer #4, round 2
647
1×10^{-142}
1338
8×10^{-293}
Reviewer #4, round 3
672
4×10^{-148}
1064
3×10^{-233}
Reproduced per-parent (same analysis as original; deterministic re-run)
674.63
9.8×10^{-149}
1337.36
8.6×10^{-293}
Figure 6C revised, deduplicated (current figure)
497.26
3.7×10^{-110}
918.80
8.0×10^{-202}

Thanks to Reviewer #4’s detailed reproduction protocol, we identified a stale-state contamination bug in the disease-classification pipeline’s strain-enumeration step. When the analysis started, it loaded every strain and species data file already present in the shared output directory — including outputs from unrelated butyrate competency analyses — into its in-memory working state before running its own enumeration. The disease-specific enumeration then appended its results on top of that polluted starting state, producing duplicate strain entries and off-target taxa in the IBD and PD contingency tables. In our pipeline this contamination shifted the chi-square statistic by only a few percent relative to the originally published values (647 → 674.63 for IBD; 1317 → 1337.36 for PD), but it was the dominant source of the run-to-run instability that Reviewer #4 observed across rounds, because the contaminating state varied depending on which prior runs had populated the output directory. We fixed this by starting the disease-classification step from a fresh state, so that only the disease-specific enumeration populates the working state, making each run deterministic.

Using the corrected code, the per-parent contingency now reproduces deterministically and matches the originally published Figure 6C to within external-dependency drift: $\chi^2 = 674.63$, $p = 9.8 \times 10^{-149}$, for IBD and $\chi^2 = 1337.36$, $p = 8.6 \times 10^{-293}$, for PD. This confirms that the reproducibility issues raised by the reviewer have been resolved.

The small residual numerical shift from the originally published chi-square values (647 → 674.63 for IBD; 1317 → 1337.36 for PD; ~1–4% per disease) reflects ontology-version drift in the NCBI Taxonomy OWL between publication and now, plus a small contribution from eQuilibrator’s reaction-direction predictions when re-queried against the live API. Both are now frozen in-repo (data/Phylogeny_Search/ncbitaxon_rank.tsv.gz and data/Input_Files/equilibrator_cache/), so subsequent reproductions are deterministic. These small numerical differences have no effect on the conclusions of the manuscript.

2. Strengthening the statistical test: a deduplicated contingency for Figure 6C

Reviewer #4's request that we verify the dataset and intermediate outputs underlying the reported table values prompted us to scrutinize the contingency construction itself. In doing so, we recognized that the original per-parent construction counted strain descendants non-independently. Descendants were

enumerated separately beneath each disease-associated taxon, so a single strain could be counted once for every annotated ancestor from which it descends. In addition, because the conflicting-direction filter operated on the source taxa rather than their descendants, a strain could be counted in both the increased- and decreased-likelihood bins through different parent taxa. Quantitatively, the per-parent IBD contingency contained 54,879 descendant occurrences spanning only 24,948 unique strains, of which 9,580 appeared in both bins; the PD contingency contained 25,370 occurrences spanning 14,487 unique strains, of which 1,029 appeared in both bins. This double-counting inflated the effective sample size seen by the chi-square test. We applied a strict removal of cross-direction conflicts because their disease direction is ambiguous in the source data. Each strain is counted once, and strains with conflicting disease-direction assignments are removed. Figure 6C has been revised to report this deduplicated contingency. Under the deduplicated analysis, butyrate-producer enrichment among decreased-likelihood taxa remains highly significant: IBD $\chi^2 = 497.26$, $p = 3.7 \times 10^{-110}$, and PD $\chi^2 = 918.80$, $p = 8.0 \times 10^{-202}$, as shown in the updated Figure 6C. Because each strain now contributes a single observation, the cross-parent multiplicity that motivated this revision is removed. Residual within-parent phylogenetic clustering does not affect the qualitative conclusion: complete sensitivity analyses, including a cluster-aware permutation test that respects the parent-to-strain hierarchy, are reported in the per-disease sensitivity CSVs in the public repository: data/Intermediate_Files/figure6c_sensitivity_{IBD,PD}.csv.

Response to the Editor

Thank you for coordinating the third round of review and for the constructive feedback. We are pleased that Reviewer #1 finds the paper ready for publication, and we are grateful to Reviewer #4 for their continued, careful computational reproducibility assessment. The detailed step-by-step reproduction protocol in Section 5.2 of their report was particularly useful: it allowed us to identify the reproducibility-critical components of our pipeline (a stale-state contamination bug in the strain-enumeration step, live-API drift in eQuilibrator, and ontology-version drift in NCBI Taxonomy) and to freeze each of them so that future reproductions result in consistent values.

In addition, while resolving these issues, we identified an opportunity to strengthen the statistical test underlying Figure 6C. The original contingency counted each strain once per disease-associated ancestor, so a strain could be counted multiple times and, in some cases, in both disease directions even after removing strain-level conflicts in the Disbiome data. We have revised Figure 6C to use a deduplicated contingency table, in which each strain is counted once and all strains with conflicting disease-direction assignments are removed. This removes cross-parent repeated strain counts from the Figure 6C contingency ßwhile leaving the qualitative conclusion unchanged

All software is registered in all recommended resources (data and code).

Editorial Manager®

Cookie Preference Center

We use cookies which are necessary to make our site work. We may also use additional cookies to analyze, improve and personalize our content and your digital experience. For

more information, see our Cookie Policy.

You may choose not to allow some types of cookies. However, blocking some types may impact your experience of our site and the services we are able to offer. See the different category headings below to find out more or change your settings.

Allow all

Manage Consent Preferences

Strictly Necessary Cookies

Always active

These cookies are necessary for the website to function and cannot be switched off in our systems. They are usually only set in response to actions made by you which amount to a request for services, such as setting your privacy preferences, logging in or filling in forms. You can set your browser to block or alert you about these cookies, but some parts of the site will not then work.

Functional Cookies

These cookies enable the website to provide enhanced functionality and personalisation. They may be set by us or by third party providers whose services we have added to our pages. If you do not allow these cookies then some or all of these services may not function properly.

Performance Cookies

These cookies allow us to count visits and traffic sources so we can measure and improve the performance of our site. They help us to know which pages are the most and least popular and see how visitors move around the site.

Targeting Cookies

These cookies may be set through our site by our advertising partners. They may be used by those companies to build a profile of your interests and show you relevant adverts on other sites. If you do not allow these cookies, you will experience less targeted advertising.

Cookie List

Clear

Apply Cancel

Consent Leg.Interest

Confirm my choices

Power
