# Supplementary material for: KG-Microbe: Building modular and scalable knowledge graphs for microbiome and microbial sciences
Source: Gigascience. 2026 Jul 10;15:giag077. doi: 10.1093/gigascience/giag077 (PMC13536490; doi:10.1093/gigascience/giag077)
Supplement: Supplementary file 6 [file giag077_giga-d-25-00093_revision_2.pdf]

GigaScience

KG-Microbe - Building Modular and Scalable Knowledge Graphs for Microbiome and Microbial Sciences

--Manuscript Draft--

**Manuscript Number:**	GIGA-D-25-00093R2
**Full Title:**	KG-Microbe - Building Modular and Scalable Knowledge Graphs for Microbiome and Microbial Sciences
**Article Type:**	Research
**Funding Information:**	U.S. Department of Energy (DE-AC0205CH11231) — Dr. Marcin P. Joachimiak<br>U.S. National Library of Medicine (T15LM009451) — Dr. Brook E Santangelo<br>U.S. National Library of Medicine (R01LM013400) — Dr. Lawrence E. Hunter<br>National Center for Advancing Translational Sciences (OT2TR003422) — Dr. Lawrence E. Hunter

Abstract:

Background

The integration of many disparate forms of data is essential for understanding the microbial world and its interaction with the environment and human health. Doing so is particularly challenging in the context of microbe-host and microbe-microbe interactions that contribute to health or environmental outcomes. There are thousands of relevant microbial species, and millions of interactions among those microbes and with their environment or host. Integrated information (e.g., about host and microbial physiology, genetics, and metabolism) facilitates deeper understanding of complex mechanisms and helps interpret correlative results.

Results

The KG-Microbe construction framework is a novel approach to harmonizing bacterial and archaeal data in the form of a Findable, Accessible, Interoperable, Reusable (FAIR) and AI-ready knowledge graph (KG). Starting from a core KG with organismal traits, environments and growth preferences and the integration of established ontologies, the framework generates a hierarchy of related KGs targeting specific use cases, including the human microbiome in the context of disease, or environmental microbiomes. The framework supports customizable taxa subsets representing communities or clades of interest. Evaluations of the KG-Microbe KGs through a series of competency questions demonstrate the accuracy and effectiveness of the data harmonization, and the utility of the resulting KGs in studies of inflammatory bowel and Parkinson’s diseases. Finally, the predictive and environmental capabilities of the KGs are demonstrated by explaining growth preferences using graph features.

Conclusions

The KG-Microbe framework unifies microbial contexts in a single resource to support integrative analyses across biomedical, host, and environmental domains. KG-Microbe is a flexible, modular enabling technology for humans and machine learning methods to uncover mechanistic explanations of microbial associations.

**Corresponding Author:**	Brook E Santangelo<br>University of Colorado Anschutz Medical Campus<br>Aurora, CO UNITED STATES
**Corresponding Author Secondary Information:**	
**Corresponding Author's Institution:**	University of Colorado Anschutz Medical Campus
**Corresponding Author's Secondary Institution:**	
**First Author:**	Brook E Santangelo
**First Author Secondary Information:**	

Powered by Editorial Manager® and ProduXion Manager® from Aries Systems Corporation

**Order of Authors:**	Brook E Santangelo
	Harshad Hegde
	J. Harry Caufield
	Justin Reese
	Tomas Kliegr
	Lawrence E. Hunter
	Catherine A. Lozupone
	Christopher J. Mungall
	Marcin P. Joachimiak
**Order of Authors Secondary Information:**	
**Response to Reviewers:**	Manuscript Reviews:<br>—---------------------------------------<br>Reviewer #1: I thank the authors for their revisions and responses to my previous comments. The revised manuscript shows improvement in clarity and structure. However, the paper is quite long in its current form, which may affect readability. The authors might want to consider moving some of the more detailed content to the supplementary materials.<br><br>We appreciate this suggestion from the reviewer. We have moved this detailed content into the Supplementary Methods section 1.1-1.11.<br><br>Minor comments:<br>- Correct the reference in the sentence: "SHAP values for all features can be found in XXXX."<br><br>Thank you for catching this, we have revised the sentence.<br><br>- Regarding the sentence "Biological process, molecular function, and cellular component GO terms are extracted from ...", my earlier comment concerned sentence structure (not content). I suggest revising it to something like "GO terms for biological process, molecular function, and cellular component are extracted from ..."<br><br>We appreciate the stylistic recommendations, we have updated this sentence to: “GO terms for biological process, molecular function, and cellular component are extracted from the “go” field, excluding any text or brackets, and linked to microbes via the predicate “biolink:participates_in”, “biolink:participates_in”, or “biolink:located_in”, respectively.”<br><br>Reviewer #4:<br><br>-- Uploading files in the Github repository. The authors have added the necessary files to the GitHub or instructions for how to download them. However, However, several intermediate files required to reproduce the full workflow remain unavailable. For example, the intermediate file "Gold_Standard_Species_Overlap_butyrate_produces.csv" could not be reproduced due to computational limitations and was shared by the authors via email instead of being made publicly available.To allow independent reproduction of the analyses, including those in the kg-microbe-paper repository, we recommend that all intermediate files be shared in the public repository or that clear instructions be provided to generate them.<br>-- Updating the Readme file with environment information. The authors have updated the readme file with the required Python packages. But they did not add information on hardware requirements (for computers with 24 GB ram or less the script is not able to execute). And we encourage the authors to include the hardware requirements in the README file.<br><br>We appreciate the reviewer ensuring reproducibility here. We uploaded the mentioned

	intermediate file (Gold_Standard_Species_Overlap_butyrate_produces.csv) and added a statement in the ReadMe of how to access this file if the script is unable to run to completion in Step 6 of the ReadMe. In addition we improved the organization of the primary input and intermediate files, now all located in specific subdirs in data/. A few optimizations were added to the make file and python scripts and verified to produce the exact results. Here is the summary:<br><br>1. Package Management Migration<br><br>- Poetry → uv: ~10x faster dependency resolution and installation<br>- Reduced environment setup time from minutes to seconds<br><br>2. Memory Optimization<br><br>- Created Classification_gold_standard_comparison_optimized.py<br>- Fixes OOM errors on systems with <200 GB RAM<br>- Uses DuckDB in-memory queries instead of loading entire 62 GB dataset into pandas<br><br>3. Files Moved to data/Input_Files/<br><br>All primary input files are now organized as follows:<br><br>data/Input_Files/<br>├── Gold_Standard_Species_Overlap_butyrate_produces.csv (373 KB - tracked in git)<br>├── KGMicrobe-biomedical-function-20250222.tar.gz (4.3 GB - git-ignored)<br>├── kg-microbe-biomedical-function-cat/ (directory - git-ignored)<br>│ ├── merged-kg_edges.tsv (29 GB)<br>│ ├── merged-kg_edges_noEC.tsv (9.5 GB)<br>│ ├── merged-kg_edges_competency_specific_ec.tsv (9.2 GB)<br>│ ├── merged-kg_edges_ncbitaxon.tsv (45 MB)<br>│ └── merged-kg_nodes.tsv (15 GB)<br>├── ncbitaxon_nodes.tsv (130 MB - git-ignored)<br>└── equilibrator_cache/ (directory - git-ignored)<br>├── compounds.sqlite (130 MB - downloaded on first run)<br>└── cc_params.npz (50 MB - downloaded on first run)<br><br>4. Python Scripts Updated (12 files)<br><br>All references updated from ./data/ to ./data/Input_Files/ and from ./src/ to ./data/:<br><br>- src/Classification_gold_standard_comparison.py<br>- src/Classification_gold_standard_comparison_optimized.py<br>- src/Competencies.py (also added appdirs monkey-patch for equilibrator cache configuration)<br>- src/Functional_Annotations.py<br>- src/Functional_Annotations_EC.py<br>- src/Gold_standard_Competency_analysis.py<br>- src/Process_competency_questions.py<br>- src/classification.py<br>- src/classification_permutations.py<br>- src/classification_utils.py (fixed makedirs path)<br>- src/gut_microbes_competencies.py<br>- src/ncbi_phylogeny_search.py<br><br>5. .gitignore Updated<br><br>Added multiple exclusions:<br>- *.tar.gz - Exclude all tar archives<br>- data/Input_Files/* - Exclude all files in Input_Files<br>- data/Input_Files/*/ - Exclude all subdirectories in Input_Files<br>- !data/Input_Files/Gold_Standard_Species_Overlap_butyrate_produces.csv - Exception to track Gold Standard file

Powered by Editorial Manager® and ProduXion Manager® from Aries Systems Corporation

	- data/Input_Files/equilibrator_cache/ - Exclude equilibrator cache<br><br>6. Makefile Updated<br><br>- Updated clean target to remove data/Input_Files/equilibrator_cache<br><br>7. Equilibrator Cache Configuration<br><br>src/Competencies.py now includes:<br>- Patch of appdirs.user_cache_dir() to redirect equilibrator cache to data/Input_Files/equilibrator_cache/<br>- This ensures both compounds.sqlite and cc_params.npz download to the project directory<br><br>8. Documentation Updated<br><br>- README.md: Updated path references:<br>- './Phylogeny_Search' → './data/Phylogeny_Search'<br>- './src/Intermediate_Files' → './data/Intermediate_Files'<br>- Added note about equilibrator cache auto-download<br><br>9. Final Directory Structure<br><br>data/<br>├── Input_Files/ # All downloaded/provided input files (mostly git-ignored)<br>│ ├── Gold_Standard_*.csv # Tracked in git<br>│ ├── *.tar.gz, *.tsv # Git-ignored downloads<br>│ ├── kg-microbe-biomedical-function-cat/ # Git-ignored KG files<br>│ └── equilibrator_cache/ # Git-ignored equilibrator cache<br>├── Intermediate_Files/ # Processing results (tracked in git)<br>│ ├── HMP_Microbes_Mapped.csv<br>│ ├── HMP_Microbes_Mapped_Summary.csv<br>│ ├── IBD_Classification_butyrate_producers_summary.csv<br>│ └── PD_Classification_butyrate_producers_summary.csv<br>├── Intermediate_Files_Competencies/ # Competency analysis (tracked in git)<br>├── hmp_supplementary/ # HMP supplementary Excel files (tracked in git)<br>├── ML_model_shap/ # SHAP feature importance (tracked in git)<br>└── Phylogeny_Search/ # Cached taxonomy (tracked in git)<br><br>10. Key Improvements<br><br>a. Complete path consolidation: All data files now under data/, no references to src/ data directories<br>b. Equilibrator cache control: Downloads to project directory instead of home directory (platform-agnostic)<br>c. Documentation path updates: README.md and CLAUDE.md fully synchronized<br>d. Proper git tracking: Small reference files tracked, large downloads excluded<br>e. Generated outputs committed: 156 analysis output files (~80 MB) now tracked in git<br><br>-- Add metadata to the dataset. The information provided by the authors mainly describes the data processing workflow and file structure, not the metadata describing the datasets. For better transparency and FAIR compliance, we recommend that the authors include explicit metadata documentation (for example, a data dictionary or metadata table describing each dataset, variable names, formats, and data provenance).<br><br>We appreciate the suggestion. Section 2, Data Description, gives a high level explanation of each resource and its content. In Section 6.2 is Table 1, which describes the type of data included for each dataset. These descriptions ensure that we've explained the purpose of each resource and how it fits into the broader KG resource. Finally, we have moved the detailed documentation of how we used each dataset, variable names, and formats to Supplementary Methods 1.1-1.11 to ensure that all metadata included from each dataset is clear.

	- Summary of the second computational reproducibility review<br>The results of the original study were reproduced during the first review with a minor numerical error. The result was consistent for inflammatory bowel disease, but differed slightly for Parkinson's disease (PD):<br>- The reproduced p-value for PD was 8e-293 as opposed to the reported p-value of 2e-288<br><br>- The reproduced value of the test statistic for PD was 1337 whereas the reported value was 1317.<br>These numerical discrepancies are minor. We verified the chi-squared results with the reported contingency table (figure 6C) in R and obtained the same chi-squared statistic and p-value as reported in the manuscript, which suggests that the difference observed in the first review likely resulted from an issue when running the original code to generate<br>the contingency table. However, as intermediate files are not shared in the repository, we<br>were unable to re-run the complete analysis to confirm the exact source of the discrepancy. We therefore recommend that the authors double-check this part of the analysis to ensure full computational reproducibility.<br><br><br>We greatly appreciate the reviewer's thorough investigations of the reproducibility of this analysis. We recognize slight numerical differences may arise due to data and software dependencies changing over time, though we ensured that the results themselves as well as the scientific interpretation of these results have not changed given these p-value and chi-square statistics. We determined that these changes are due to updates to the NCBITaxon owl file through the OwlReady package, which is accessed in the 4th script of the pipeline (Classification_gold_standard_comparison.py), and updates to the Equilibrator API in the second script of the pipeline (Process_competency_questions.py). In addition extreme p-value calculations may have platform and package version-specific sensitivity. In the latest reproduction, one less extreme p-value matched exactly; the other (more extreme) p-value shifted by 5 in the base-10 exponent. We made additional code edits to further optimize the competencies and also cache the Equilibriator results.<br><br><br>We appreciate this reviewers' detailed analysis of the change of these results. After reviewing the results reported in the manuscript and our own analysis, we confirm that these are correctly stated in the manuscript.<br><br>Author Decision Letter<br><br>In addition, please register any new software application in the bio.tools and SciCrunch.org databases to receive RRID (Research Resource Identification Initiative ID) and biotoolsID identifiers, and include these in your manuscript. Computational workflows should be registered in workflowhub.eu and the DOIs cited in the relevant places in the manuscript. These will facilitate tracking, reproducibility and re-use of your tool.<br><br>The KG-Microbe framework has been registered at these resources:<br>1.https://rrid.site/data/record/nlx_144509-1/SCR_026589/resolver<br>2.https://bio.tools/kg-microbe<br>3.https://workflowhub.eu/workflows/2044<br>4.https://doi.org/10.48546/WORKFLOWHUB.WORKFLOW.2044.1<br><br>All web links and URLs should be given a reference number and included in the reference list rather than within the text of the manuscript. Please remove the URLs, cite them as reference and adjust the order of the reference accordingly. Except for "Abstract" and "Availability of Source Code and Requirements"<br><br>We thank the editor for noticing this. We have updated all citations and removed any URLs from the main manuscript in the relevant sections.
**Additional Information:**	

Question	Response
Are you submitting this manuscript to a special series or article collection?	No
**Experimental design and statistics**<br><br>Full details of the experimental design and statistical methods used should be given in the Methods section, as detailed in our Minimum Standards Reporting Checklist. Information essential to interpreting the data presented should be made available in the figure legends.<br><br>Have you included all the information requested in your manuscript?	Yes
**Resources**<br><br>A description of all resources used, including antibodies, cell lines, animals and software tools, with enough information to allow them to be uniquely identified, should be included in the Methods section. Authors are strongly encouraged to cite Research Resource Identifiers (RRIDs) for antibodies, model organisms and tools, where possible.<br><br>Have you included the information requested as detailed in our Minimum Standards Reporting Checklist?	Yes
**Availability of data and materials**<br><br>All datasets and code on which the conclusions of the paper rely must be either included in your submission or deposited in publicly available repositories (where available and ethically appropriate), referencing such data using a unique identifier in the references and in the "Availability of Data and Materials" section of your manuscript.	Yes

Have you have met the above requirement as detailed in our Minimum Standards Reporting Checklist?	
GigaScience has policies and guidelines in place for the use of generative AI-writing tools such as ChatGPT. If you have used such writing tools to assist with writing the manuscript this must be declared and cited in the text. Authors should not list AI-writing tools and other AI-assisted technologies as an author or co-author and should acknowledge that they are fully responsible for text generated or refined by AI-writing tools.<p><br><br>A summary of use (particularly in the introduction or among methods) needs to be included at the end of the paper, and the outputs should also be included as a supplementary file hosted in GigaDB or other open repositories. Please read our guidelines for more information. <p><br><br>By submitting to GigaScience, you are aware of the journal's AI-writing tools policy, and if you have declared use of such tools below, you have acknowledged this where appropriate in your manuscript and have made a summary of use and outputs available. <p><br><b>AI-assisted writing tools have been used in the preparation of this manuscript?	No

Powered by Editorial Manager® and ProduXion Manager® from Aries Systems Corporation

Manuscript

Click here to access/download;Manuscript;KG-Microbe_paper_REVISIONS2.docx

KG-Microbe - Building Modular and Scalable Knowledge Graphs for Microbiome and Microbial Sciences

Brook E. Santangelo[1*], Harshad Hegde[2#], J. Harry Caufield[2], Justin Reese[2], Tomas Kliegr[3], Lawrence E. Hunter[4], Catherine A. Lozupone[1], Christopher J. Mungall[2], Marcin P. Joachimiak[2*]

[1] Department of Biomedical Informatics, University of Colorado Denver Anschutz Medical Campus, Aurora, CO, USA
[2] Environmental Genomics and Systems Biology, Lawrence Berkeley National Laboratory, Berkeley, CA 94720, United States
[3] Faculty of Informatics and Statistics, Prague University of Economics and Business, Prague, Czech Republic
[4] Department of Pediatrics, University of Chicago, Chicago, IL, USA
[#] Now at GlaxoSmithKline, Inc.

* Correspondence: brook.santangelo@cuanschutz.edu, marcinjoachimiak@gmail.com

Abstract

Background

The integration of many disparate forms of data is essential for understanding the microbial world and its interaction with the environment and human health. Doing so is particularly challenging in the context of microbe-host and microbe-microbe interactions that contribute to health or environmental outcomes. There are thousands of relevant microbial species, and millions of interactions among those microbes and with their environment or host. Integrated information (e.g., about host and microbial physiology, genetics, and metabolism) facilitates deeper understanding of complex mechanisms and helps interpret correlative results.

Results

The KG-Microbe construction framework is a novel approach to harmonizing bacterial and archaeal data in the form of a Findable, Accessible, Interoperable, Reusable (FAIR) and AI-ready knowledge graph (KG). Starting from a core KG with organismal traits, environments and growth preferences and the integration of established ontologies, the framework generates a hierarchy of related KGs targeting specific use cases, including the human microbiome in the context of

disease, or environmental microbiomes. The framework supports customizable taxa subsets representing communities or clades of interest. Evaluations of the KG-Microbe KGs through a series of competency questions demonstrate the accuracy and effectiveness of the data harmonization, and the utility of the resulting KGs in studies of inflammatory bowel and Parkinson's diseases. Finally, the predictive and environmental capabilities of the KGs are demonstrated by explaining growth preferences using graph features.

Conclusions

The KG-Microbe framework unifies microbial contexts in a single resource to support integrative analyses across biomedical, host, and environmental domains. KG-Microbe is a flexible, modular enabling technology for humans and machine learning methods to uncover mechanistic explanations of microbial associations.

Keywords: knowledge graphs, ontologies, microbiology, microbiome, phenotypes, genome annotations, semantic path analysis, machine learning

1. Background

Our knowledge of the influence of microbes in varied environments has expanded significantly due to technological advances. The ability to identify organisms across diverse taxonomic groups using efficient sequencing technologies has allowed us to survey many environments and hosts. These groups, known as taxa, can range from species to higher-level classifications such as families or genera. Identifying organisms at the lowest classification levels, the species or strain level, provides the most resolved genomic content from direct experimental observations. All of this knowledge is critical for understanding microbial organisms and their communities by uncovering differences in populations of taxa and their associated molecular functions, such as the gut microbiome of a disease versus a healthy population[1,2].

Sequence-based methods for understanding microbial function either only cover a specific set of taxa or predict function at potentially coarse taxonomic resolution, such as the family or phylum level[1,3]. Attributing functions at more specific taxonomic resolution can be done by considering taxonomic relationships (e.g., between species and strains) between annotated and unannotated microbes. For example, one critical function of microbes with implications for health and disease is the production of short-chain fatty acids (SCFAs). Evidence that these microbial metabolites reduce inflammation suggests the protective nature of SCFA-producing microbes against inflammatory disease[4]. Understanding this function at a per-strain or species level is important for characterizing a healthy microbiome and distinguishing it from disease states, yet remains difficult given the fragmented nature of microbial knowledge[5].

Information about the structure and function of microbes and their communities is available in a range of sources. Microbial traits and functional attributes can be discovered with laboratory experiments or computational predictions using publicly available data[6]. However, relevant information is spread across many special-purpose databases, which vary greatly in structure and format[5]. A knowledge graph (KG) is an effective and flexible way to integrate this vast amount of information, consistently representing knowledge despite varying formats, sources, and domains of the original data sources. KGs often rely on ontologies, which are expert curated, hierarchical summaries of concepts in a specific domain, e.g. biological metabolites, that support standardized knowledge representation. In the biomedical sciences, KGs have gained popularity in applications such as drug discovery and repurposing as well as disease classification[7,8]. The inherent data interoperability of KGs presents a unique benefit to the study of microbial communities as a rapidly evolving field that is still undergoing harmonization. Applying a KG approach to microbiome related research questions is in its early stages, as existing microbial KGs represent targeted aspects of microbiology or human disease[9–12]. These resources have demonstrated the potential for KGs to enhance microbiome research through answers to queries for broad biomedically and

environmentally relevant information. However, no resource exists that harmonizes microbial traits and genomic annotations while seamlessly integrating taxonomic relationships.

To address this gap, we developed the KG-Microbe framework which supports the generation of large and comprehensive KGs with relevant information about microbes and their interactions with the environment and human health. The KG-Microbe framework can ingest data from a broad array of sources, ranging from ontologies to published datasets to reference databases, to fully represent these interactions. KG-Microbe enhances microbial and microbiome research through representation of microbial morphology, traits, ecology, function, and taxonomy. As very large KGs can be computationally demanding, the KG-Microbe framework enables the construction of a set of KGs, fit for purpose in varied contexts by including only relevant information for a particular use case. The quality of KG-Microbe KGs is demonstrated through graph competency queries, which showcase the variety of data sources and the relevance of content within the graph, and its effectiveness is demonstrated through its ability to enhance our functional understanding of associations with inflammatory bowel disease (IBD) and Parkinson's disease (PD). We also demonstrate the utility of KG-Microbe in environmental contexts, using KG features for training machine learning models to predict and explain temperature growth preferences, leading to new biological insights. The KG-Microbe framework can enable the microbiology and microbiome fields by harmonizing and standardizing microbial data, facilitating insights and predictions about microbes and their communities.

2. Data Description

2.1. KG-Microbe Content

The KG-Microbe framework supports the integration of microbial trait and function data from curated databases, ontologies, and experimental results. Because computation and memory are limiting factors, the framework supports the construction of custom KGs that harmonize data sources relevant to applications of microbiology or biomedicine, otherwise known as modules

(Figure 1). We present four custom KGs covering different applications in biomedicine and more general microbiology. The core KG, KG-Microbe-Core, designed to contain a foundational set of broadly applicable microbiological knowledge, includes microbial organismal traits obtained from structured databases including literature curation and experimental assay results. KG-Microbe-Biomedical introduces disease relevant concepts and human functional information. KG-Microbe-Function and KG-Microbe-Biomedical-Function also ingest microbial functional information (Figure 1A). All KG-Microbe graphs are modeled according to the Biolink Model, a formalized semantic model for the representation of complex biological data[13]. The KG-Microbe framework provides software infrastructure to build the four graphs, which are made available in KG-Hub and the KG-Registry[14,15]. Knowledge of microbial traits and functions was ingested from a variety of resources and modeled to serve the purposes of mechanistic inference in different research domains (Figure 2). In contrast to other microbial KGs, KG-Microbe includes only structured or semi-structured data, as opposed to mining free text, to ensure only the most accurate representation from curated sources (Supplementary Table 1). Negative assertions are currently not encoded in KG-Microbe (e.g., ‘does not metabolize X’), and therefore the KG does not distinguish between absence of data and negative assertions.

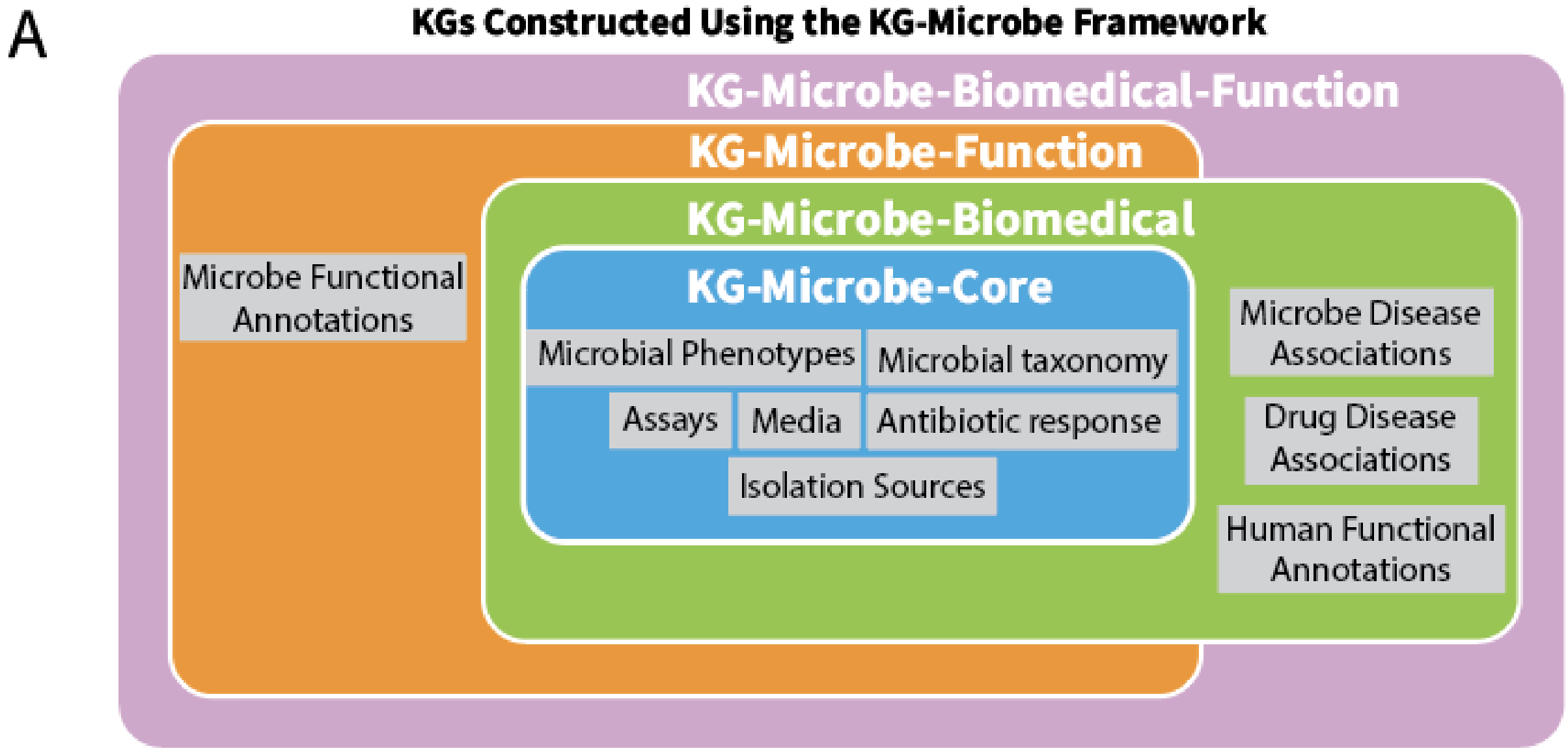

B

	Number Nodes	Number Edges	Size Edges File
**KG-Microbe-Core**	1.4 million	3.0 million	362 MB
**KG-Microbe-Biomedical**	1.6 million	5.4 million	663 MB
**KG-Microbe-Function**	151.7 million	555.5 million	29 GB
**KG-Microbe-Biomedical-Function**	152.0 million	557.9 million	29 GB

Figure 1. (A) Content of each KG generated with the KG-Microbe framework. (B) The size of each KG, listed as total number of nodes, total number of edges, and the size of the edges file. Each KG in the framework can be used as a starting point to construct novel custom KGs.

2.1.1. Microbial Taxonomy and Strain Representation

The value of KGs produced by the KG-Microbe framework depends on the accurate representation of microbial knowledge at all taxonomic levels. KG-Microbe-Core includes 1,012,214 microbial taxa nodes, 870,981 of which are normalized to National Center for Biotechnology Information (NCBI) Taxonomy terms[16]. The remaining 159,680 taxa without a corresponding NCBI Taxonomy term are introduced as children of their related species-level NCBI Taxonomy term. The inclusion of the full taxonomic hierarchy supports summarization of traits at higher taxonomic levels or exploration of traits at lower taxonomic levels, using the structured relationships in the taxonomic hierarchy.

Traits and genomic annotations are linked to microbial nodes at a species or strain level. Strains are normalized to NCBI Taxonomy when a mapping to an NCBI Taxonomy node exists in the source. This sometimes results in a rollup of these attributes to the species level, where multiple strain level traits are attached to a species level node. In these cases, the strain nodes are still introduced to the KG as children of their related species, and the provenance of that trait-strain relationship remains in the graph metadata, for example *"strain:CCUG-59342"*, a

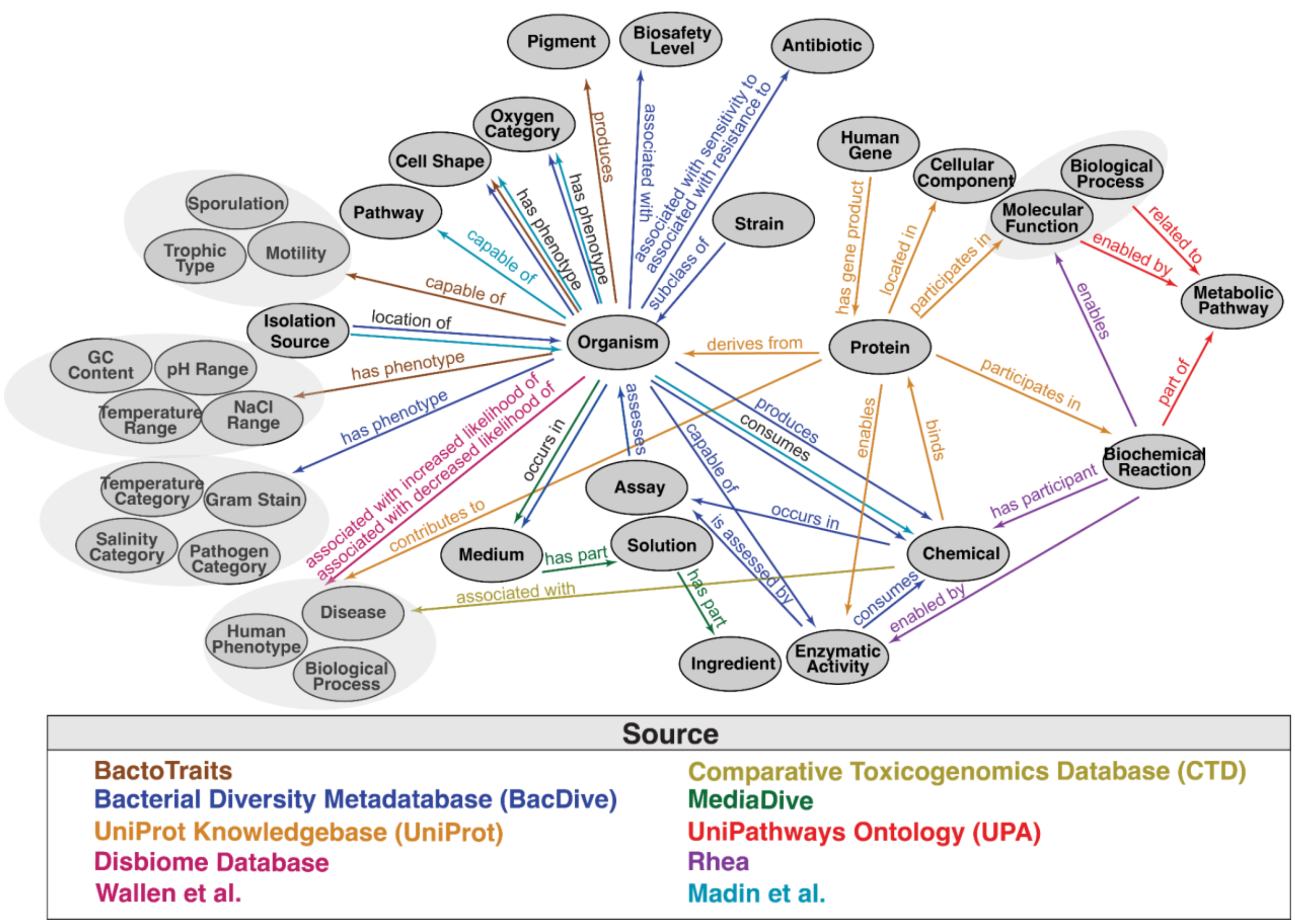

Figure 2. Semantic schema supported by the KG-Microbe framework, representing all sources across all four KG-Microbe KGs.

strain, is a subclass of *Escherichia coli (NCBITaxon:562)*, the species to which all traits are linked. When no mapping to an NCBI Taxonomy node exists in the source, traits are linked directly to a corresponding strain level node in the KG. This modeling choice supports the consistency and robustness of taxa representation, with traits linked to standardized terms for

microbial data that is not strain-resolved by identifier. This also supports efficient queries when mapping external data sources to the knowledge represented in the graph.

2.1.2. Microbial Phenotypes

Microbial phenotypes are ingested from three sources: the Bacterial Diversity Metadatabase (BacDive), the BactoTraits database, or a published dataset that unifies various sources of information about microbiology (Madin et al)[17–19]. The Bacterial Diversity Metadatabase (BacDive) is one of the largest standardized resources of prokaryotic information from over 80,000 strains[20]. The BactoTraits database expands on BacDive cultured bacteria experimental results by modeling 19 physiologic and morphological traits[18]. Madin et al. is an aggregated dataset of over 170,000 strains and 15,000 species curated from a wide range of sources including publications and textbooks[19]. Each of the microbial phenotype nodes are attached to a species or strain level taxon node (Figure 2).

The microbial phenotypes modeled in the KG framework include environmental growth information, morphological qualities, trophic types, ecological phenotypes, isolation sources, and several forms of microbial metabolism. Direct microbe-chemical links indicate whether a microbe is a producer or consumer of a given chemical. When ingested from BacDive or Madin et al., this knowledge comes from culture observations of carbon substrates or published literature, serving as a developing gold-standard of microbial metabolism. Antibiotic resistance and sensitivity data is sourced from antibiogram results in BacDive. All antibiotic compounds are mapped to ChEBI nodes, which consist of 165 unique chemicals to which microbes are sensitive to and 155 unique chemicals to which microbes are resistant. Finally, the involvement of species or strains in pathways or capabilities to perform enzymatic activities are introduced from culture observations or structured databases.

2.1.3. Medium and Assay Entities

We modeled a series of assay results called Analytical Profile Index (API) assays which assess a series of physiological traits from Bacdive[17]. 461 unique assays from which the culturing results are sourced in Bacdive are introduced according to the microbe that each assay tests. Information about media was also introduced from MediaDive, an expert curated database of over 3,000 distinct cultivation media recipes for over 40,000 strains that are linked to the strains represented by BacDive[21]. From this source, we introduce 3,333 unique media in which microbes can grow. The chemical solutions that make up each medium are then modeled as unique nodes that have chemical parts. All of this information is valuable to aid in understanding microbial biology in the context of nutrition requirements and growth preferences.

2.1.4. Functional Annotations

Functional annotations for 32,662 microbes are ingested by the KG-Microbe framework from the UniProt Knowledgebase (UniProt)[22] . Due to its large size (Figure 1B), UniProt data is only ingested into KG-Microbe-Function and KG-Microbe-Biomedical-Function. UniProt is a leading community and expert curated database of both reviewed and computationally predicted functional annotations of proteins for a large number of organisms[22]. UniProt is updated frequently and thus provides comprehensive and up to date functional annotations critical for understanding microbial mechanisms. KG-Microbe ingests functional annotations by organism (NCBITaxon) proteomes, which is a set of proteins encoded by the genes that are present as complete or draft genome, when the proteome contains a threshold number of proteins (see Methods). The protein node is linked directly to the corresponding NCBI Taxonomy node, then linked to the chemicals to which the protein binds, biological processes or molecular functions that the protein participates in, cellular components that the protein is located in, enzymatic activity that the protein is capable of, and biochemical reactions that the protein participates in (Figure 2). Enzymatic activities are mapped to Enzymatic Commission (EC) numbers, which provide a hierarchical structure that

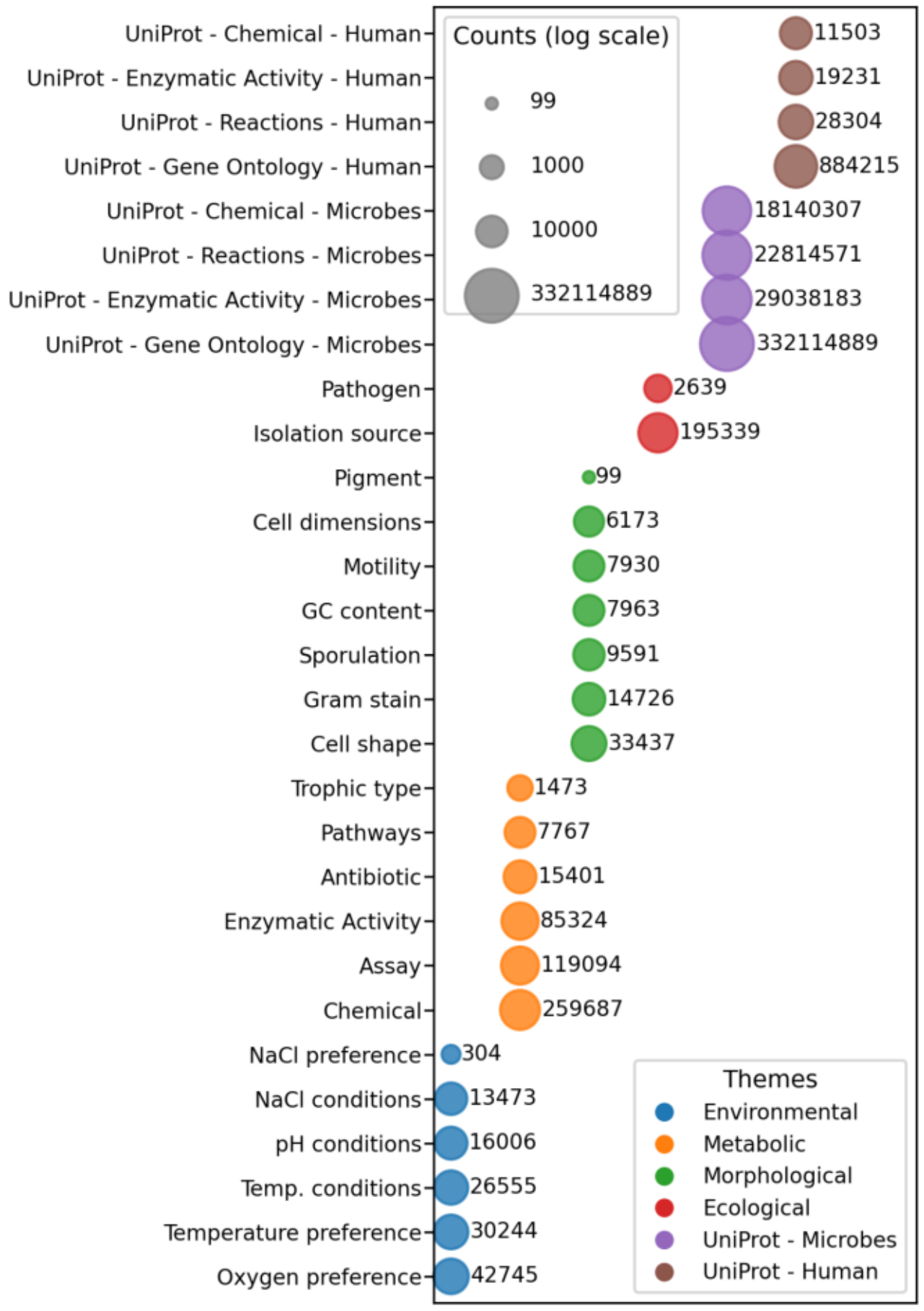

Figure 3. Summary of organismal traits and themes in KG-Microbe. The y-axis represents ontology or custom prefixes corresponding to different traits. Each trait prefix can have many different values and the numbers in the plot represent the count of taxa which have any value with that prefix. Traits with fewer than five edges are not shown. KG-Microbe has annotations for 1,773 organismal trait terms, 53,786 functional annotation terms linked to microbes, and 33,305 functional terms linked to human proteins in the current KG-Microbe graphs.

categorizes enzymes by function. Biochemical reactions are mapped to IDs from the Rhea database, an expert-curated knowledge base of reactions and their relationships to chemical substrates and products. Molecular function and biological processes are mapped to IDs from the Gene Ontology (GO). By representing protein function across all three of these similar sources, none of the curated information from UniProt about protein function is lost. Using these standardized data resources ensures cross-database compatibility of the represented knowledge.

Pathway information is also represented using the Unipathways ontology, a manually curated resource of metabolic pathways that provides relationships between biochemical reactions, enzymatic activity, and biological processes or molecular functions[23]. In the KG, the information is abstracted to the molecular function that is enabled by the pathway, the synonym biological process for each pathway when available, and the biochemical reactions that are part of each pathway. This semantic representation of genome annotations, within the context of inter-linked reaction, chemical, function, and pathway hierarchies, provides a standardized way to formally define microbial protein function, where provenance is maintained by including all relevant function node types.

2.1.5. Biomedically Relevant Insights: Drug, Disease, and Human Content

Disease and human-host relevant information was harmonized into the biomedical KG-Microbe graphs for applications in understanding the involvement of the host microbiome in disease. The human subset of functional annotations from UniProt are introduced to KG-Microbe-Biomedical and KG-Microbe-Biomedical-Function, which resulted in 163,341 new protein nodes. The gene templates of human proteins are also introduced from UniProt[24]. This supports a combined insight into host biology and microbiology in pursuit of exploring the host-associated microbiome. Human diseases and their relationships to microbes are introduced from two resources: Disbiome and a published set of differentially abundant taxa from a population study of PD, referred to as Wallen et al.[25,26]. Each source provides correlative relationships between a taxon and a disease. Disbiome is a database of published information about microbial composition in disease and health states for diseases or human phenotypes[25]. A total of 4,209 unique associations are introduced between microbes that are increased in disease-associated gut microbiomes and their corresponding diseases or phenotypes, and 4,618 unique associations for those that are decreased. Chemical-disease associations are sourced from the Comparative Toxicogenomics Database (CTD)[27]. With this human physiology and disease knowledge, KG-Microbe-Biomedical and KG-Microbe-Biomedical-Function support biomedically relevant inference.

3. Analyses

3.1. Examining KG-Microbe Content through Competency Questions

The integration of vast amounts of information included in the KG-Microbe framework can include errors or omissions. We assess the accuracy of KG-Microbe-Function demonstrating four main qualities: (1) the graph's ability to synthesize multiple sources of information, (2) the graph's ability to infer microbial trait and function across taxonomic relationships, (3) the accessibility of complex knowledge from the graph enabled by its formal and computationally interoperable

representation, and (4) the relevance of the graph content to biomedical applications of the host-associated microbiome as well as general microbiology.

We first examine the extent to which the KG includes biomedically relevant taxa via the following competency question: *Does the taxonomic coverage of the KG include well studied gut microbes?* This competency assesses which taxa that are known to exist in the gut microbiome are also in the KG. The NIH Human Microbiome Project (HMP), a well-established reference dataset for microbial taxa of interest for human health, evaluated the microbiome of 300 healthy American adults to build a catalog of microbial taxa and genome sequences and define common metabolic pathways[28]. We used taxa from stool samples that were found to be significantly associated with host phenotypes such as age, gender, ethnicity, and other clinical metadata. Of the 75 unique taxa, 72 exist in KG-Microbe. The three that do not exist do not have a direct mapping to a taxon in NCBI Taxonomy, suggesting an unpublished taxon not yet in public databases, or a revision in the name. Thus, KG-Microbe effectively includes taxa of interest from this HMP resource.

We next sought out to evaluate whether the organismal traits of these taxa were represented in the KG via the following competency question: *Can you use the KG to learn about the complex traits of well-studied gut microbes?* We evaluated how many of the 75 HMP taxa previously described have at least one organismal trait in the KG based on the traits described in Figure 3[28]. Only taxa with a species rank were relevant to this competency as organismal traits for higher taxonomic ranks taxa are not included in available data resources. We found that 19 of the 21 species taxa had an organismal trait, suggesting the usefulness of using the KG to study well-known gut-related microbes.

We were also interested in whether functional annotations of these taxa were represented in the KG and used the following competency question to inquire: *Can you explore the functional annotations of well-studied gut microbes using the KG?* Using the same set of 75 taxa from HMP, we found that 18 of the 21 taxa of rank species had a functional annotation, all of which also had

an organismal trait[28]. This highlights the relevance of the taxa represented in the KG to human health and suggests the utility of KG-Microbe to provide more context about organismal traits and genome functions for the gut microbiome.

To provide more functional depth to the queries surrounding the knowledge included in KG-Microbe, our final competency question was designed to examine a particularly important health-related microbial trait: *Can you explore butyrate production based on the combined trait and functional representations?* The intestinal mucosa is an important barrier separating commensal microbes from the host. Short chain fatty acids (SCFAs) are made from nondigestible carbohydrates by microbial fermentation in the human gut. Butyrate is one of several SCFAs that have been shown to play an important role in strengthening the intestinal barrier[29]. Butyrate participates in several main signaling pathways critical to epithelial barrier function and immune homeostasis, including the AMPK and Akt signaling pathway that facilitates tight junction assembly and the activation of G-protein-coupled receptors to induce T cell-independent IgA secretion in the colon[30].

To understand the representation of butyrate production in KG-Microbe-Function, we evaluated specific paths, i.e. series of linked nodes and edges, in the KG that model biological mechanisms that are herein referred to as *semantic representations* (Figure 4A). By capitalizing on the unique capabilities of a KG to model diverse data types, semantic representations offer a powerful methodology for accessing knowledge. We chose three semantic representations from two sources, BacDive and UniProt, that each describe butyrate production in different ways. The first semantic representation is from BacDive organismal traits, reporting that a given microbe produces butyrate. Another semantic representation uses biochemical reactions (as Rhea nodes) that involve butyrate as a participant from UniProt protein annotations for a given microbe. To predict reaction direction and specify butyrate as a product rather than a participant, we used the eQuilibrator method (see Methods)[31]. The final semantic representation includes sets of enzymatic activity annotations (as EC nodes) for proteins from a given microbe that are involved

in one of four butyrate producing pathways (the acetyl-CoA, glutarate, 4-aminobutyrate, and lysine pathways, Figure 4A), which were identical EC sets to those used in a butyrate pathway prediction analysis to which we compared the microbial taxa resulting from our competency queries (referred to as Vital et al.)[32] While the content of the two semantic representations from UniProt is similar, we explore the differences in protein annotations with biochemical reactions (Rhea) vs. enzymatic activity (EC). The inconsistencies of the resulting butyrate-producing microbial set from each semantic representation exemplifies the need for data harmonization to capture all knowledge.

We developed queries that investigate the representation of a critical biological function of gut microbes: butyrate production. The approach to assessing the graph's representation of this biological function demonstrates the KG's ability to portray a well-studied aspect of the microbiome. For this competency, we compared the set of taxa reported to produce butyrate across these three semantic representations in KG-microbe, as well as those found with a sequence-based approach that serves as an external gold standard of butyrate producers not ingested in the KG. This analysis assesses the correctness of information represented in the KG, where we would expect alignment of the taxa identified through semantic representations to those in the gold standard. We evaluated a pathway prediction analysis performed by Vital et al. In that paper, genomes were analyzed from the Integrated Microbial Genome (IMG) database to find potential butyrate producers according to EC annotations from KEGG and a hidden Markov model (HMM)-based approach that inferred homologous sequences trained from protein sequences in NCBI of bacteria known to produce butyrate[32,33]. This method predicted 225 microbes capable of butyrate production, of which 216 could be mapped to NCBI Taxonomy identifiers[32] and 181 had protein annotations from UniProt in KG-Microbe-Function.

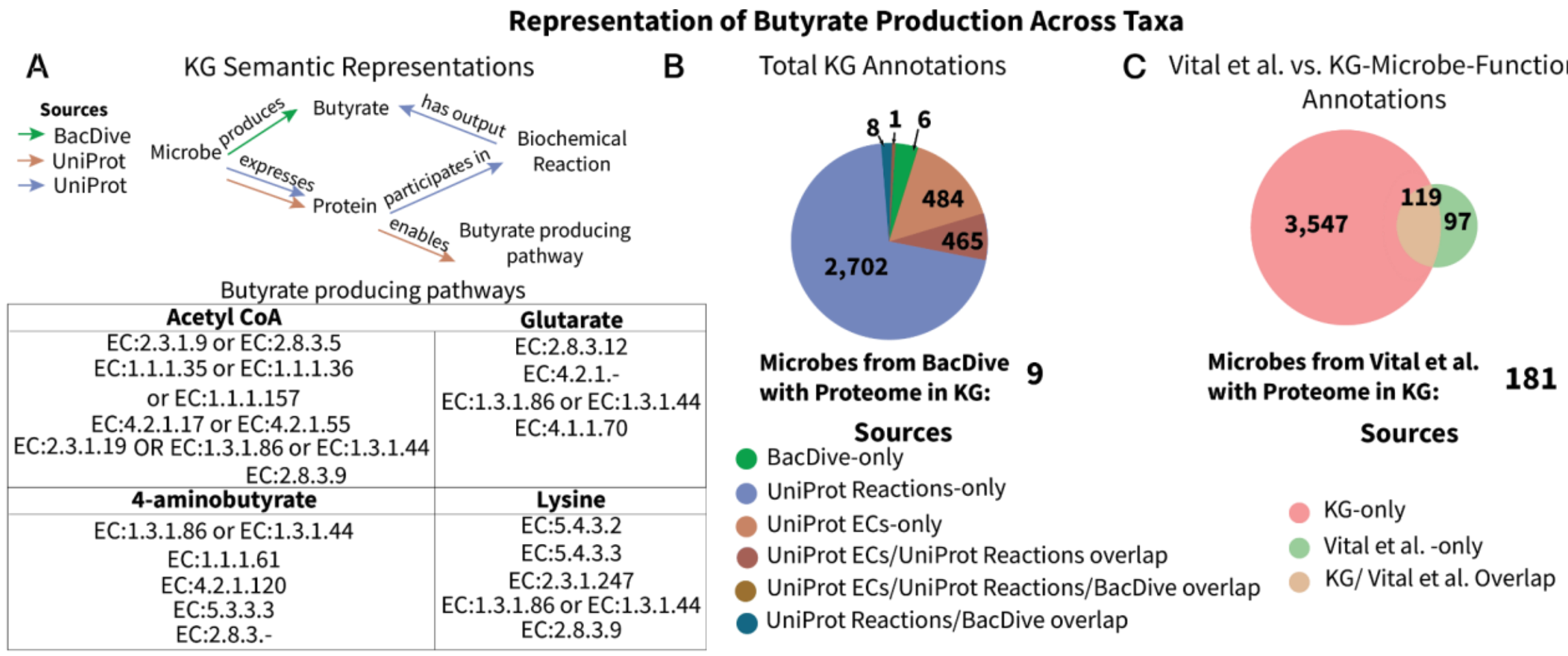

Figure 4. (A) Semantic representation of butyrate production in KG-Microbe-Function. BacDive provides organismal traits specifying which microbes can produce butyrate, and UniProt provides functional annotations through either reactions that have butyrate as a product (predicted using eQuilibrator) or through a curated set of EC numbers that are involved in on of 4 butyrate producing pathways. These sets of ECs with qualifiers represent the KG queries that were performed. (B) Number of unique microbes that are annotated to produce butyrate based on the semantic representations from (A). The number of taxa from the BacDive set that have UniProt annotations in the KG are also noted. (C) Number of unique microbes that are annotated to produce butyrate in Vital et al. (green) or through any representation from (A) (pink). The number of microbes from the Vital et al. set that have UniProt annotations in the KG are also noted.

A majority of the butyrate producers in the KG were from the semantic representation involving biochemical reactions (3,176 total), followed by the semantic representation involving enzymatic activity (949 total, Figure 3B). Very few direct butyrate production annotations from BacDive exist, suggesting room for an experimentally validated gold standard for this trait (15 total, Figure 3B). All microbes in the BacDive set that had butyrate-related functional annotations in the KG were also expected to produce butyrate based on the reaction or enzymatic activity semantic representation. A Monte Carlo simulation of 1,000 iterations ($p < 1e-4$) indicated that this overlap was statistically significant and not due to random chance. Thus, we show the accuracy of the taxa from UniProt having functional annotations that align with BacDive experimental data.

In comparing the KG butyrate producers to the set in Vital et al., we found that 119 of the 216 taxa (55%) according to Vital et al. were identified using these semantic representations in KG-Microbe-Function. Of the 181 taxa from Vital et al. with functional annotations in the graph set, 117 (65%) were represented in the KG as producing butyrate (the other 2 that overlap with the Vital et al. set were from BacDive, Figure 4C). To understand the predicted set of butyrate producers from KG-Microbe-Biomedical-Function, we examined the taxonomic groups annotated by this trait. We compared taxa that are in the KG and annotated to produce butyrate but not in Vital et al. (3,547 total taxa, Figure 4C) to those that are in Vital et al. but not annotated in the KG (97 total taxa, Figure 4C). In this comparison, while Vital et al.-exclusive taxa represent potential false negatives (annotations reported by Vital et al. but not captured by KG-Microbe), given the continual accumulation of new data and differences in scope between resources, some of these discrepancies may reflect differences in methodology or database coverage. A majority of the phyla and families represented in Vital et al. are covered in the KG (Figure 5A) even though there exist 97 taxa from Vital et al. that do not have a KG representation of butyrate production. A majority of the taxa annotated to have this trait by KG-Microbe-Function are also part of the primary phyla present in the human gut: Bacteroidota, Bacillota (Firmicutes), Actinomycetota (Actinobacteria), Pseudomonadota (Proteobacteria), or Fusobacteriota (Fusobacteria), though not all species or strains of that phyla are expected to survive in the human gut (Figure 5A)[2]. These results confirm alignment of taxa at coarser taxonomic resolution (at the phylum level) between the KG and Vital et al. despite having differences at the strain or species level. Over time, such discrepancies between prior published results and reference data resources are expected to arise from the continued accumulation of new data, which is captured in frequently updated databases such as NCBI and UniProt.

To further assess the accuracy of the KG knowledge, we evaluated the potential for the graph to identify key butyrate producing families in the human gut, including Ruminococcaceae, Lachnospiraceae, Bacteroidaceae, Prevotellaceae, Clostridiaceae, Oscillospiraceae, and

Eubacteriaceae[34]. We found that 100% of species and strains in the genera *Faecalibacterium* and *Roseburia* were predicted to be butyrate producers, and 50% of species and strains in the genus *Oscillobacter*.

Through the comparison of semantic representations in the KG, an external gold standard, and what we know about taxa present in the human gut microbiome, we found alignment at the family and phylum levels. We have demonstrated that the large number of taxa represented in the KG though not by the Vital et al. analysis are aligned with our understanding of butyrate production in the gut. The species and strain level differences that arise between Vital et al. and the KG allude to the variance in functional annotations, which is expected with manual curation and predictions. This semantic representation methodology thus demonstrates the ability to summarize microbial function according to the modeling of reaction and pathway information alongside phylogenetic relationships within KG-Microbe.

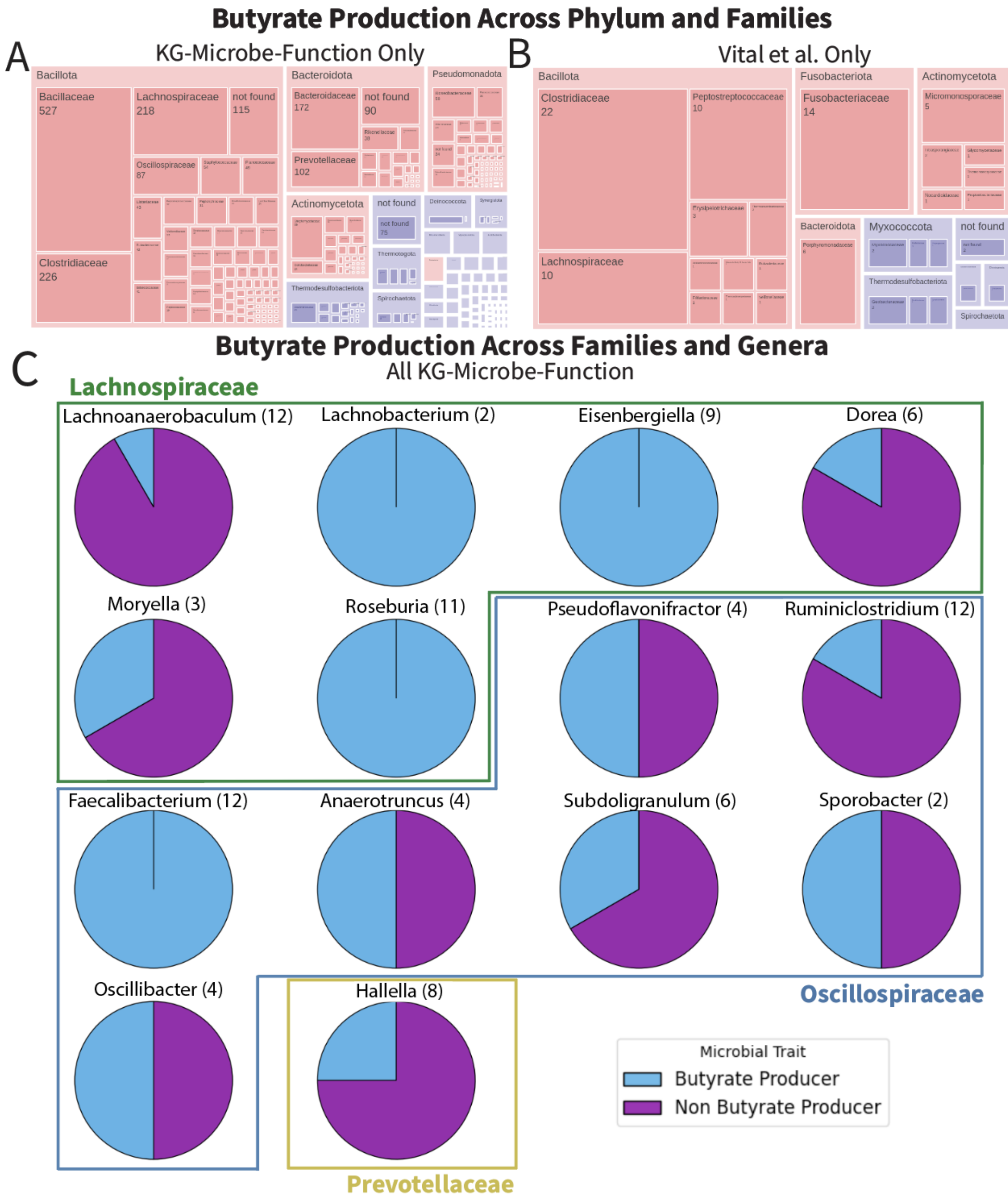

Figure 5. Treemaps of observed phyla and families among (A) annotated microbes in KG-Microbe-Function that are not in Vital et al. set and (B) microbes in the Vital et al. set that are not in KG-Microbe-Function. Red boxes signify the main phyla found to be present in the human gut, while purple boxes signify phyla not commonly found to be present in the human gut. (C) Proportion of butyrate producers among annotated microbes in KG-Microbe-Function, including families understood to be prominent butyrate producers in the gut.

3.2. Applications of KG-Microbe in the Context of Disease

We applied the representation of microbial taxonomy and function within KG-Microbe-Biomedical-Function in two disease contexts to demonstrate its usefulness in biomedicine. Examining the role of the microbiome in disease expands our understanding of how microbes can influence human health, either positively or negatively. With population-wide studies, microbial function is often predicted at a coarse taxonomic resolution. Through the representation of phylogenetic relationships in the KG, it is possible to explore microbial function at finer taxonomic resolution (the species or strain level), bringing us closer to targeted therapeutics such as probiotics to treat diseases. Butyrate production is a key metabolite for maintaining intestinal barrier function and reducing inflammation, and population studies have found significant reduction in intestinal microbes that produce butyrate in inflammatory bowel disease[30] and PD[35], where reduced intestinal barrier function is also observed. In PD, the aggregation of alpha-synuclein, often observed in the enteric nervous system, is heightened by increased inflammation from reduced butyrate production[35]. Population level results are useful to begin to characterize the difference in microbial communities in disease compared to health, but it is difficult to infer functional explanations for these differences due to the coarse taxonomic resolution of the results of 16S rRNA amplicon sequencing.

To explore the functional qualities of these correlative results, we used the taxonomic structure of KG-Microbe-Biomedical-Function to gather the ‘pan-genome’ of all strains below each taxon annotated as increasing or decreasing with disease. We found that a significantly higher number of butyrate producing microbes were associated with a decreased likelihood of both IBD and PD ($p = 1e\text{-}142$, $p = 2e\text{-}288$ respectively) (Figure 6). By traversing the KG in a semantically constrained way we were able to find representative finer-resolved species or strains for each taxon from the original source (labeled as “Original Source Representation” in Figure 6B) and therefore provide a clearer summary of the informative trait of butyrate production.

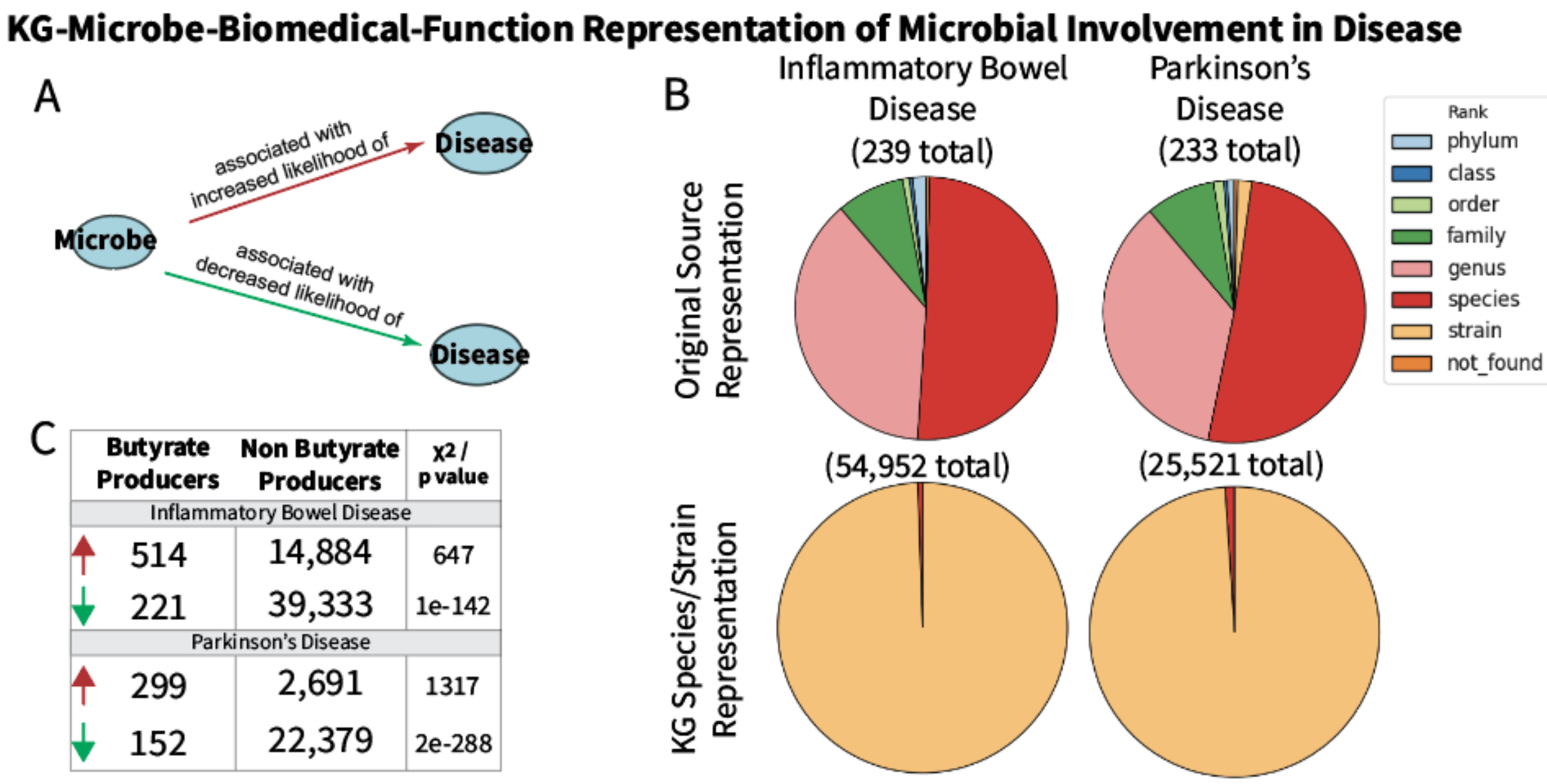

Figure 6. (A) Semantic representation of microbe-disease relationships in KG-Microbe-Biomedical-Function from Disbiome or Wallen et al. (B) The rank of disease annotations found directly in Disbiome or Wallen et al. (top), then transformed by finding all strain or species level children of each taxon (bottom) for both IBD and PD. (C) Using the species/strain representation of disease annotations, the proportion of butyrate producers along with the corresponding chi-squared test statistic comparing taxa annotated to increase (red arrow) or decrease (green arrow) likelihood of disease.

The phylogenetic comparison of butyrate production and disease association analyses are typical examples of general and popular research activities performed with microbial and microbiome data. We have demonstrated the ability of the KG to uncover microbial function at finer taxonomic resolutions than population level results. KG-Microbe enables analysis of such complex data relationships in a semantically- and taxonomically-aware manner while being supportive of the nuances and richness of microbial data.

3.3. Environmental Applications of KG-Microbe

Finally, we demonstrate the unique capability of the KG-Microbe framework in an environmental application and as a source of microbial traits for machine learning (ML)-based predictions. While over 10,000 bacterial and archaeal species exist in the human microbiome, the number of species living in Earth's environments is estimated to be at least in the millions, and

with a much larger diversity of environments and physical conditions, most of them inhospitable to humans[36,37]. Due to these differences, applying KG-Microbe to environmental applications utilizes different parts of the KG than when considering only the human microbiome. In addition, the biomedical examples focused on semantic representations and detailed assessments of paths for a specific outcome, whereas in this section we take an untargeted approach and consider all taxa which have an enzyme (EC) or reaction annotation (Rhea). These two node types can be considered equivalent representations in some cases (i.e., the enzyme can be semantically synonymous with the reaction it performs), though the precise outcomes of genome annotation can vary due to protein family models and sequence similarity cutoffs.

Using strains and species with edges to both temperature preference information and to genomic annotations of enzymatic activity (as EC nodes) and biochemical reactions (as Rhea nodes), we trained a gradient boosted decision tree classification model to predict four temperature classes: psychrophilic, mesophilic, thermophilic, and hyperthermophilic (see Methods). A semantic representation of genomic annotations was used to produce binary features for taxa (e.g. when the triples *"protein, biolink:participates_in, enzymatic activity"* and *"protein, biolink:derives_from, microbe"* exist, the corresponding *"enzymatic activity"* is included as a feature for that microbe). The multiclass model performed well with 90% and 61% precision and recall on withheld test data (macro avg. across four temperature classes). Interpreting the feature importance for hyperthermophilic and psychrophilic taxa (Figure 7), representing opposite extremes of temperature, revealed sets of EC and Rhea annotations with opposite importance (positive vs. negative), corresponding directly to enzyme and reaction presence/absence patterns.

We considered SHapley Additive exPlanations (SHAP) values, which measure the contribution of a given feature to the outcome of a predictive ML model[38]. The top important feature for predicting microbial temperature class preferences was the Catalase enzyme class (EC:1.11.1.6), with positive importance for psychrophilic and negative for hyperthermophilic

organisms. Oxygen solubility increases with lower temperature and psychrophile metabolism can generate additional oxygen[39], hence organisms in these environments could be subject to more oxidative stress. On the other hand hyperthermophiles often inhabit environments with low oxygen levels such as hot springs or deep sea vents and hydrogen peroxide is less stable at high temperatures[40]. Among the top 5 most important features for each class, hyperthermophiles were associated with positive features 4-alpha-glucanotransferase (EC:2.4.1.25), phosphotransferases with an alcohol group as an acceptor (EC:2.7.1.-), and protein acetyllysine N-acetyltransferase (EC:2.3.1.286); and psychrophiles with tRNA hydroxylases (RHEA:64224). In hyperthermophiles, presence of 4- alpha- glucanotransferase, phosphotransferases with an alcohol group as acceptor, and NAD^+ - dependent protein deacetylases reflects adaptations for robust carbohydrate utilization, energy efficiency such as ATP- saving glycolysis, and protein homeostasis at high temperatures, whereas the presence of tRNA hydroxylases in psychrophiles indicates enhanced tRNA modifications that maintain tRNA structural flexibility and translation efficiency at low temperatures[41–47]. The top unique positive feature for hyperthermophiles was adenosylmethionine decarboxylase (Rhea:15981), an enzyme involved in spermidine production, with spermidine known to increase DNA and protein stability[48]. The top unique feature for psychrophiles was RNA-directed DNA polymerase (EC:2.7.7.49), which could be explained by the fact that RNA molecules are highly heat labile and more stable at lower temperatures[49]. In summary, this machine learning application demonstrates that the harmonized knowledge in KG-Microbe can be used to predict broadly relevant biological insights such as temperature preference while providing specific explanations of important microbial features.

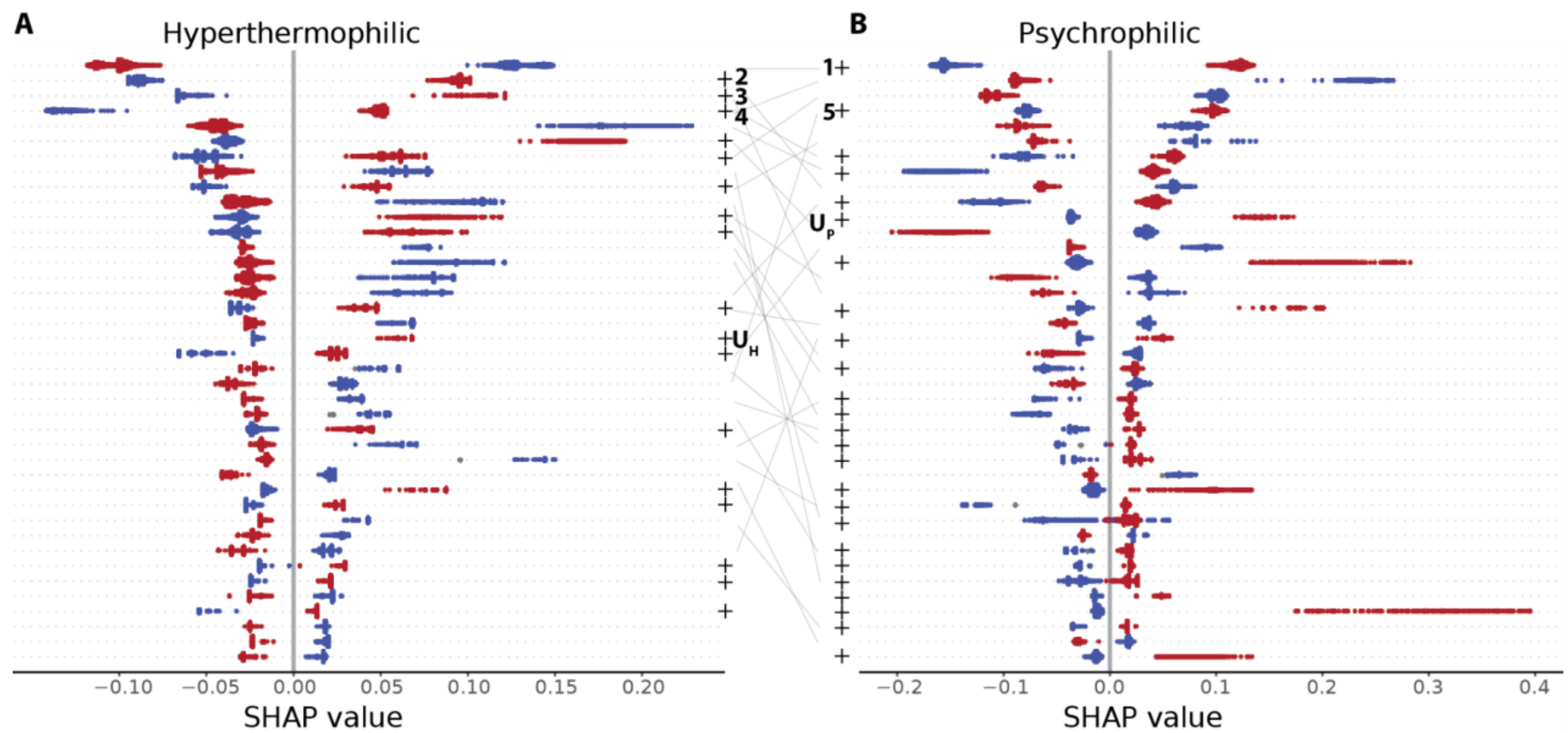

Figure 7. Feature importance for hyperthermophilic (A) and psychrophilic (B) taxa from a multiclass temperature classification model using EC enzyme and Rhea reaction UniProt annotations as features. The top 20 features are shown, sorted in descending order by mean absolute SHAP values based on the model training data. Red corresponds to feature presence and blue corresponds to feature absence. A '+' indicates that the presence of that feature is present and has a positive impact on the model while a '-' (not shown) corresponds to a negative impact and absence of that feature. Lines connect features with opposite ('+' vs '- ' or '-' vs '+') importance for the two classes, meaning flipped presence/absence in either direction. The top unique feature for hyperthermophiles (U_H) is adenosylmethionine decarboxylase and for psychrophiles (U_P) RNA-directed DNA polymerase. Across the two panels, the top-ranking features include: (1) catalase, (2) 4-alpha-glucanotransferase, (3) phosphotransferases with an alcohol group as acceptor, (4) NAD^+ protein deacetylase, (5) tRNA hydroxylase. SHAP values for all features can be found in the kg-microbe-paper github repository[50].

4. Discussion

The KG-Microbe framework supports the construction of diverse, harmonized, and broadly applicable microbial KGs that support biological discovery. To demonstrate utility and quality of the KGs we performed a series of analyses covering a wide range of applications from biomedical to environmental and beyond. Through evaluation by competency questions, we demonstrated that the harmonization and integration of multiple data sources resulted in an accurate model of microbial traits for applications in mechanistic inferences at varying levels of taxonomic resolution. Incorporating large sources of protein annotations in a semantically consistent structure allowed

straightforward inference over the KGs to make predictions about relevant microbial traits such as butyrate production. The applications of this microbial trait analysis in the context of IBD and PD exemplify how the KGs facilitate an understanding of microbial function at finer taxonomic resolutions. In the future, an evaluation of the catalog of microbial genome sequences by the HMP could be mapped to sequence identifiers or sequences as opposed to taxonomic names, providing additional validation and potentially increased taxonomic resolution. Furthermore, the HMP study was only based on the samples from healthy American participants, therefore greater diversity and coverage of a healthy microbiome community would be important to evaluate. These competencies can be extended to metabolic functions beyond butyrate production given the broad scope of the knowledge represented in the KG.

We have also demonstrated the utility of KG-Microbe-Function to support ML-based predictions by transforming the knowledge represented into binary features for use in inferences about microbial growth preferences and explanations of the functions involved. This example of collapsing the semantic representation of functional annotations can be extended to other microbial knowledge in the graph, such as organismal traits, or incorporating latent representations of graph structure such as graph embeddings. Embeddings or ML-based approaches to learn microbiome data have become increasingly successful, though cannot provide the same level of mechanistic detail as the semantic representations presented here and thus have limited explainablity[51]. These applications can be extended to other biological questions to further benefit from harmonized knowledge sources.

The four KG-Microbe framework KGs cover a broader scope of knowledge than previously captured in microbial KGs (Supplementary Table 1). The MetagenomicKG was recently published as a resource that effectively represents microbial sequences from the Genome Taxonomy Database (GTDB) with microbial functions described in the KEGG database, among other sources, in order to characterize pathogens in relation to human diseases, using data from KEGG, the Bacterial and Viral Bioinformatics Resource Center (BV-BRC), or MicroPhenoDB[9,16,52–55].

Because in MetagenomicKG microbes are not mapped to NCBI Taxonomy, the most comprehensive taxonomic classification resource, the traversal of phylogenetic relationships among taxa that is possible in all KG-Microbe graphs is much more limited with this resource[16]. Other microbial KGs represent a limited number of microbial relationships and contexts. MicrobiomeKG introduces a set of manually curated microbial associations from literature, while MGMLink integrates correlative results from the gutMGene database into a large biomedical KG[10,56]. MiKG4Md and Pre-/Probiotics Knowledge Graph similarly combine data from a series of manuscripts from a directed literature search to represent the role of the microbiome in mental health disorders[11,12]. The KG-Microbe biomedical graphs integrate a wider variety of microbe-disease information, incorporate organismal and genomic traits, and provide more knowledge of host physiology (human in this case) with functional annotations. The KG-Microbe framework fills an important need in building KGs relevant to a variety of microbial contexts that are rooted in broadly relevant knowledge of microbiology.

One limitation is the open-world nature of KGs in general—missing links are not negative evidence. Where high-confidence negatives exist (e.g., antibiotic sensitivity and resistance), we include them; expanding negative coverage is a priority for future releases. As with any KG, we are reliant on the knowledge in the ingested sources. UniProt is our main source of functional annotations and was chosen because of its partially reviewed proteome data, broad scope, and standardization of microbial function annotations. In comparison to resources such as the KEGG or MetaCyc, UniProt is freely available, supports frequent updates, and provides mappings to common primary resources to help with standardization, all of which ensures that the KG-Microbe Function graphs are up to date with recent genome annotation knowledge[22,53,57]. A large fraction of the predicted butyrate producing taxa from KG-Microbe-Function are based on UniProt annotations. Of the 3,600 taxa predicted to produce butyrate in the KG from the UniProt source, 2,859 are annotated with a "Probable butyrate kinase (BK)". UniProt includes both expert and community curation to inform protein annotations, however many of these annotations are not

experimentally validated. The inclusion of resources like BacDive alongside these functional annotations is important because BacDive organismal traits are derived from literature and laboratory experiments (see Methods) and hence can serve as an independent gold standard. Despite challenges in the accuracy and completeness of functional annotations, the provenance in our KG construction and the ability to compare across multiple data sources can increase confidence in assertions and predictions.

Our current strategy for resolving taxa involves both manual and automated approaches. While large language model (LLM)-based mapping strategies are beginning to be used for extracting information from literature and databases for KG construction, its applications for microbes are more challenging. Microbial naming can be inconsistent across sources due to challenges in sequence resolution and classification. Conflicts and ambiguities arise among publications and databases based on naming conventions of microbes, complicating automated strategies. In this paper, a semi-automated approach was used to resolve taxa for the few cases where taxa were not already mapped to NCBI Taxonomy. This was due to the relatively small numbers of taxa to resolve, and the manual adjustments were critical to maximize coverage.

A future direction of this KG framework is to provide a sequence-integration layer for taxa rather than names and identifiers mapped to NCBI Taxonomy. By introducing taxa based on sequence integration of whole genomes or by sequence accession identifiers, an additional layer of confidence and provenance is enabled, and this also supports integration of novel taxa not represented in existing taxonomies by linking them to the closest neighbor or appropriate taxonomy rank. Because BacDive provides sequence provenance for each strain-level record, 16S or whole genome sequences can be found from the BacDive source using the corresponding NCBI Taxonomy or BacDive IDs from the KG.

The KGs are also limited by the lack of reaction directionality for genome annotations due to the bi-directional representation of reactions in UniProt. We introduced reaction directions based on the eQuilibrator API, which can be broadly applicable to chemical reaction knowledge

represented in this way. A future direction would be to add this to the KG semantic representation of biochemical reactions with additional assumptions (e.g. standard growth conditions). Further, there is potential to incorporate more evidence-based models of microbial metabolism such as Genome-Scale Metabolic Networks (GSMNs). Resources such as the Biochemical, Genetic and Genomic knowledge base (BiGG) or MetaNetX could provide these on a larger scale[58,59]. There are also knowledge bases such as AGORA2, which consolidates GSMNs of gut microbiome-relevant organisms, that can be used in predictive models of microbial biochemical interactions to provide more targeted analyses of specific taxa[60,61].

The biomedical KGs include microbe-disease correlations from Disbiome, which provides a range of microbe-relevant diseases and correlations from literature. The broad scope of disease from this resource supports KG analyses such as understanding butyrate production in the context of inflammatory diseases (e.g., IBD and PD). However, as shown in our disease analysis, Disbiome annotations are at a coarse taxonomic resolution. It would be useful to introduce metagenomics studies of microbes in a disease context to expand the function insights that can be gained from KG-Microbe-Biomedical-Function. Several other structured disease databases exist that could be introduced to the biomedical KG to expand the possibilities of inference in other disease contexts[5].

The functional KGs constructed with this framework are compliant with the Biolink model, with the exception that they all include potentially overlapping node types such as EC, Rhea, and GO, which all represent biochemical activity in different ways. This ensures that protein or microbe annotations from all sources are fully represented in the graph (UniProt, BacDive). Not all potentially equivalent annotations are found for each protein, even in a standardized resource like UniProt due to nuances of sequence annotation. Future versions of the KGs might collapse overlapping nodes based on equivalency analysis, for example combining EC and Rhea terms as synonyms which pertain to identical biochemical reactions, with confirmed equivalence in sequence annotations, to ensure uniqueness in all node types and support more straightforward

inference. Combining EC and Rhea nodes can also reduce the number of content edges and provide more straightforward paths describing protein function. However, this design prioritizes preserving all information over minimizing edges, thereby more accurately capturing the knowledge required for inference. These node normalization tasks are important considerations for the modeling choices of each KG built from this framework. Our competency questions demonstrate the importance of fully representing sequence annotations. Furthermore, functional annotation methods for microbes are still being expanded and refined.

We present a novel framework for constructing modular KGs that are relevant to a variety of applications within the microbiome and microbial sciences. Through targeted use cases for the KGs, we demonstrate that KG-Microbe-Core provides a base for inference and learning about organismal traits of microbes, KG-Microbe-Function enables the identification of important microbial functions, and KG-Microbe-Biomedical-Function broadens our understanding the host-associated microbiome. With the standardization of both experimental observations and functional predictions according to the Biolink Model, all KG-Microbe KGs demonstrate the ability to draw from the many sources of knowledge available in an accurate way that increases their collective utility. The large-scale integration of functional and trait information from literature, structured databases, and experimental observations in the KG-Microbe framework offers unrivaled standardization and coverage of microbial traits and biology.

5. Potential Implications

The architecture of this framework supports a variety of applications for the resulting KGs, from understanding specific mechanisms of the host microbiome in disease to providing growth preference predictions for microbes. The KG-Microbe framework paves the way for modular KG development, where customizable KGs can be constructed based on standardized transform and modeling patterns for a wide range of data sources. We present the core KG as well as three other custom KGs in this work, though the underlying host or datasets modeled can be adjusted

according to the underlying biomedical, biological, or ecological question. This framework and ecosystem of KGs enable a wide range of applications, from generating hypotheses about microbial involvement in disease at the species or strain level—critical for biomedical applications such as biomarker development and targeted therapeutics—to leveraging ML methods to utilize the diverse biological information for predictive modeling.

6. Methods

In this section we first describe the architecture of the KG-Microbe framework used to construct the four KGs described, as well as how this framework can be generalized to build customized KGs. The modeling details for representing all node types according to knowledge from each source in all KGs are described in Section 2.1.1-2.1.5 (Table 1, Supplementary Figure 2). The source-specific preprocessing and analysis procedures are provided in the Supplementary Methods (1.1–1.11). Finally, we discuss evaluation methods.

Table 1. Description of all resources included in the KG-Microbe framework.

**Node Type(s) Represented**	**Standardized Mappings**
Organism	NCBI Taxonomy (NCBITaxon)[16] , Custom "*strain*" (Bacterial Diversity Metadatabase (BacDive)[17])
Chemical	Chemical Entities of Biological Interest (ChEBI)[76], CAS Registry (CAS-RN)[77], PubChem[78], Kyoto Encyclopedia of Genes and Genomes (KEGG)
Antibiotic	Chemical Entities of Biological Interest (ChEBI)[76]
Isolation Source	Chemical Entities of Biological Interest (ChEBI)[76], Environment Ontology (ENVO)[68], Plant Ontology (PO)[69], Phenotype and Trait Ontology (PATO)[72], Uberon Ontology (UBERON), Custom "*isolation_source*" (Bacterial Diversity Metadatabase (BacDive)[17] and Madin et al.[19])
Enzymatic Activity	Enzyme Commission (EC)[79]
Optimal pH	Custom "*pH_opt*" (BactoTraits[18])
pH Range	Custom "*pH_range*" (BactoTraits[18])
pH Spread	Custom "*pH_delta*" (BactoTraits[18])
Temperature Category	Custom "*temperature*" (BactoTraits[18])
Optimal Temperature	Custom "*temp_opt*" (BactoTraits[18])
Temperature Range	Custom "*temp_range*" (BactoTraits[18])
Temperature Spread	Custom "*temp_delta*" (BactoTraits[18])
Optimal NaCl Concentration	Custom "*NaCl_opt*" (BactoTraits[18])
NaCl Concentration Range	Custom "*NaCl_range*" (BactoTraits[18])

NaCl Concentration Spread	Custom "*NaCl_delta*" (BactoTraits[18])
Oxygen Category	Custom "*oxygen*" (Bacterial Diversity Metadatabase (BacDive)[17], BactoTraits[14], Madin et al.[19])
GC Content	Custom "*gc*" (BactoTraits[18])
Motility	Custom "*motility*" (Bacterial Diversity Metadatabase (BacDive)[17], BactoTraits[18])
Trophic Type	Custom "*trophic_type*" (Bacterial Diversity Metadatabase (BacDive)[17], BactoTraits[18], Madin et al.[19])
Cell Width	Custom "*cell_width*" (BactoTraits[18])
Cell Length	Custom "*cell_length*" (BactoTraits[18])
Cell Shape	Custom "*cell_shape*" (Bacterial Diversity Metadatabase (BacDive)[17], BactoTraits, Madin et al.[19])
Sporulation	Custom "*sporulation*" (Bacterial Diversity Metadatabase (BacDive)[17], BactoTraits)
Pathway	Custom *"pathways"* (Madin et al.[19])
Biosafety Level	Custom "*BSL*" (Bacterial Diversity Metadatabase (BacDive)[17]
Medium	Custom *"medium"* (Bacterial Diversity Metadatabase (BacDive)[17], MediaDive[21])
Solution	Custom *"solution"* (MediaDive[21])
Ingredient	Chemical Entities of Biological Interest (ChEBI)[76], CAS Registry (CAS-RN)[77], PubChem[78], Kyoto Encyclopedia of Genes and Genomes (KEGG)[53], Custom *"ingredient"* (MediaDive[21])
Assay	Custom "*assay*" (Bacterial Diversity Metadatabase (BacDive)[17])
Protein	Uniprot Knowledgebase (UniProt)[22]
Biological process	Gene Ontology (GO)[80]
Molecular function	Gene Ontology (GO)[80]
Cellular Component	Gene Ontology (GO)[80]
Disease	Mondo Disease Ontology (MONDO)[24], Human Phenotype Ontology (HPO)[81], Gene Ontology (GO)[80]
Human Phenotype	Human Phenotype Ontology (HPO)[81]
Metabolic pathway	UniPathways (UPA)[23]
Human Gene	HUGO Gene Nomenclature Committee (HGNC)[82]
Biochemical Reaction	Rhea[83], *"UPA:UCR"* (Unipathways Ontology (UPA))
Metabolic Pathway	*"UPA:UPA"* (Unipathways Ontology (UPA))

6.1. KG-Microbe Architecture

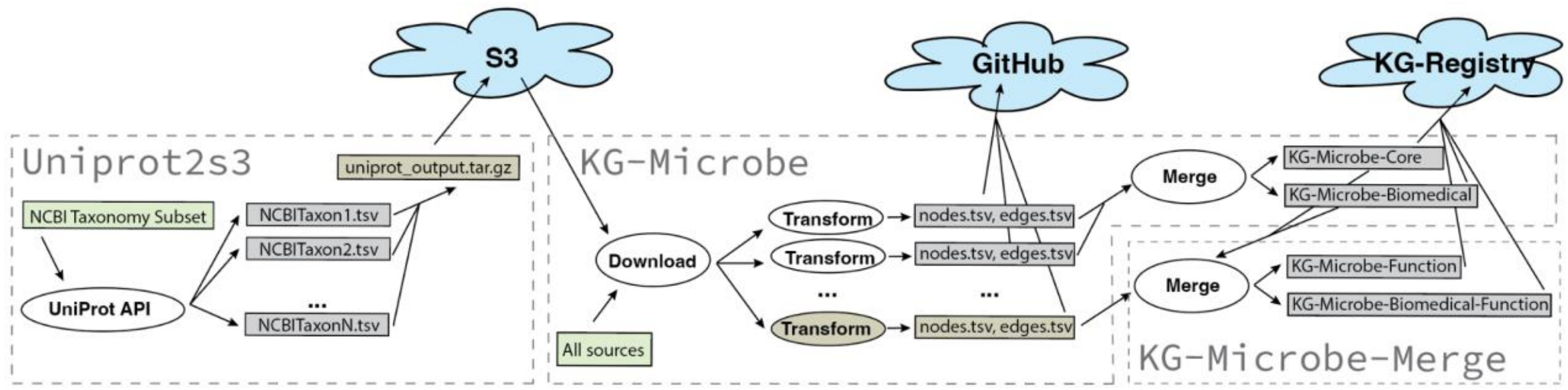

Figure 8. Architecture of the build process for KGs built from the KG-Microbe framework. First UniProt is queried for all organisms of interest which stores the raw responses in a TSV file per organism. These are compressed into a single data product and stored in S3 (yellow boxes). Next all input sources are downloaded from S3, the Open Biological and Biomedical Ontology (OBO) Foundry, or cached API downloads in Google Drive as input to the unique transform code for each source. These transforms are then released as compressed files in GitHub or S3 for large files. Last, the transform components are merged according to the desired graph and content. Final products are stored in KG-Registry.

The download, transform, and merge steps for constructing KGs in the KG-Microbe framework, build upon the KG-Hub construction process and involve three coordinated code repositories: Uniprot2s3, KG-Microbe, and KG-Microbe-Merge (Figure 8)[15]. The uniprot2s3 repository is used to download information using the UniProt API (see the “UniProt” section below)[62]. The underlying infrastructure that supports the construction is KG-Hub and the KGX tool (version 2.4.2), which provides a template repository and tooling for KG construction[14]. During the transform step, content from all sources is normalized and modeled according to the “KG-Microbe Construction and Modeling” section below by the kg-microbe repository, and redundant edges and nodes are accounted for in cases where overlapping information exists in multiple sources[63]. The resulting transform modules in KGX format are then published in a GitHub release to provide additional data, provenance, and to support interoperability with other sources and KGs. Finally, sources are merged into distinct KG resources. KGX was used to merge KG-Microbe-Core and KG-Microbe-Biomedical in the kg-microbe repository. Due to the size of the microbial UniProt transform, we implemented a merge process using DuckDB to merge the UniProt edges in KG-Microbe-Function and KG-Microbe-Biomedical-Function with other sources in a memory efficient way[64]. Timing metrics for this build process can be found in Supplementary Table 2.

Beyond the four KGs presented, KG-Microbe is a generalizable framework for microbial KG construction. The source dataset transformations included in the current release demonstrate the framework's flexibility through their diverse data structures (such as OWL, JSON, and CSV), serving as templates for integrating additional sources. Standardized ontology formats can easily be introduced using the framework's KG-Hub infrastructure and KGX tooling, which enables merging with other KGs within compatible ecosystems. New data sources can be integrated by following the demonstrated patterns: API-based ingestion (as shown with UniProt via uniprot2s3), file-based transformation (as implemented for BacDive and other sources), and memory-efficient merging strategies (as developed for large-scale UniProt data using DuckDB). We have applied this generalizability in the construction of KG-Microbe-Biomedical and KG-Microbe-Biomedical-Function, for which we ingested two additional ontologies, the Parkinson's disease microbiome data from Wallen et al., and the Disbiome database. These KGs therefore serve as an exemplar for domain-specific extensions.

6.2. Evaluation Methods

6.2.1. Butyrate Analysis

We used a metagenomic analysis by Vital et al. of 3,184 sequenced bacterial genomes from the publicly available Integrated Microbial Genome database, which documented 225 predicted butyrate producing bacteria at the species or strain level. 216 of these taxa were able to be mapped to a NCBI Taxonomy ID using a pattern-based fuzzy string matching. First a direct string match was attempted. Next, brackets were included around the first term, assumed to be a genus name, and the match was attempted again. Finally *“sp.”* was included in the name and the match was attempted again. Any microbes that were not able to be mapped to an NCBI Taxonomy term were manually curated when possible, leaving only 3 *“not found”* taxa from the Vital et al. set.

We compared these taxa to those represented in KG-Microbe from various sources. Butyrate production is represented in the graph from BacDive or as functional annotations from UniProt. In BacDive, microbes are cited as being capable of producing butyrate from literature curation or in house experiments. One semantic representation involving UniProt data was microbes with proteins involved in biochemical reactions (Rhea nodes) that have butyrate as a product. Because reaction annotations in UniProt are undirected, we applied the eQuilibrator methodology to predict the direction of a given reaction, which uses group contribution to estimate free energy of formation ($\Delta fG°$)[84]. Overall, this approach contributed 3,176 total taxa to the set of predicted butyrate producers from the KG. Another semantic representation involving UniProt data showed which microbes were capable of defined sets of EC numbers, allowing for one missing annotated reaction. These EC sets were identical to those used in the Vital et al. metagenomic analysis to represent four butyrate producing pathways (the acetyl-CoA, glutarate, 4-aminobutyrate, and lysine pathways, Figure 3A).

We searched for all strains and species for relevant families using DuckDB over KG-Microbe-Biomedical-Function. We use the *"biolink:subclass_of"* edge to find all children of each given taxa. Because there exists taxa without a rank assignment in NCBI Taxonomy, we excluded these and any children of the taxa without rank from analysis. All species and strain level butyrate producers in KG-Microbe-Function were found, and their corresponding family and phylum assignment was identified in order to constrain the list by only those phyla relevant to the human gut[2]. We then evaluated key families known for butyrate production in the gut microbiome: Ruminococcaceae, Lachnospiraceae, Bacteroidaceae, Prevotellaceae, Clostridiaceae, Oscillospiraceae, and Eubacteriaceae[34]. Of that list, we evaluated the proportion of total taxa in the family, according to knowledge represented in the KG from NCBI Taxonomy, to butyrate producers (Figure 4C). All competency queries can be found in the kg-microbe-paper github repository[50].

6.3. Disease Analysis

Microbes with a disease relationship were found in KG-Microbe-Biomedical-Function based on edge and node type. The edges "*biolink:associated_with_decreased_likelihood_of*" and "*biolink:associated_with_increased_likelihood_of*" were used for the analysis in both IBD and PD. To identify microbes involved in IBD, we included the diseases Crohn's disease and Ulcerative colitis in the search, since they are children of the IBD disease class according to MONDO. The resulting microbe:disease pairs were sourced from Disbiome and Wallen et al. Microbes with an inconsistent direction of their disease relationship across studies were excluded. We then traversed KG-Microbe-Biomedical-Function, similarly to the methods described in the "Butyrate Annotation" section to find all strains or species of a query taxon, at any rank, at a finer taxonomic resolution. When no strains existed in NCBI Taxonomy beneath the given taxon, we evaluated species. From the collection of protein annotations to these taxa, we then characterized them as being butyrate producers or not based on the KG organismal and genomic trait representation in Figure 3A. We then compared the fraction of taxa annotated to produce butyrate out of all representative strains (or species) between those that increase or decrease likelihood of the disease.

6.4. Temperature Classification Model

A catboost multiclass classification model was trained using KG-Microbe EC and Rhea UniProt binary annotations. Rows and columns with less than two observations were removed. All four temperature classes were used, all with at least 40 training examples: psychrophilic, mesophilic, thermophilic, and hyperthermophilic. Rows and columns with less than two observations were removed. 25 EC features were removed because of identity with another EC feature. Data was split with stratification as 70/20/10% for train/validation/test, with hyperthermophiles having the fewest test data examples with (N= 8). Training data consisted of 5,628 taxa and 6,177 EC and Rhea features. The catboost parameters were 200,000 iterations,

depth of 4, learning rate of 0.05, L2 regularization term of 4, bagging temperature of 1, random strength of 6, a MultiClass loss function, random seed of 42, verbosity of 100, early stopping rounds of 50, and use best model set to True. CatBoost model SHAP values were computed using the TreeExplainer shap_values() method from the SHAP python package. SHAP values were computed on the training set for each class. SHAP summary plots show class-specific SHAP values and were generated using the TreeExplainer method from the SHAP package (version 0.39.0) with shap.summary_plot(), displaying the top 40 most important features per temperature class. SHAP values for all features for each temperature class are available in the analysis repository[50].

7. Availability of Source Code and Requirements

Project name: KG-Microbe

Project homepage: https://github.com/Knowledge-Graph-Hub/kg-microbe

Operating system(s): Platform independent

Programming language: Python 3.10

Other requirements:

License: The KG-Microbe codebase is licensed under an BSD-3-Clause license and the associated data with a CC0 1.0 license.

Restrictions to use by non-academics: Both the CC0 1.0 license (dataset) and the BSD-3-Clause license (codebase) allow for both commercial and non-commercial use.

8. Data Availability

All metadata for the KG resources are available in KG-Registry[15]. In addition, KG-Microbe-Core and KG-Microbe-Biomedical are available in KG-Hub. The code for the framework to create each transformed module for KG-Microbe graphs and to merge the non-function graphs can be accessed online[63]. The code to generate raw files for incorporating UniProt annotations can be

accessed online[62]. The code to merge transformed modules for the function graphs in the graph can be accessed online[64]. The code for all analyses can be accessed online[50].

9. List of abbreviations

KG: knowledge graph; EC: enzyme commission; IBD: inflammatory bowel disease; PD: Parkinson's disease; ID: identifier; API: application programming interface; API assay: Analytical Profile Index assay; AI: artificial intelligence; ML: machine learning

10. Declarations

Not applicable

11. Consent for publication

Not applicable

12. Competing interests

The authors declare no competing interests.

13. Funding

This work was supported in part by the Director, Office of Science, Office of Basic Energy Sciences, of the US Department of Energy, Contract No. DE-AC0205CH11231. Research reported in this publication was also supported by the National Library of Medicine (NLM) of the National Institutes of Health (NIH) under award number T15LM009451. This research was also funded by grant number R01LM013400 and OT2TR003422.

14. Author Contributions

Conceptualization: B.E.S, M.P.J, Methodology: B.E.S, M.P.J, J.R Software: B.E.S, M.P.J, H.H., H.C., J.R., Investigation: B.E.S, M.P.J, Resources: B.E.S, M.P.J, H.H., H.C., J.R., C.J.M., Formal Analysis: B.E.S, M.P.J, Supervision: M.P.J, L.E.H., C.A.L., C.J.M., Funding Acquisition: B.E.S, M.P.J, Validation: B.E.S., M.P.J, Investigation: B.E.S., M.P.J., Visualization: B.E.S.,

M.P.J., Writing - original draft: B.E.S, M.P.J., Writing - review & editing: B.E.S, M.P.J., H.C., J.R., C.J.M., T.K., L.E.H., C.A.L.

15. References

1. Kim, N. *et al.* Genome-resolved metagenomics: a game changer for microbiome medicine. *Exp Mol Med* **56**, 1501–1512 (2024).
2. Hou, K. *et al.* Microbiota in health and diseases. *Sig Transduct Target Ther* **7**, 135 (2022).
3. Langille, M. G. I. *et al.* Predictive functional profiling of microbial communities using 16S rRNA marker gene sequences. *Nat Biotechnol* **31**, 814–821 (2013).
4. Mann, E. R., Lam, Y. K. & Uhlig, H. H. Short-chain fatty acids: linking diet, the microbiome and immunity. *Nat Rev Immunol* **24**, 577–595 (2024).
5. Santangelo, B. E. *et al.* Integrating biological knowledge for mechanistic inference in the host-associated microbiome. *Front. Microbiol.* **15**, 1351678 (2024).
6. Youn, J., Rai, N. & Tagkopoulos, I. Knowledge integration and decision support for accelerated discovery of antibiotic resistance genes. *Nat Commun* **13**, 2360 (2022).
7. Putman, T. E. *et al.* The Monarch Initiative in 2024: an analytic platform integrating phenotypes, genes and diseases across species. *Nucleic Acids Research* **52**, D938–D949 (2024).
8. Callahan, T. J. *et al.* An Open-Source Knowledge Graph Ecosystem for the Life Sciences. https://doi.org/10.48550/ARXIV.2307.05727 (2023) doi:10.48550/ARXIV.2307.05727.
9. Ma, C., Liu, S. & Koslicki, D. MetagenomicKG: a knowledge graph for metagenomic applications. Preprint at https://doi.org/10.1101/2024.03.14.585056 (2024).

10. Goetz, S. L., Glen, A. K. & Glusman, G. MicrobiomeKG: Bridging Microbiome Research and Host Health Through Knowledge Graphs. Preprint at https://doi.org/10.1101/2024.10.10.617697 (2024).
11. Liu, T., Lan, G., Feenstra, K. A., Huang, Z. & Heringa, J. Towards a knowledge graph for pre-/probiotics and microbiota–gut–brain axis diseases. *Sci Rep* **12**, 18977 (2022).
12. Liu, T. *et al.* Predicting the relationships between gut microbiota and mental disorders with knowledge graphs. *Health Inf Sci Syst* **9**, 3 (2021).
13. Unni, D. R. *et al.* Biolink Model: A universal schema for knowledge graphs in clinical, biomedical, and translational science. *Clinical Translational Sci* **15**, 1848–1855 (2022).
14. Caufield, J. H. *et al.* KG-Hub—building and exchanging biological knowledge graphs. *Bioinformatics* **39**, btad418 (2023).
15. Harry Caufield, actions-user, Michael Gargano, DnlRKorn & Justin Reese. Knowledge-Graph-Hub/kg-registry: 0.0.1. Zenodo https://doi.org/10.5281/ZENODO.15026976 (2025).
16. Schoch, C. L. *et al.* NCBI Taxonomy: a comprehensive update on curation, resources and tools. *Database (Oxford)* **2020**, baaa062 (2020).
17. Söhngen, C., Bunk, B., Podstawka, A., Gleim, D. & Overmann, J. BacDive—the Bacterial Diversity Metadatabase. *Nucl. Acids Res.* **42**, D592–D599 (2014).
18. Cébron, A. *et al.* BactoTraits – A functional trait database to evaluate how natural and man-induced changes influence the assembly of bacterial communities. *Ecological Indicators* **130**, 108047 (2021).
19. Madin, J. S. *et al.* A synthesis of bacterial and archaeal phenotypic trait data. *Sci Data* **7**, 170 (2020).
20. Reimer, L. C. *et al.* Bac *Dive* in 2022: the knowledge base for standardized bacterial and archaeal data. *Nucleic Acids Research* **50**, D741–D746 (2022).
21. Koblitz, J. *et al.* Media *Dive* : the expert-curated cultivation media database. *Nucleic Acids Research* **51**, D1531–D1538 (2023).

22. The UniProt Consortium *et al.* UniProt: the Universal Protein Knowledgebase in 2023. *Nucleic Acids Research* **51**, D523–D531 (2023).

23. Morgat, A. *et al.* UniPathway: a resource for the exploration and annotation of metabolic pathways. *Nucleic Acids Research* **40**, D761–D769 (2012).

24. Vasilevsky, N. A. *et al. Mondo: Unifying Diseases for the World, by the World*. http://medrxiv.org/lookup/doi/10.1101/2022.04.13.22273750 (2022) doi:10.1101/2022.04.13.22273750.

25. Janssens, Y. *et al.* Disbiome database: linking the microbiome to disease. *BMC Microbiol* **18**, 50 (2018).

26. Wallen, Z. D. *et al.* Metagenomics of Parkinson's disease implicates the gut microbiome in multiple disease mechanisms. *Nat Commun* **13**, 6958 (2022).

27. Davis, A. P. *et al.* Comparative Toxicogenomics Database (CTD): update 2021. *Nucleic Acids Research* **49**, D1138–D1143 (2021).

28. The Human Microbiome Project Consortium. Structure, function and diversity of the healthy human microbiome. *Nature* **486**, 207–214 (2012).

29. Peng, L., Li, Z.-R., Green, R. S., Holzmanr, I. R. & Lin, J. Butyrate Enhances the Intestinal Barrier by Facilitating Tight Junction Assembly via Activation of AMP-Activated Protein Kinase in Caco-2 Cell Monolayers. *The Journal of Nutrition* **139**, 1619–1625 (2009).

30. Recharla, N., Geesala, R. & Shi, X.-Z. Gut Microbial Metabolite Butyrate and Its Therapeutic Role in Inflammatory Bowel Disease: A Literature Review. *Nutrients* **15**, 2275 (2023).

31. Beber, M. E. *et al.* eQuilibrator 3.0: a database solution for thermodynamic constant estimation. *Nucleic Acids Research* **50**, D603–D609 (2022).

32. Vital, M., Howe, A. C. & Tiedje, J. M. Revealing the Bacterial Butyrate Synthesis Pathways by Analyzing (Meta)genomic Data. *mBio* **5**, e00889-14 (2014).

33. Markowitz, V. M. *et al.* IMG: the integrated microbial genomes database and comparative analysis system. *Nucleic Acids Research* **40**, D115–D122 (2012).

34. Singh, V. *et al.* Butyrate producers, "The Sentinel of Gut": Their intestinal significance with and beyond butyrate, and prospective use as microbial therapeutics. *Front. Microbiol.* **13**, 1103836 (2023).

35. Elford, J. D., Becht, N., Garssen, J., Kraneveld, A. D. & Perez-Pardo, P. Buty and the beast: the complex role of butyrate in Parkinson's disease. *Front. Pharmacol.* **15**, 1388401 (2024).

36. Rup, L. The Human Microbiome Project. *Indian J Microbiol* **52**, 315–315 (2012).

37. Louca, S., Mazel, F., Doebeli, M. & Parfrey, L. W. A census-based estimate of Earth's bacterial and archaeal diversity. *PLoS Biol* **17**, e3000106 (2019).

38. Lundberg, S. & Lee, S.-I. A Unified Approach to Interpreting Model Predictions. Preprint at https://doi.org/10.48550/ARXIV.1705.07874 (2017).

39. Chattopadhyay, M. K. *et al.* Increase in Oxidative Stress at Low Temperature in an Antarctic Bacterium. *Curr Microbiol* **62**, 544–546 (2011).

40. Ladenstein, R. & Antranikian, G. Proteins from hyperthermophiles: Stability and enzymatic catalysis close to the boiling point of water. in *Biotechnology of Extremophiles* (ed. Antranikian, G.) vol. 61 37–85 (Springer Berlin Heidelberg, Berlin, Heidelberg, 1998).

41. Jeon, B. *et al.* 4- α- Glucanotransferase from the Hyperthermophilic Archaeon *Thermococcus Litoralis*: Enzyme Purification and Characterization, and Gene Cloning, Sequencing and Expression in *Escherichia Coli*. *European Journal of Biochemistry* **248**, 171–178 (1997).

42. Akerboom, A. P. Functional analysis of thermostable proteins involved in carbohydrate metabolism. (Wageningen University, 2007). doi:10.18174/31866.

43. Verhees, C. H. *et al.* ADP-Dependent Phosphofructokinases in Mesophilic and Thermophilic Methanogenic Archaea. *J Bacteriol* **183**, 7145–7153 (2001).

44. Tuininga, J. E. *et al.* Molecular and Biochemical Characterization of the ADP-dependent Phosphofructokinase from the Hyperthermophilic Archaeon Pyrococcus furiosus. *Journal of Biological Chemistry* **274**, 21023–21028 (1999).
45. Hachisuka, S., Sato, T. & Atomi, H. Metabolism Dealing with Thermal Degradation of NAD^+ in the Hyperthermophilic Archaeon Thermococcus kodakarensis. *J Bacteriol* **199**, (2017).
46. Cao, J., Wang, T., Wang, Q., Zheng, X. & Huang, L. Functional Insights Into Protein Acetylation in the Hyperthermophilic Archaeon Sulfolobus islandicus*. *Molecular & Cellular Proteomics* **18**, 1572–1587 (2019).
47. Edwards, A. M., Addo, M. A. & Dos Santos, P. C. Extracurricular Functions of tRNA Modifications in Microorganisms. *Genes* **11**, 907 (2020).
48. Li, B., Kurihara, S., Kim, S. H., Liang, J. & Michael, A. J. A polyamine-independent role for *S* -adenosylmethionine decarboxylase. *Biochemical Journal* **476**, 2579–2594 (2019).
49. Becskei, A. & Rahaman, S. The life and death of RNA across temperatures. *Computational and Structural Biotechnology Journal* **20**, 4325–4336 (2022).
50. Santangelo, B. E. bsantan/kg-microbe-paper. (2025).
51. Pope, Q., Varma, R., Tataru, C., David, M. M. & Fern, X. Learning a deep language model for microbiomes: The power of large scale unlabeled microbiome data. *PLoS Comput Biol* **21**, e1011353 (2025).
52. Parks, D. H. *et al.* GTDB: an ongoing census of bacterial and archaeal diversity through a phylogenetically consistent, rank normalized and complete genome-based taxonomy. *Nucleic Acids Research* **50**, D785–D794 (2022).
53. Kanehisa, M., Furumichi, M., Sato, Y., Kawashima, M. & Ishiguro-Watanabe, M. KEGG for taxonomy-based analysis of pathways and genomes. *Nucleic Acids Research* **51**, D587–D592 (2023).

54. Yao, G. *et al.* MicroPhenoDB Associates Metagenomic Data with Pathogenic Microbes, Microbial Core Genes, and Human Disease Phenotypes. *Genomics, Proteomics & Bioinformatics* **18**, 760–772 (2020).
55. Olson, R. D. *et al.* Introducing the Bacterial and Viral Bioinformatics Resource Center (BV-BRC): a resource combining PATRIC, IRD and ViPR. *Nucleic Acids Research* **51**, D678–D689 (2023).
56. Santangelo, B., Bada, M., Hunter, L. & Lozupone, C. *Hypothesizing Mechanistic Links between Microbes and Disease Using Knowledge Graphs*. http://biorxiv.org/lookup/doi/10.1101/2023.12.01.569645 (2023) doi:10.1101/2023.12.01.569645.
57. Caspi, R. *et al.* The MetaCyc database of metabolic pathways and enzymes - a 2019 update. *Nucleic Acids Research* **48**, D445–D453 (2020).
58. Norsigian, C. J. *et al.* BiGG Models 2020: multi-strain genome-scale models and expansion across the phylogenetic tree. *Nucleic Acids Research* gkz1054 (2019) doi:10.1093/nar/gkz1054.
59. Moretti, S., Tran, V. D. T., Mehl, F., Ibberson, M. & Pagni, M. MetaNetX/MNXref: unified namespace for metabolites and biochemical reactions in the context of metabolic models. *Nucleic Acids Research* **49**, D570–D574 (2021).
60. Heinken, A. *et al.* Genome-scale metabolic reconstruction of 7,302 human microorganisms for personalized medicine. *Nat Biotechnol* 1–12 (2023) doi:10.1038/s41587-022-01628-0.
61. Diener, C., Gibbons, S. M. & Resendis-Antonio, O. MICOM: Metagenome-Scale Modeling To Infer Metabolic Interactions in the Gut Microbiota. *mSystems* **5**, e00606-19 (2020).
62. Santangelo, B. E. & Hegde, H. Knowledge-Graph-Hub/uniprot2s3. (2025).

63. Santangelo, B. E., Joachimiak, M. P., Caufield, J. H., Mungall, C. J. & Hegde, H. Knowledge-Graph-Hub/kg-microbe. (2025).

64. Santangelo, B. E., Hegde, H. & Joachimiak, M. P. Knowledge-Graph-Hub / kg-microbe-merge. (2025).

65. Weinstein, M. P. & Lewis, J. S. The Clinical and Laboratory Standards Institute Subcommittee on Antimicrobial Susceptibility Testing: Background, Organization, Functions, and Processes. *J Clin Microbiol* **58**, e01864-19 (2020).

66. Madin, J. S. & Nielsen, D. A. bacteria-archaea-traits.

67. Degtyarenko, K. *et al.* ChEBI: a database and ontology for chemical entities of biological interest. *Nucleic Acids Research* **36**, D344–D350 (2007).

68. Buttigieg, P. *et al.* The environment ontology: contextualising biological and biomedical entities. *J Biomed Sem* **4**, 43 (2013).

69. Cooper, L. & Jaiswal, P. The Plant Ontology: A Tool for Plant Genomics. in *Plant Bioinformatics* (ed. Edwards, D.) vol. 1374 89–114 (Springer New York, New York, NY, 2016).

70. Mungall, C. J., Torniai, C., Gkoutos, G. V., Lewis, S. E. & Haendel, M. A. Uberon, an integrative multi-species anatomy ontology. *Genome Biol* **13**, R5 (2012).

71. Dooley, D. M. *et al.* FoodOn: a harmonized food ontology to increase global food traceability, quality control and data integration. *npj Sci Food* **2**, 23 (2018).

72. PATO: Phenotypic Quality Ontology.

73. Jackson, R. C. *et al.* ROBOT: A Tool for Automating Ontology Workflows. *BMC Bioinformatics* **20**, 407 (2019).

74. Ontology Access Kit (OAK).

75. Mallick, H. *et al.* Multivariable association discovery in population-scale meta-omics studies. *PLoS Comput Biol* **17**, e1009442 (2021).

76. Hastings, J. *et al.* ChEBI in 2016: Improved services and an expanding collection of metabolites. *Nucleic Acids Res* **44**, D1214–D1219 (2016).
77. Jacobs, A. *et al.* CAS Common Chemistry in 2021: Expanding Access to Trusted Chemical Information for the Scientific Community. *J. Chem. Inf. Model.* **62**, 2737–2743 (2022).
78. Kim, S. *et al.* PubChem 2023 update. *Nucleic Acids Research* **51**, D1373–D1380 (2023).
79. Bairoch, A. The ENZYME database in 2000. *Nucleic Acids Research* **28**, 304–305 (2000).
80. The Gene Ontology Consortium. The Gene Ontology Resource: 20 years and still GOing strong. *Nucleic Acids Research* **47**, D330–D338 (2019).
81. Köhler, S. *et al.* The Human Phenotype Ontology in 2021. *Nucleic Acids Research* **49**, D1207–D1217 (2021).
82. Seal, R. L. *et al.* Genenames.org: the HGNC resources in 2023. *Nucleic Acids Research* **51**, D1003–D1009 (2023).
83. Bansal, P. *et al.* Rhea, the reaction knowledgebase in 2022. *Nucleic Acids Research* **50**, D693–D700 (2022).
84. Haghbakhsh, R., Raeissi, S. & Duarte, A. R. C. Group contribution and atomic contribution models for the prediction of various physical properties of deep eutectic solvents. *Sci Rep* **11**, 6684 (2021).

Click here to access/download
Supplementary Material
Santangelo_etal_supplementary_REVISIONS2.docx
