# Supplementary material for: KG-Microbe: Building modular and scalable knowledge graphs for microbiome and microbial sciences
Source: Gigascience. 2026 Jul 10;15:giag077. doi: 10.1093/gigascience/giag077 (PMC13536490; doi:10.1093/gigascience/giag077)
Supplement: Supplementary file 8 [file giag077_reviewer_1_report_original_submission.pdf]

Reviewer Report

Title: KG-Microbe - Building Modular and Scalable Knowledge Graphs for Microbiome and Microbial Sciences

Version: Original Submission **Date:** 4/1/2025

Reviewer name: Reviewer 1

Reviewer Comments to Author:

This paper presents a KG (KG-Microbe) comprising four components that integrate microbial data, presenting a resource with the potential to support further biomedical research. The KG was evaluated by analyzing its ability to provide insights on specific topics and training a machine learning model for predictive tasks.While the paper tackles a relevant issue and could serve as a valuable resource for the community, I have some concerns regarding the scientific rigor and clarity of the methods, as well as the presentation of results. Below, I provide more specific comments:- I found the biological background insufficient for readers unfamiliar with key biological terms like "taxon" or "strain." Including brief explanations and additional references for established resources would improve accessibility. For example, the sentence "Information about the structure and function of microbes and their communities is available in a range of publicly available sources. Microbial traits and functional attributes can be discovered with laboratory experiments or computational predictions using publicly available data." lack citations that support these claims.Additionally, the comparison between KG-Microbe and existing microbial KGs is not sufficiently clear. A comparative table highlighting key features and differences would help establish the novelty and relevance of KG-Microbe. A more detailed description of each existing KG's characteristics would also be beneficial.- The organization of the "Data Description" section somehow overlaps with the "Methods" section, leading sometimes to redundancy and confusion. Additionally, some text in the "Data Description" refers to content that appears later in the paper, disrupting the logical flow.Furthermore, some paragraphs in section 2.1.2 would benefit from including concrete examples to enhance clarity. For example, the following sentence is not clear to me: "Ecological phenotypes such as pathogenicity in a given host (e.g. "plant" or "human"), biosafety level (BSL) and the sources from which a given strain has been found, referred to as an isolation source, are also modeled."- I have several concerns about the evaluation.The approach to CQs is unclear. Typically, CQs are formulated in natural language, translated into formal graph queries (e.g., SPARQL), and evaluated with appropriate metrics like accuracy. Here, the focus appears to be mainly on butyrate production, which may not fully reflect the broader scope of the KG. I believe the authors should expand the scope of CQs and detail the evaluation process.The evaluation through ML also raises questions. Generating semantic representation using binary features over more recent approaches like KG embeddings or Graph Neural Networks requires justification. Additionally, while the SHAP value analysis is promising, Figure 7 is not easily interpretable â€” displaying enzyme labels instead of codes could improve readability.- Justifications for certain methodological choices are missing. For example, the exclusion of textual information (subsection 6.2.1) and the decision not to map pathways to ontologies (subsection 6.2.2) are not explained.The rationale for excluding negative information â€” critical in KGs

operating under an open-world assumption â€” should be addressed. Distinguishing between missing information and something negative is very relevant.The scalability and reliability of manual mappings between taxonomies are concerning, given their inherent limitations. More automated mapping strategies should be considered.While the methods are detailed, some subsections feel overly exhaustive. Summarizing parts of the methods and incorporating more visual representations to illustrate modeling choices would enhance readability.- Finally, as a broader consideration, it is unclear to me whether KG-Microbe should be considered a framework or solely a KG. If positioned as a framework, it should be clear how it could generalize to other datasets beyond the ones used. Currently, the methods appear highly tailored to specific data sources/ databases.Minor comments:- Figure 2 shows nodes like phRange and temperature without connections. Are these nodes meant to be isolated?- Pages 6, 10, and 26 have excessive blank spaces.- The hyperlink in reference 12 seems broken (I think the correct link is https://zenodo.org/records/15026977).- Figure 4 is split across pages.- Formatting issues are present on page 33.- The title of section 2.1.5 appears overly generic.- An overview of the methodology before subsection 6.1 could help readers.- There is repetition in subsection 6.2.7: "Information about whether a given protein is reviewed or unreviewed is ingested directly from UniProt, though not included in the graphs (...) Provenance of whether a protein is from SwissProt or TrEMBL is recorded in the "reviewed" field of the raw data, though not included in the final graph.".- On page 38, correct the following "Biological process, molecular function, and cellular component GO terms are extracted from the "go" field, excluding any text or brackets, and linked to microbes via the predicate "biolink:participates_in", "biolink:participates_in", or "biolink:located_in", respectively.".- On page 42, the sentence "Another semantic representation involving UniProt data was microbes capable of defined sets of EC numbers, allowing for one missing annotated reaction." needs clarification.- I would reorganize the subsections in section 6 to clarify that subsections 6.1 and 6.2 cover KG construction while subsections 6.3, 6.4 and 6.5 focus on evaluation.

Level of Interest

Please indicate how interesting you found the manuscript: Choose an item.

Quality of Written English

Please indicate the quality of language in the manuscript: Choose an item.

Declaration of Competing Interests

Please complete a declaration of competing interests, considering the following questions:

- Have you in the past five years received reimbursements, fees, funding, or salary from an organisation that may in any way gain or lose financially from the publication of this manuscript, either now or in the future?
- Do you hold any stocks or shares in an organisation that may in any way gain or lose financially from the publication of this manuscript, either now or in the future?

- Do you hold or are you currently applying for any patents relating to the content of the manuscript?
- Have you received reimbursements, fees, funding, or salary from an organization that holds or has applied for patents relating to the content of the manuscript?
- Do you have any other financial competing interests?
- Do you have any non-financial competing interests in relation to this paper?

If you can answer no to all of the above, write 'I declare that I have no competing interests' below. If your reply is yes to any, please give details below.

I declare that I have no competing interests.

I agree to the open peer review policy of the journal. I understand that my name will be included on my report to the authors and, if the manuscript is accepted for publication, my named report including any attachments I upload will be posted on the website along with the authors' responses. I agree for my report to be made available under an Open Access Creative Commons CC-BY license (http://creativecommons.org/licenses/by/4.0/). I understand that any comments which I do not wish to be included in my named report can be included as confidential comments to the editors, which will not be published.

Choose an item.

To further support our reviewers, we have joined with Publons, where you can gain additional credit to further highlight your hard work (see: https://publons.com/journal/530/gigascience). On publication of this paper, your review will be automatically added to Publons, you can then choose whether or not to claim your Publons credit. I understand this statement.

Yes Choose an item.
