# Supplementary material for: KG-Microbe: Building modular and scalable knowledge graphs for microbiome and microbial sciences
Source: Gigascience. 2026 Jul 10;15:giag077. doi: 10.1093/gigascience/giag077 (PMC13536490; doi:10.1093/gigascience/giag077)
Supplement: Supplementary file 11 [file giag077_reviewer_2_report_original_submission.pdf]

**Reviewer Report**

**Title: KG-Microbe - Building Modular and Scalable Knowledge Graphs for Microbiome and Microbial Sciences**

**Version: Original Submission** **Date:** 4/10/2025

**Reviewer name: Reviewer 2**

**Reviewer Comments to Author:**

This manuscript introduces KG-Microbe, a knowledge graph (KG) framework developed to integrate microbial and host-associated data from diverse sources. The authors propose a modular and scalable solution supporting integrative analyses in microbiome research, addressing gaps in existing KGs which are limited in either taxonomic coverage, integration depth, or the functional and ecological context. KG-Microbe incorporates extensive microbial taxonomies, traits, phenotypes, genomic annotations, and environmental interactions to provide powerful tools for querying complex microbial data and generating predictive insights in both biomedical and environmental contexts.The paper addresses an important and emerging area in microbial bioinformatics. Given the complexity of microbiome data and its integration needs, the development of modular, interoperable knowledge graphs provides substantial value for both computational and experimental communities.KG-Microbe is comprehensive, integrating data across a range of taxonomies, traits, phenotypic and genomic features, experimental assays, media information, and disease associations. The incorporation of diverse data sources such as UniProt, BacDive, and Disbiome significantly enhances its value.The authors present a transparent description of data ingestion, processing pipelines, semantic modeling (Biolink model compliance), and knowledge integration. This transparency is essential for reproducibility and for the KG's adoption by the wider research community.Evaluations through competency questions demonstrate the KG's utility clearly, particularly in microbial disease contexts (IBD and Parkinson's disease) and ecological predictions (temperature preferences). Additionally, leveraging semantic reasoning and ML explainability (SHAP values) are strong and compelling use-cases.Major comments:- While the authors provide compelling competency questions, a comparative analysis with alternative microbial KG implementations or common microbial databases (e.g., MicrobiomeKG, MiKG4Md, or AGORA2) would help clearly position KG-Microbe within the existing landscape.- Information on query response times, KG size impact on performance, or processing scalability when updating the KG (particularly with UniProt frequent updates) would enhance credibility and applicability to large-scale projects.- The manuscript acknowledges potential inaccuracies in UniProt's predicted annotations, particularly butyrate production. More detailed discussions on how data inaccuracies were handled or mitigated (beyond Monte Carlo simulations and validation against experimentally supported data from BacDive) would reinforce confidence in the reliability and accuracy of KG-Microbe predictions.Minor comments:- Figures are generally clear, but some visualizations (e.g., Figures 4 and 5) would benefit from better resolution or simplified presentation. Specifically, the treemap visualizations (Fig. 5) are somewhat difficult to interpret quickly and clearly.- Although method details are robustly described, usability documentation such as example queries or a brief tutorial could facilitate immediate practical uptake by other

researchers.- Clearly highlighting more explicitly how KG-Microbe might evolve, particularly integration with other pathway-centric resources, could clarify the roadmap for its future improvement and sustainability.

**Level of Interest**

Please indicate how interesting you found the manuscript: Choose an item.

**Quality of Written English**

Please indicate the quality of language in the manuscript: Choose an item.

**Declaration of Competing Interests**

Please complete a declaration of competing interests, considering the following questions:

- Have you in the past five years received reimbursements, fees, funding, or salary from an organisation that may in any way gain or lose financially from the publication of this manuscript, either now or in the future?
- Do you hold any stocks or shares in an organisation that may in any way gain or lose financially from the publication of this manuscript, either now or in the future?
- Do you hold or are you currently applying for any patents relating to the content of the manuscript?
- Have you received reimbursements, fees, funding, or salary from an organization that holds or has applied for patents relating to the content of the manuscript?
- Do you have any other financial competing interests?
- Do you have any non-financial competing interests in relation to this paper?

If you can answer no to all of the above, write 'I declare that I have no competing interests' below. If your reply is yes to any, please give details below.

I declare that I have no competing interests

I agree to the open peer review policy of the journal. I understand that my name will be included on my report to the authors and, if the manuscript is accepted for publication, my named report including any attachments I upload will be posted on the website along with the authors' responses. I agree for my report to be made available under an Open Access Creative Commons CC-BY license (http://creativecommons.org/licenses/by/4.0/). I understand that any comments which I do not wish to be included in my named report can be included as confidential comments to the editors, which will not be published.

Choose an item.

To further support our reviewers, we have joined with Publons, where you can gain additional credit to further highlight your hard work (see: https://publons.com/journal/530/gigascience). On publication of this paper, your review will be automatically added to Publons, you can then choose whether or not to claim your Publons credit. I understand this statement.

Yes Choose an item.
