# Supplementary material for: KG-Microbe: Building modular and scalable knowledge graphs for microbiome and microbial sciences
Source: Gigascience. 2026 Jul 10;15:giag077. doi: 10.1093/gigascience/giag077 (PMC13536490; doi:10.1093/gigascience/giag077)
Supplement: Supplementary file 13 [file giag077_reviewer_3_report_original_submission.pdf]

Reviewer Report

Title: KG-Microbe - Building Modular and Scalable Knowledge Graphs for Microbiome and Microbial Sciences

Version: Original Submission **Date:** 4/15/2025

Reviewer name: Reviewer 3

Reviewer Comments to Author:

In this paper, the authors introduce KG-Microbe, a framework that integrates multiple data sources to enhance the understanding of heterogeneous microbial interactions using knowledge graphs (KGs). The KG is structured following the Biolink Model, and four versions of the KG at different resolutions are made available. By combining diverse sources, the framework yields a rich and comprehensive database of interactions, ranging from functional characterization to disease associations. The paper presents three distinct applications of KG-Microbe. While the manuscript is generally well written and the framework shows promise, several major issues need to be addressed:1. In the first application, the authors assess the accuracy of KG-Microbe by comparing its annotations with those from Vital et al. However, the discussion lacks clarity regarding annotations identified exclusively by KG-Microbe or exclusively by Vital et al. Are these to be interpreted as false positives and false negatives, respectively? This distinction should be made explicit. Furthermore, the issue of redundant relations in UniProt is briefly mentioned, but requires a more in-depth discussionâ€”particularly regarding its broader implications for knowledge graph quality and reliability.2. The third application is difficult to interpret. The authors should clearly describe the types of data extracted from the KG and explain how these data are utilized within the machine learning pipeline. Currently, the explanation is too vague. Additionally, Figure 7 lacks clarity and should be revised with more detailed labels and explanations to improve reader comprehension.3. Although the authors reference other microbial knowledge graphs, a more systematic comparison is warranted. A comparative table summarizing the features of KG-Microbe in relation to existing databases would significantly improve the reader's understanding of its novel contributions and advantages.4. To enhance usability, the software documentation should include several example queries to help users effectively explore and utilize the knowledge graph. Additionally, the provided notebooks should be more thoroughly documented.

Level of Interest

Please indicate how interesting you found the manuscript: Choose an item.

Quality of Written English

Please indicate the quality of language in the manuscript: Choose an item.

Declaration of Competing Interests

Please complete a declaration of competing interests, considering the following questions:

- Have you in the past five years received reimbursements, fees, funding, or salary from an organisation that may in any way gain or lose financially from the publication of this manuscript, either now or in the future?
- Do you hold any stocks or shares in an organisation that may in any way gain or lose financially from the publication of this manuscript, either now or in the future?
- Do you hold or are you currently applying for any patents relating to the content of the manuscript?
- Have you received reimbursements, fees, funding, or salary from an organization that holds or has applied for patents relating to the content of the manuscript?
- Do you have any other financial competing interests?
- Do you have any non-financial competing interests in relation to this paper?

If you can answer no to all of the above, write 'I declare that I have no competing interests' below. If your reply is yes to any, please give details below.

I declare that I have no competing interests

I agree to the open peer review policy of the journal. I understand that my name will be included on my report to the authors and, if the manuscript is accepted for publication, my named report including any attachments I upload will be posted on the website along with the authors' responses. I agree for my report to be made available under an Open Access Creative Commons CC-BY license (http://creativecommons.org/licenses/by/4.0/). I understand that any comments which I do not wish to be included in my named report can be included as confidential comments to the editors, which will not be published.

Choose an item.

To further support our reviewers, we have joined with Publons, where you can gain additional credit to further highlight your hard work (see: https://publons.com/journal/530/gigascience). On publication of this paper, your review will be automatically added to Publons, you can then choose whether or not to claim your Publons credit. I understand this statement.

Yes Choose an item.
