# Supplementary material for: KG-Microbe: Building modular and scalable knowledge graphs for microbiome and microbial sciences
Source: Gigascience. 2026 Jul 10;15:giag077. doi: 10.1093/gigascience/giag077 (PMC13536490; doi:10.1093/gigascience/giag077)
Supplement: Supplementary file 15 [file giag077_reviewer_4_report_original_submission.pdf]

Reviewer Report

Title: KG-Microbe - Building Modular and Scalable Knowledge Graphs for Microbiome and Microbial Sciences

Version: Original Submission **Date:** 4/25/2025

Reviewer name: Reviewer 4

Reviewer Comments to Author:

Reproducibility report for: KG-Microbe - Building Modular and Scalable Knowledge Graphs for Microbiome and Microbial SciencesJournal: GigaScienceID number/DOI: GIGA-D-25-00093Reviewer(s): Laura Caquelin, Department of Clinical Neuroscience, Karolinska Institutet, Sweden [Wrote the summary of the study and the scope of reproducibility section and reviewed the final report.]Tobias WÃ¤ngberg, Department of Clinical Neuroscience, Karolinska Institutet, Sweden [Worked on reproducing the results and contributed to writing the report.]--1. Summary of the StudyThe study introduces the KG-Microbe framework, which harmonizes bacterial and archaeal data into a knowledge graph (KG). Evaluations demonstrate its accuracy and effectiveness and its utility in human disease like inflammatory bowel and Parkinson's diseases. KG-Microbe is a flexible tool for both human and machine learning analyses to uncover microbial interactions and their mechanisms.--2. Scope of reproducibilityAccording to our assessment the primary objective is: to demonstrate the effectiveness of the KG-Microbe framework in integrating microbial data and understanding its role in human diseases, specifically inflammatory bowel disease (IBD) and Parkinson's disease (PD).- Outcome: The framework effectively identifies microbial functions, such as butyrate production, and their associations with disease outcomes- Analysis method outcome: Microbial data were analyzed using the KG-Microbe knowledge graph, with a chi-squared test applied to compare disease annotations and the proportion of butyrate producers.- Main result: Presented in Figure 6C. "We found that a significantly higher number of butyrate producing microbes were associated with a decreased likelihood of both IBD and PD (p = 1e-142, p = 2e-288 respectively)".--3. Availability of Materialsa. Data- Data availability: Open- Data completeness: Complete after requesting some missing files from the authors.- Access Method: Repository- Repository: https://github.com/bsantan/kg-microbe-paper/tree/main- Data quality: The data files have been shared and appear sufficient for running the analyses. However, no metadata is provided to describe the content, structure, or origin of the files which limits interpretability and reusability.b. Code- Code availability: Open- Programming Language(s): Python- Repository link: https://github.com/bsantan/kg-microbe-paper/tree/main- License: BSD-3-Clause license- Repository status: Public- Documentation: Clear documentation in README file.--4. Computational environment of reproduction analysis- Operating system for reproduction: MacOS 15.1- Programming Language(s): Python- Code implementation approach: Writing script based on shared code- Version environment for reproduction: Python 3.12--5. Results5.1 Original study results- Results 1: The main results are presented

in Figure 6C. The result of a chi-squared test confirmed that increased levels of Butyrate were associated with increased likelihood of IBD and PD. The reported p-values were 1e-142, p = 2e-288 respectively.5.2 Steps for reproduction-> Run the code Classification_gold_standard_comparison.py- Issue 1: Missing file merged-kg_edges_noEC.tsv and folder kg-microbe-biomedical-function-cat-- Resolved: After contacting the authors, the missing files were shared on the Github repository.- Issue 2: Error message: could not find directory "./Input_Files/kg-microbe-biomedical-function/merged-kg_edges.tsv"--Resolved: Changed the directory to "Input_Files/data/merged/kg-microbe-biomedical-function/merged-kg_edges.tsv" after extracting KGMicrobe-biomedical-function-20250222.tar.gz located in folder Input_Files.- Issue 3: The script could not be run since it requires too much memory (running on 24 GB ram MacBook Pro 2024). The variable data_edges is of size > 16 GB, making copies of this variable in the code will cost a lot of memory, and eventually force the script to terminate.--Resolved: Rewrote the script Classification_gold_standard_comparison.py with the following modifications to avoid making local copies of variables requiring a lot of memory(i) Instead of calling sub-functions get_disease_pairs, subset_by_features, find_microbes_strain, remove_conflicting_directionality the code was directly added to the main script.(ii) Instead of running the main script in one go the script was rewritten by splitting it up and storing intermediate results in files- Issue 4: The file ncbitaxon_nodes.tsv , which is required to run the script needed to generate Gold_Standard_Species_Overlap_butyrate_produces.csv, is missing.-- Resolved: The file ncbitaxon_nodes.tsv is required to run another script named Process_competency_question.py to produce Gold_Standard_Species_Overlap_butyrate_produces.csv. After contacting the authors, the missing file was shared via email. However the computer used in this analysis did not have sufficient memory requirements to execute the script. Therefore the file Gold_Standard_Species_Overlap_butyrate_produces.csv was sent directly by the authors via email.5.3 Statistical comparison Original vs Reproduced results- Results: The results obtained were p-value 1e-142 for IBD and p-value 8e-293 for PD (see screenshot from console).- Comments: The slight discrepancy for the IBD p-value may be due to different rounding errors between computers used to compute the results.- Errors detected: -- Statistical Consistency: The p-value for IBD is consistent. The p-value for PD differs slightly; 2e-288 in the manuscript compared to 8e-293 in the reproducibility analysis.---6. Conclusion- Summary of the computational reproducibility reviewThe aim of this report was to reproduce the p-value of a chi-squared test to confirm that increased levels of Butyrate are associated with increased likelihood of IBD and PD. The results were reproduced. The p-value for IBD matched the result in the paper. The p-value for PD differed slightly (2e-288 in the manuscript compared to 8e-293 in the reproducibility analysis).- Recommendations for authorsThe study was reproducible because of access to the data uploaded in the Github repository or shared by email. To ensure that the study remains fully reproducible in the future, the following recommendations are strongly advised:-- The authors should upload all required files to the GitHub repository.-- All required packages required to run the script should be included, the package duckdb is missing from the readme file.-- The required Python package 'catboost' is currently not compatible with Python 3.13 or more recent versions. A note about this in the README file would be helpful for reproducibility.-- The script requires a lot of memory to run. For computers with 24 GB ram or less the script is not able to execute. To improve reproducibility the authors could rewrite the script but avoiding creating copies of large variables and deleting unused variables. In addition, a comment in the README about hardware requirements would be helpful.-- It would be helpful to include

accompanying metadata files, for the datasets used or generated by the scripts, that explain:--- The definition of each variable name.--- The origin of each dataset (raw, processed, etc).--- Any preprocessing steps applied before analysis.

Level of Interest

Please indicate how interesting you found the manuscript: Choose an item.

Quality of Written English

Please indicate the quality of language in the manuscript: Choose an item.

Declaration of Competing Interests

Please complete a declaration of competing interests, considering the following questions:

- Have you in the past five years received reimbursements, fees, funding, or salary from an organisation that may in any way gain or lose financially from the publication of this manuscript, either now or in the future?
- Do you hold any stocks or shares in an organisation that may in any way gain or lose financially from the publication of this manuscript, either now or in the future?
- Do you hold or are you currently applying for any patents relating to the content of the manuscript?
- Have you received reimbursements, fees, funding, or salary from an organization that holds or has applied for patents relating to the content of the manuscript?
- Do you have any other financial competing interests?
- Do you have any non-financial competing interests in relation to this paper?

If you can answer no to all of the above, write 'I declare that I have no competing interests' below. If your reply is yes to any, please give details below.

1. no 2. no 3. no 4. no 5. no 6. no

I agree to the open peer review policy of the journal. I understand that my name will be included on my report to the authors and, if the manuscript is accepted for publication, my named report including any attachments I upload will be posted on the website along with the authors' responses. I agree for my report to be made available under an Open Access Creative Commons CC-BY license (http://creativecommons.org/licenses/by/4.0/). I understand that any comments which I do not wish to be included in my named report can be included as confidential comments to the editors, which will not be published.

Choose an item.

To further support our reviewers, we have joined with Publons, where you can gain additional credit to further highlight your hard work (see: https://publons.com/journal/530/gigascience). On publication of this paper, your review will be automatically added to Publons, you can then choose whether or not to claim your Publons credit. I understand this statement.

Yes Choose an item.
