# Supplementary material for: KG-Microbe: Building modular and scalable knowledge graphs for microbiome and microbial sciences
Source: Gigascience. 2026 Jul 10;15:giag077. doi: 10.1093/gigascience/giag077 (PMC13536490; doi:10.1093/gigascience/giag077)
Supplement: Supplementary file 16 [file giag077_reviewer_4_report_revision_1.pdf]

Reviewer Report

Title: KG-Microbe - Building Modular and Scalable Knowledge Graphs for Microbiome and Microbial Sciences

Version: Revision 1 **Date:** 11/6/2025

Reviewer name: Reviewer 4

Reviewer Comments to Author:

Reproducibility report for: KG-Microbe - Building Modular and Scalable KnowledgeGraphs for Microbiome and Microbial SciencesJournal: GigascienceID number/DOI: GIGA-D-25-00093Reviewer(s): Tobias WÃ¤ngberg, Department of Clinical Neuroscience, KarolinskaInstitutet, Sweden [Worked on checking the authors' modifications and writing thereport.]Laura Caquelin, Department of Clinical Neuroscience, Karolinska Institutet, Sweden[Wrote and reviewed the report]--1. ContextThis report refers to a second assessment of the computational reproducibility of thearticle GIGA-D-25-00093, following a revision by the authors after the first round ofreview.The scope of the computational reproducibility review was to reproduce the outcome ofchi-squared tests comparing disease annotations association with the proportion ofbutyrate producers on a 2x2 contingency table. The results can be found in Figure 6C ofthe main manuscript.--2. Changes since the first reviewThe authors have uploaded additional data files to the Github repository and updated thereadme file according to the comments of the first computational reproducibility review.Since all modifications made by the authors concerned only the GitHub repository andthe results of the reproducibility analysis remain unchanged from the first review, we didnot re-run the full reproducibility analysis.But the authors did not provide a specific response to the results differences observed inthe first report in their reply. So we wanted to understand and during this second review,we re-checked the chi-squared test results for Parkinson's disease using the contingencytable provided in the manuscript. Running the test in R produced the same chi-squaredstatistic and p-value as reported by the authors, which suggests that the discrepancyobserved in the first review likely came from an issue when executing the original code,probably a difference in the contingency table.(see Screenshot R console)--6. Conclusion- Follow-up on previous recommendations:In the first round of review, we noted several recommendations about:-- Uploading files in the Github repository. The authors have added thenecessary files to the GitHub or instructions for how to download them.However, However, several intermediate files required to reproduce thefull workflow remain unavailable. For example, the intermediate file"Gold_Standard_Species_Overlap_butyrate_produces.csv" could not bereproduced due to computational limitations and was shared by theauthors via email instead of being made publicly available.To allowindependent reproduction of the analyses, including those in the kg-microbe-paper repository, we recommend that all intermediate files beshared in the public repository or that clear instructions be provided togenerate them.-- Updating the Readme file with environment information. The authorshave updated the readme file with the required Python packages. But theydid not add

information on hardware requirements (for computers with 24GB ram or less the script is not able to execute). And we encourage theauthors to include the hardware requirements in the README file.--Add metadata to the dataset. The information provided by the authorsmainly describes the data processing workflow and file structure, not themetadata describing the datasets. For better transparency and FAIRcompliance, we recommend that the authors include explicit metadatadocumentation (for example, a data dictionary or metadata tabledescribing each dataset, variable names, formats, and data provenance).- Summary of the second computational reproducibility reviewThe results of the original study were reproduced during the first review with a minornumerical error. The result was consistent for inflammatory bowel disease, but differedslightly for Parkinson's disease (PD):- The reproduced p-value for PD was 8e-293 as opposed to the reported p-value of2e-288- The reproduced value of the test statistic for PD was 1337 whereas the reportedvalue was 1317.These numerical discrepancies are minor. We verified the chi-squared results with thereported contingency table (figure 6C) in R and obtained the same chi-squared statisticand p-value as reported in the manuscript, which suggests that the difference observedin the first review likely resulted from an issue when running the original code to generatethe contingency table. However, as intermediate files are not shared in the repository, wewere unable to re-run the complete analysis to confirm the exact source of thediscrepancy. We therefore recommend that the authors double-check this part of theanalysis to ensure full computational reproducibility.

Level of Interest

Please indicate how interesting you found the manuscript: Choose an item.

Quality of Written English

Please indicate the quality of language in the manuscript: Choose an item.

Declaration of Competing Interests

Please complete a declaration of competing interests, considering the following questions:

- Have you in the past five years received reimbursements, fees, funding, or salary from an organisation that may in any way gain or lose financially from the publication of this manuscript, either now or in the future?
- Do you hold any stocks or shares in an organisation that may in any way gain or lose financially from the publication of this manuscript, either now or in the future?
- Do you hold or are you currently applying for any patents relating to the content of the manuscript?
- Have you received reimbursements, fees, funding, or salary from an organization that holds or has applied for patents relating to the content of the manuscript?
- Do you have any other financial competing interests?
- Do you have any non-financial competing interests in relation to this paper?

If you can answer no to all of the above, write 'I declare that I have no competing interests' below. If your reply is yes to any, please give details below.

1. No 2. No 3. No 4. No 5. No 6. No

I agree to the open peer review policy of the journal. I understand that my name will be included on my report to the authors and, if the manuscript is accepted for publication, my named report including any attachments I upload will be posted on the website along with the authors' responses. I agree for my report to be made available under an Open Access Creative Commons CC-BY license (http://creativecommons.org/licenses/by/4.0/). I understand that any comments which I do not wish to be included in my named report can be included as confidential comments to the editors, which will not be published.

Choose an item.

To further support our reviewers, we have joined with Publons, where you can gain additional credit to further highlight your hard work (see: https://publons.com/journal/530/gigascience). On publication of this paper, your review will be automatically added to Publons, you can then choose whether or not to claim your Publons credit. I understand this statement.

Yes Choose an item.
