# Supplementary material for: KG-Microbe: Building modular and scalable knowledge graphs for microbiome and microbial sciences
Source: Gigascience. 2026 Jul 10;15:giag077. doi: 10.1093/gigascience/giag077 (PMC13536490; doi:10.1093/gigascience/giag077)
Supplement: Supplementary file 17 [file giag077_reviewer_4_report_revision_2.pdf]

Reviewer Report

Title: KG-Microbe - Building Modular and Scalable Knowledge Graphs for Microbiome and Microbial Sciences

Version: Revision 2 **Date:** 3/18/2026

Reviewer name: Reviewer 4

Reviewer Comments to Author:

Reproducibility report for: KG-Microbe - Building Modular and Scalable Knowledge Graphs for Microbiome and Microbial SciencesJournal: GigascienceID number/DOI: GIGA-D-25-00093Reviewer(s): Tobias WÃ¤ngberg, Department of Clinical Neuroscience, Karolinska Institutet, Sweden [Worked on reproducing the results and contributed to writing the report.]Laura Caquelin, Department of Clinical Neuroscience, Karolinska Institutet, Sweden [Reviewed the final report.]---1. ContextThis report corresponds to a third assessment of the computational reproducibility of the article GIGA-D-25-00093, following a revision by the authors after the first round of review.The scope of the computational reproducibility review is to reproduce the outcome of chi-squared tests comparing disease annotations association with the proportion of butyrate producers on a knowledge graph. The results can be found in Figure 6C of the main manuscript.---2. Changes since the first reviewThe authors made several changes in response to the second review. All the previously unavailable intermediate files have now been uploaded to the public repository, with clear README explanations. The repository structure was also reorganized so that inputs, intermediate outputs, and cached files are stored under a data folder.The computational setup was revised by migrating to uv, updating the Makefile, and redirecting the equilibrator cache to the project directory. The authors also added a memory-optimized version of the biomedical classification script intended to reduce out-of-memory problems on systems with more limited RAM.In addition, the authors expanded the documentation describing datasets and metadata, mainly through updates to the manuscript, supplementary methods, and README. Finally, they addressed the previously noted numerical discrepancies by attributing them to changing external dependencies, including updates to the NCBITaxon file and the Equilibrator API, and reported further code optimizations and caching improvements.---3. Availability of Materialsa. Data- Data availability: Open- Data completeness: Complete- Access Method: Repository- Repository: https://github.com/bsantan/kg-microbe-paper/tree/main-Data quality: Structuredb. Code- Code availability: Open- Programming Language(s): Python- Repository link:https://github.com/bsantan/kg-microbe-paper/tree/main- License: BSD 3-Clause License- Repository status: Public- Documentation: Clear---4. Computational environment of reproduction analysis- Operating system for reproduction: MacOS 26.2- Programming Language(s): Python- Code implementation approach: Using shared code- Version environment for reproduction: Python 3.12.12---5. Results5.1 Original study results- Results 1: The main results are presented in Figure

6C. The result of a chi-squared test confirmed that increased levels of Butyrate were associated with decreased likelihood of IBD and PD. The reported p-values were 1e-142, p = 2e-288 respectively.5.2 Steps for reproduction-> Cloning repository and set up environmentThe repository was cloned from GitHub, and the Python environment was created using uv, by running the following terminal commands.curl -LsSf https://astral.sh/uv/install.sh | shgit clone https://github.com/bsantan/kg-microbe-paper.gitcd kg-microbe-papersource $HOME/.local/bin/envuv sync-> Download and prepare the required input data.The make all terminal command below was used to download the KG-Microbe archive and ontology data, extract the relevant files, and generate the derived edge tables required for the downstream analyses.uv run make all-> Run analysis scripts.The analysis scripts were run by executing the following terminal commands.uv run python src/gut_microbes_competencies.pyuv run python src/Process_competency_questions.pyuv run python src/Gold_standard_Competency_analysis.pyuv run python src/Classification_gold_standard_comparison.py-> Inspect the final reproduced summary files.The final results were obtained from the generated IBD and PD summary files.cat data/Intermediate_Files/IBD_Classification_butyrate_producers_summary.csvcat data/Intermediate_Files/PD_Classification_butyrate_producers_summary.csv5.3 Statistical comparison Original vs Reproduced results- Reproduced results: For both conditions, the counts of "decreased" and "increased" values in the contingency table reproduced underlying the Chi-square test differ. For inflammatory bowel disease, the reproduced chi-squared statistic was 672 with a p-value of 4e-148. For Parkinson's disease, the reproduced chi-squared statistic was 1064 with a p-value of 3e-233. In the manuscript, the corresponding reported p-values were approximately 1e-142 for inflammatory bowel disease and 2e-288 for Parkinson's disease.(See IBD_Classification_butyrate_producers_summary.csv file and PD_Classification_butyrate_producers_summary.csv file).- Comments: -- Errors detected: -- Statistical Consistency: The statistical method used in the reproduction was consistent with the original study in that the same chi-squared comparison was reproduced from the authors' code and output files. The reproduced results remained qualitatively consistent with the original study in showing extremely small p-values and strong evidence of association. However, the numerical counts of "decreased" and "increased" values, the chi-squared statistics and p-values were not fully consistent with those reported in the manuscript, particularly for PD. Therefore, the statistical conclusions were directionally similar in terms of significance, but not numerically identical.---6. Conclusion- Summary of the third computational reproducibility reviewThe main computational workflow could be executed and the expected output files for the analysis were generated.The results were not fully reproducible even if they confirmed a highly statistically significant association for both inflammatory bowel disease and Parkinson's disease. The reproduced numerical results were not identical to those reported in the manuscript. For both conditions, the counts of "decreased" and "increased" values in the contingency table reproduced differ from the values in figure 6C, which may explain the observed differences in the test results.For inflammatory bowel disease, the reproduced chi-squared statistic was 672 with p-value 4e-148, compared with the reported chi-squared statistic of 647 and p-value of 1e-142. For Parkinson's disease, the reproduced chi-squared statistic was 1064 with p-value 3e-233, compared with the reported chi-squared statistic of 1317 and p-value of 2e-288.The reproduced numerical results in the present review also differed from those obtained in the previous round reviews, where the reproduced values obtained were a chi-squared statistic of 647 with

p-value 1e-142 for IBD and chi-squared statistic of 1338 with p-value 8e-293 for PD.This indicates that, although the qualitative conclusion of a highly significant association is robust, the exact numerical results are not fully stable across computational reproductions.- Follow-up on previous recommendations:The authors addressed several of the previous comments appropriately. In particular, the previously missing intermediate file is now publicly available in the repository, the file structure and documentation were improved, and a memory-optimized classification script was added. These changes substantially improved reproducibility compared with the previous round.We recommend that the authors rerun the analysis for Figure 6C, verify the dataset and intermediate outputs underlying the reported table values, and revise the manuscript table if needed so that the published values match the reproducible code output.

Level of Interest

Please indicate how interesting you found the manuscript: Choose an item.

Quality of Written English

Please indicate the quality of language in the manuscript: Choose an item.

Declaration of Competing Interests

Please complete a declaration of competing interests, considering the following questions:

- Have you in the past five years received reimbursements, fees, funding, or salary from an organisation that may in any way gain or lose financially from the publication of this manuscript, either now or in the future?
- Do you hold any stocks or shares in an organisation that may in any way gain or lose financially from the publication of this manuscript, either now or in the future?
- Do you hold or are you currently applying for any patents relating to the content of the manuscript?
- Have you received reimbursements, fees, funding, or salary from an organization that holds or has applied for patents relating to the content of the manuscript?
- Do you have any other financial competing interests?
- Do you have any non-financial competing interests in relation to this paper?

If you can answer no to all of the above, write 'I declare that I have no competing interests' below. If your reply is yes to any, please give details below.

1. no 2. no 3. no 4. no 5. no 6. no

I agree to the open peer review policy of the journal. I understand that my name will be included on my report to the authors and, if the manuscript is accepted for publication, my named report including any

attachments I upload will be posted on the website along with the authors' responses. I agree for my report to be made available under an Open Access Creative Commons CC-BY license (http://creativecommons.org/licenses/by/4.0/). I understand that any comments which I do not wish to be included in my named report can be included as confidential comments to the editors, which will not be published.

I agree to the open peer review policy of the journal

To further support our reviewers, we have joined with Publons, where you can gain additional credit to further highlight your hard work (see: https://publons.com/journal/530/gigascience). On publication of this paper, your review will be automatically added to Publons, you can then choose whether or not to claim your Publons credit. I understand this statement.

Yes Choose an item.
