# Supplementary material for: KG-Microbe: Building modular and scalable knowledge graphs for microbiome and microbial sciences
Source: Gigascience. 2026 Jul 10;15:giag077. doi: 10.1093/gigascience/giag077 (PMC13536490; doi:10.1093/gigascience/giag077)
Supplement: Supplementary file 18 [file giag077_supplementary_files.zip › Santangelo_etal_supplementary_REVISIONS3.docx]

**Supplementary Figure 1**

The KG-Microbe strain-level pan-proteome approach, which uses all representative proteins available for a given NCBI Taxonomy species. Each organism is represented as an NCBI Taxonomy identifier in the UniProt database^1^, and those are found via the uniprot2s3 repository^2^. Only those with associated proteomes are included in the transform. Proteins are marked as Unassembled or part of the Reference proteome, in which case Reference assemblies are prioritized if present. Only one is taken when more than one entry exists per protein to avoid duplication. Finally, only proteomes for organisms that have more than 1000 proteins are included.

**Supplementary Figure 2**

Sources included in each KG generated with the KG-Microbe framework. The type of each source is characterized by box color. Each KG includes ontologies, structured databases, experimental data from the literature, or genome annotations.

### 1. Supplementary Methods

### 1.1. KG Construction and Modeling

The sources ingested for each KG produced by the KG-Microbe framework are shown in Supplementary Figure 2. The version, data type, and modeling choices of all ingests are detailed in the following sections.

### 1.2. BacDive

The BacDive database^3^ is included in all KGs (Supplementary Figure 2). Information from BacDive was obtained using the API and was downloaded on October 3, 2023 as a JSON file. Each strain level taxon has a unique identifier within the JSON file. The strain identifier is the *“NCBI tax id”* subfield of the *“General”* subfield when present, otherwise is given a unique identifier according to the *“BacDive-ID”* field with the custom *“strain”* prefix (e.g., *“strain:bacdive_17187”*). The strain label is formed by combining the *“strain designation”* subfield of *“Name and taxonomic classification”,* the NCBITaxon ID, and the BacDive ID (e.g., “*bacdive_17187 as SH2 of NCBITaxon:1123511”*). The *“Physiology and metabolism”* field is used to find oxygen, salt concentration, metabolism, antibiotic, enzymatic activity, and API assay or antibiogram assay information. The *“oxygen tolerance”* subfield is used to generate nodes of 9 unique categories: *"oxygen:aerobe", "oxygen:aerotolerant", "oxygen:anaerobe", "oxygen:facultative_aerobe", "oxygen:facultative_anaerobe", "oxygen:microaerophile", "oxygen:microaerotolerant", "oxygen:obligate_aerobe",* or *"oxygen:obligate_anaerobe"*. Morphology information is captured from the *“Morphology”* subfield, within which are the *“cell morphology”* and *“pigmentation”* subfields. The *“colony morphology”* and *“multicellular morphology”* subfields were not ingested because they are free text fields. The *“cell morphology”* specifies the *“gram stain”* trait (*“negative”* or *“positive”*) which results in three unique nodes: “*gram_stain:positive”, “gram_stain:negative”, “gram_stain:variable”*. 18 unique values from the *“cell shape”* subfield were introduced to the graph with the *“biolink:has_phenotype”* predicate. Whether a microbe is motile or non-motile is sourced from the*“motility”* subfield, introducing the nodes *“motility:motile”* and *“motility:non_motile”.* Temperature categories are extracted from the *“culture temp”* subfield, where only the *“range”* subfield was used to obtain one of four categories (*“temperature:hyperthermophilic”, “temperature:mesophilic”, “temperature:psychrophilic”,* or *“temperature:thermophilic”*), and the quantitative measurements from the *“temperature”* subfield were not used. Salinity tolerance was introduced from the *“halophily_level”* subfield of the *“halophily”* subfield as 7 unique categories (“*extremely_halophilic”, “haloalkaliphilic”, “halophilic”, “halotolerant”, “moderately_halophilic”, “non_halophilic”, “slightly_halophilic”*). Chemical production or consumption results are obtained from the *“metabolite utilization”* subfield, where the associated ID comes from the *“Chebi-ID”* subfield when present, the *“biolink:produces”* predicate is used when a *“+”* is present in the *“utilization activity”* field, and the *“biolink:consumes”* predicate is used when a *“-”* is present in the *“utilization activity”* field. Chemical terms are mapped using provided mappings or by manual curation for the case of 9 terms (using the *“metabolite_mapping.json”* file). When no matching ChEBI exists, chemicals are represented with CAS-RN or KEGG identifiers according to BacDive (Table 1). Chemical production results are also found from the *“metabolite production”* subfield, where *“production”* is *“yes”*. Enzymatic activity is found in the *“enzymes”* subfield and modeled using the *“biolink:capable_of”* predicate from the microbe to the enzyme in the *“ec”* subfield when *“activity”* is *“+”*. Negative results from the *“enzymes”* subfield are not included in the graph (e.g. when *“activity”* is *“-”*). A microbe is considered sensitive to a given chemical when the *“is sensitive”* subfield within *“antibiotic resistance”* is *“yes”*, and resistant when *“is resistant”* is *“yes”*. The negative results of *“is sensitive”* or *“is resistant”* as *“no”* were not ingested. The sensitivity concentration from the “*sensitivity conc.”* subfield is not ingested but is available in the raw BacDive source files. Antibiotic resistance and sensitivity information is also taken from the antibiogram results in the *“antibiogram”* subfield, including resistance or sensitivity to chemicals such as *“chloramphenicol”*, *“amoxicillin”*, or *“ampicillin”*. This field provides a growth medium specified in the *“medium”* subfield and a range of chemical subfields with an associated value specifying the zone of inhibition (ZOI) diameter in millimeters, indicating the extent to which the chemical (an antibiotic) inhibits growth of the strain. Each individual antibiogram result is mapped to a standardized chemical identifier (using the *“metabolite_mapping.json”* file), and the value is returned as a single floating-point number where a range is represented by the mean (e.g. *“42-44”* becomes *“43”*) and a greater than inequality is represented by the corresponding minimum value (e.g. *“>50”* becomes *“50”*). Strains with ZOI values of less than 15mm for a given chemical are considered resistant to that antibiotic, and values of greater than 25mm are considered as sensitive to that chemical; data with values between 15 and 25mm are discarded. Microbes are linked to a chemical via the *“biolink:associated_with_sensitivity_to”* predicate when sensitive and the *“biolink:associated_with_resistant_to”* predicate when resistant. The *“metabolite tests”* and *“fatty acid profile”* subfields are not ingested. The *“Physiology and metabolism”* field is also used to find trophic type information, from the *“nutrition_type”* subfield, resulting in 28 unique categories from the *“type”* subfield (e.g., *“trophic_type:autotrophy”, “trophic_type:chemotrophy”, “trophic_type:lithoautotrophy”*). The *“spore_forming”* subfield was used to find whether or not a microbe can form spores based on the *“yes”* or *“no”* result (*“sporulation:spore_forming”* and *“sporulation:non_spore_forming”*).

Isolation source information was sourced from the *“isolation source categories”* subfield within the *“Isolation, sampling and environmental information”* subfield. BacDive models isolation sources in categories from 1 to 3, and all three categories were included as new nodes in the graph with the *“isolation_source”* prefix (e.g. *“isolation_source:environmental”*). These standardized values from BacDive were not mapped to any ontology (Table 1).

Biosafety level categories of 1, 2, or 3 were sourced from the *“Safety Information”/”risk assessment”* subfield. The *“biosafety level”* subfield was annotated with the corresponding safety level, which was introduced as a new nodes (e.g. *“BSL:1”)* and linked to the microbe via the *“biolink:associated_with”* predicate.

Culture medium information is found in the *“Culture and growth conditions”* field, where each medium is assigned a unique integer according to the MediaDive resources (e.g., *“medium:1”*). When the *“growth”* subfield is *“yes”*, an edge is added between the associated organism (e.g., *“NCBITaxon:1396, biolink:occurs_in, medium:1”*). Medium ingredients are  ingested from MediaDive (see the MediaDive section of the Methods).

Each API assay in BacDive assesses a number of enzymatic activities. Assays are represented with the chemical that the assay intends to test (*“chemical, biolink:occurs_in, assay”*) or the enzymatic activities assessed by the assay (*“enzymatic activity, biolink:assessed_by, assay”*). The combination of the API assay kit type (according to the name of a subfield starting with *“API”*), and the enzymatic activity, when annotated with a *“+”*, was used for each assay node. For example, the *“API zym”* field had 20 unique enzymatic activity results for a given taxon, one of which was being positive for *“Acid phosphatase”*, therefore a new node *“assay:API_zym_Acid phosphatase”* was linked to the associated microbe via the *“biolink:assesses”* predicate. Negative results were not included (e.g. when a given enzymatic activity was *“-”*). The antibiotic results are not consistent with the Clinical and Laboratory Standards Institute (CLSI)^4^ and European Committee on Antimicrobial Susceptibility Testing (EUCAST)^5^ and therefore not for clinical use.

Other fields that were not used from the BacDive JSON file were: *“Sequence information”, “External links”, “Publications”, “Reference”*, *“Sequence Information”, “Isolation”, “Literature”, “Fatty Acid Profile”, “Compound Production”, “Tolerance”, “Culture Temp.”, “Halophily”,* and *“Culture pH”* because they were in free text form.

### 1.3. Madin et al.

The Madin et al. ingest is included in all KGs (Supplementary Figure 2). Phenotype data is sourced from the *condensed_traits_NCBI.csv* from the Madin et al. GitHub repository^6^. Traits were introduced for each organism from the *“tax_id”* column which contained a NCBI Taxonomy ID. The *“metabolism”* column is used to extract information about oxygen tolerance, resulting in six unique categories (*"oxygen:aerobe", "oxygen:anaerobe", "oxygen:facultative_anaerobe", "oxygen:microaerophile", "oxygen:obligate_aerobe",* or *"oxygen:obligate_anaerobe")*. The two unique annotations of *“strictly anaerobic”* and *“anaerobic”* were collapsed into one KG node *“oxygen:anaerobe”*. The *“pathways”* column sources 100 unique pathways that are linked to the corresponding microbe with the *“biolink:capable_of”* predicate. Only a small number of pathways could be mapped to GO terms because they represent classes of metabolism as opposed to specific pathways (e.g. *methane oxidation* and *methylotrophy*), so we chose not to normalize this pathway information in the current version and relied on the normalization strategies of Madin et al. Therefore, no pathways are mapped to ontologies or external references but instead have the *“pathways”* prefix (e.g. *“pathways:thiosulfate_oxidation”*) (Table 1). The *“carbon_substrates”* column is used to identify chemicals that the corresponding taxa consumes, where all chemicals are mapped to a ChEBI identifier and linked to the microbe with the *“biolink:consumes”* predicate using named entity recognition and manual mappings for 6 unique terms (found in the *“chebi_manual_annotation.tsv”* file) (Table 1). 19 unique cell shapes are sourced from the *“cell_shape”* column (e.g. *“cell_shape:bacillus”*) and linked to the corresponding microbe with the *“biolink:has_phenotype”* predicate. Finally, environments from which microbes are found is sourced from the *“isolation_source”* column, which results in five unique categories that were not mapped to a standardized identifier (*“isolation_source:food_fermented”, “isolation_source:host_animal_ectotherm”, “isolation_source:host_animal_endotherm”, “isolation_source:host_animal_endotherm_intratissue”, “isolation_source:host_animal_endotherm_rumen”*), and the rest of which were mapped to the Environment Ontology (ENVO)^7^, ChEBI^8^, the Plant Ontology (PO)^9^, the Food Ontology (FOODON)^10^, the Phenotype and Trait Ontology (PATO)^11^, or the Uberon Ontology (UBERON)^12^ using the *data/conversion_tables/environments.csv* from Madin et al. (Table 1).

### 1.4. BactoTraits

The BactoTraits database^13^ is included in all KGs (Supplementary Figure 2). The BactoTraits database was downloaded as a tsv file (*BactoTraits_databaseV2_Jun2022.csv*). BactoTraits provides organismal traits for the same set of taxa at the strain or species level available in BacDive. All columns were mapped to unique prefix and categories using a LinkML structured yaml file called the *“custom_curies.yaml”*. Bacterial size information is binned into four qualitative labels of cell length and width (*very_low, low, mid,* or *high*) each represented as a node. Bacterial cells of length ≤1.3um are connected to the *“cell_length:very_low”* node, those of length 1.3um to 2um to the *“cell_length:low”* node, those of length 2um to 3um to the *“cell_length:mid”* node, and those of length ≥3um to the *“cell_length:high”* node. Similarly, bacterial cell width is represented in ranges of ≤0.5um (“*cell_width:very_low”),* 0.5um to 0.65um (“*cell_width:low”),* 0.65um to 0.9um (“*cell_width:mid”),* ≥0.9um (“*cell_width:high”)*. 9 unique cell shapes are sourced from BactoTraits. When a microbe was linked to multiple shapes in BactoTraits, KG-Microbe represents each trait as its own node (e.g. “*S_star_dumbell_pleomorphic*” in BactoTraits becomes *“cell_shape:star_dumbbell_pleomorphic”* and the individual linked nodes *“cell_shape:star”, “cell_shape:dumbell”, “cell_shape:pleomorphic”* in KG-Microbe). Growth conditions for each microbe were introduced from BactoTraits, including the optimal range for growth, the range in which growth is possible, and the ecological valence, or spread, of the range in which growth can occur. All of these ranges were binned into unique qualitative labels according to the representations of categories in BactoTraits (very low, low, mid1, mid2, mid3, mid4 and high). For example, salt concentration range nodes vary from *“NaCl_range:very_low”* (≤1% NaCl in BactoTraits) to *“NaCl_range:high”* (>8% NaCl in BactoTraits), whereas the spread of temperature range nodes vary from *“temp_opt:very_low”* (≤10°C) to *“temp_opt:high”* (>40°C), with intermediate ranges: *“temp_opt:low”* (10–22°C), *“temp_opt:mid1”* (22–27°C), *“temp_opt:mid2”* (27–30°C), *“temp_opt:mid3”* (30–34°C), and *“temp_opt:mid4”* (34–40°C). Trophic types were ingested as 10 categories directly from BactoTraits (e.g. *“trophic_type:autotrophy”*, *“trophic_type:chemotrophy”*, *“trophic_type:copiotrophy”*). Gram staining phenotypes were ingested directly from BactoTraits as *“gram_stain:positive”, “gram_stain:negative”, “gram_stain:variable”*, as was sporulation information as *“sporulation:spore_forming”* and *“sporulation:non_spore_forming”* and motility as *“motility:motile”* or *“motility:non_motile”.* The pigmentation of the microbial cell is recorded as 10 unique pigments (e.g, *“pigment:pink”, “pigment:black”, “pigment:carotenoid”*) that are not mapped to an ontology.

### 1.5. MediaDive

The MediaDive database^14^ is included in all KGs (Supplementary Figure 2). MediaDive was downloaded from the REST API v0.1.0 (https://mediadive.dsmz.de/rest/media). All unique media is taken from the *“id”* subfields of the *“data”* field. The DSMZ growth medium IDs that are introduced from BacDive correspond to those in MediaDive, where the *“name”* subfield serves as the node label for each medium. The medium type is extracted from the *“complex_medium”* subfield, which is a *“1”* if complex and *“0”* if not complex, resulting in two medium types: *“medium-type:complex”* and *“medium-type:defined”*. Given each unique medium ID, data from the MediaDive REST API is then used to find the strains that grow in the media using the *“medium-strains”* field, *“bacdive_id”* subfield. The BacDive ID is linked to the NCBI Taxonomy or unique strain node provided by BacDive using the *“biolink:occurs_in”* predicate. The *“medium”* field along with each unique medium ID is used to extract information about the medium content via the *“solutions”* subfield, where a new node is introduced using the *“id”* subfield (e.g. *“solution:1”*), and all ingredients are found in the *“recipe”* subfield using *“compound_id”*. The *“ingredient”* field along with each unique compound ID is used to represent each chemical as ChEBI, KEGG, PubChem, or CAS-RN IDs (using the *“ChEBI”, “KEGG-Compound”, “PubChem”,* or *“CAS-RN”* subfields respectively) (Table 1). When no chemical mapping was available, the custom *“ingredient”* prefix was used. Two new triples of the form *“solution, biolink:has_part, chemical”* and *“medium, biolink:has_part, solution”* are added using this content.

### 1.6. Rhea

The Rhea database^15^ is included in all KGs (Supplementary Figure 2). Biochemical reaction knowledge from the Rhea database is retrieved from Expasy flat files. The PyOBO Python package enables the construction of OBOs from structured databases and is used to retrieve relationships among biochemical reactions, and between biochemical reactions and chemicals. As part of PyOBO, the *“rhea-directions.tsv”* and *“rhea-relationships.tsv”* files are used to model the direction of each biochemical reaction and the hierarchy of biochemical reactions, respectively. Rhea terms have IDs in multiples of 4 starting at 10000, where the first is *“undirected”*, the second is *“left to right”*, the third is *“right to left”*, and the fourth is *“bidirectional”*. Each unique Rhea term is related to its parent term (except for the *“undirected”* term) via the *“biolink:subclass_of”* predicate. This pattern is used to model the input and output chemicals accordingly. The *“biolink:input_of”* and *“biolink:output_of”* predicates are used to link chemicals to reactions assigned with *“left to right”* or *“right to left”* directions. The *“biolink:participant_of”* predicate is used to link chemicals to reactions that are *“undirected”* or *“bidirectional”*. In addition to using the Rhea model from PyOBO, the *“rhea2ec.tsv”* and *“rhea2go.tsv”* files are downloaded as sources for relationships between biochemical reactions and molecular functions (modeled in the form *“molecular function, biolink:enables, biochemical reaction)*, and relationships between biochemical reactions and enzymatic activity (modeled in the form *“biochemical reaction, biolink:enabled_by, enzymatic activity*).

### 1.7. Ontologies

The Mondo Disease Ontology (MONDO) and human phenotype ontology (HPO) are only included in KG-Microbe-Biomedical and KG-Microbe-Biomedical-Function, while all other ontologies are included in KG-Microbe-Core and KG-Microbe-Function as well (Supplementary Figure 2).  Ontologies are downloaded as JSON or OWL files from the Open Biological and Biomedical Ontology (OBO) Foundry^16^. GO, HPO, and ENVO are transformed into edges and nodes using the *transform* function from KGX (version 2.4.2). EC, UPA, ChEBI, and MONDO are first transformed via the KGX library followed by post processing steps to correctly model the data for the purposes of KG-Microbe. In this step, the following prefixes are replaced from the original file: *“eccode”* with *“EC”* (from the EC JSON file), *“CAS”* with *“CAS-RN”* (from the ChEBI OWL file), *“OBO:UPa_UCR*” and *“OBO:UPa_UPA”* with *“UPA”* (from the UPA OWL file), and *“http://identifiers.org/hgnc/”* with *“HGNC”* (from the MONDO JSON file). UPA chemical reactions are mapped to Rhea terms using the *“xref”* field when possible, otherwise they keep the given ID with the *“UPA:UCR”* prefix (e.g. *“UPA:UCR00014”*). We derived the transitive relationships from UPA for triples of the form *“enzymatic reaction, part of, linear sub pathway”* and *“linear sub pathway, part of, pathway”*. In this process, enzymatic reactions were mapped to GO terms while pathways were prefixed with *“UPA:UPA”*, resulting in new triples of the form *“molecular function, biolink:enabled_by, metabolic pathway”* and *“biological process, biolink:related_to, metabolic pathway”*. Similarly, we derived transitive relationships for triples of the form *“chemical reaction, part of, enzymatic reaction”*, *“enzymatic reaction, part of, linear sub pathway*”, and *“linear sub pathway, part of, pathway”*. Here, new triples of the form *“biochemical reaction, biolink:part_of, metabolic pathway”* were introduced to the KG.

### 1.8. NCBI Taxonomy

As part of the KG-Microbe construction we generated a trimmed version of the NCBI Taxonomy^17^ that only included the single cell organisms of interest. The following eukaryotic or otherwise irrelevant taxonomic parent nodes and their descendants were removed: other sequences (NCBITaxon:28384), Viridiplantae (NCBITaxon:33090), Metazoa (NCBITaxon:33208), herbal medicine (NCBITaxon:1407750), mixed DNA libraries (NCBITaxon:1145094), mixed EST libraries (NCBITaxon:119167), mixed libraries (NCBITaxon:590738), Viruses (NCBITaxon:10239). Trimming was performed using ROBOT (ROBOT is an OBO Tool)^18^, a command line tool that supports filtering of OBOs in a logically consistent manner among other operations supporting ontology development.

### 1.9. UniProt

The microbial UniProt ingest is included in KG-Microbe-Function and KG-Microbe-Biomedical-Function (Supplementary Figure 2). Protein annotation data for KG-Microbe is sourced from UniProt proteomes, which are sets of proteins linked to a given taxon. The KG-Microbe pipeline extracts functional annotations from the UniProt REST API using parallelization with multiple CPU cores and produces a flat file for each NCBITaxon organism. Query fields used to construct the KG for both the microbial subset and humans are *“xref_proteomes”*, *“organism_id”*, *“accession*”, *“protein_names”*, *“ec”*, *“ft_binding”*, “*go”*, *“rhea”*, and *“cc_function”*. The infrastructure is supported in the uniprot2s3 GitHub repository, which outputs all organism specific files and stores them as a compressed file in S3. This serves as the raw protein functional annotation source data endpoint used to produce the transformed annotation data for the merged KGs (Figure 8). Provenance of whether a protein is from SwissProt (reviewed) or TrEMBL (unreviewed) is recorded in the *“reviewed”* field of the raw data, though not included in the final graph. The resulting UniProt raw files and other resources are then downloaded to prepare for extracting and modeling the information (the transform step in Figure 8). The *“organism_id”* field corresponds to an NCBI Taxonomy ID to which all functional information is linked. The *“accession”* field is used for each unique protein ID (e.g. *“UniprotKB:A0A1H0WGA5”)*, which is linked to the microbe via the *“biolink:derives_from”* predicate*.* The protein label is extracted from the *“protein_names”* field, where only names are included (in some cases more than one, semicolon delimited) and any EC IDs are excluded. GO terms for biological process, molecular function, and cellular component are extracted from the *“go”* field, excluding any text or brackets, and linked to microbes via the predicate “*biolink:participates_in”, “biolink:participates_in”,* or *“biolink:located_in”*, respectively. The GO term categories (GO-BP, GO-MF, GO-CC) are differentiated using the Ontology Access Kit (OAK)^19^. Biochemical reactions are extracted as Rhea IDs from the *“rhea”* field and linked to organisms via the *“biolink:participates_in”* predicate because all Rhea terms are undirected, and enzymatic activities as EC IDs from the *“ec”* field are linked to microbes via the *“biolink:enables”* predicate. Only ChEBI terms are extracted from the content in the *“ft_binding”* field to yield chemicals to which the corresponding protein binds via the *“biolink:binds”* predicate. We obtained UniProt proteome annotation data for 29,154 taxa represented by 56,670 proteomes, including 5,739 plasmid proteomes. After transform and merge, functional annotations were linked to 32,662 microbes across KG-Microbe-Function and KG-Microbe-Biomedical-Function. Organisms included in the UniProt ingest were limited to those associated with a reference or non-reference proteome and with more than 1,000 total proteins across all linked proteomes (Supplementary Figure 1). This protein-count cutoff was selected for computational tractability, allowing all entries to be processed in parallel during the transform step on a NERSC Perlmutter CPU node (512 GB total; 400 GB requested per job).

The human UniProt ingest is included in KG-Microbe-Biomedical and KG-Microbe-Biomedical-Function (Supplementary Figure 2). For the human query only, the *“gene_primary”* and *“cc_disease”* fields are also included. The *“gene_primary”* field contains a gene name which is mapped to a HUGO Gene Nomenclature Committee (HGNC) ID using MONDO in order to align with MONDO genes. The *“cc_disease”* field contains Online Mendelian Inheritance in Man (OMIM) IDs for diseases associated with the corresponding protein. These terms are mapped to MONDO terms using the *“xref”* fields of the MONDO OWL file. The protein is linked to the disease via the *“biolink:contributes_to”* predicate. Only the reference proteome was included for the human set of proteins (*“UP000005640”*), as well as unreviewed proteins. This results in potentially redundant human proteins which ensure that no annotation information is lost. The human subset of the UniProt database is linked to the corresponding NCBITaxon ID (NCBITaxon:9606) though it is not linked to the taxonomic hierarchy in the graphs.

### 1.10. Disbiome

The Disbiome^20^ ingest is included in the KG-Microbe-Biomedical and KG-Microbe-Biomedical-Function graphs (Supplementary Figure 2). Disbiome relationships were downloaded from the Disbiome API as a JSON file. The disease name was extracted from the *“disease_name”* field. The disease was normalized by a string match to MONDO, HPO, or GO terms, or by manual curation if no match was found automatically (Table 1). The organism name was extracted from the *“organism_name”* field and the organism ID from the *“organism_id”* field, which corresponded to an NCBI Taxonomy ID when present. To map microbes to NCBI Taxonomy terms when no organism ID was present, a direct string match between the microbe name in Disbiome and that in NCBI Taxonomy was first attempted. If no matches were found, brackets were included around the first term, assumed to be a genus name, and the match was attempted again. For example, after no NCBI Taxonomy match was found for “*Ruminococcus lactaris”*, “[*Ruminococcus] lactaris”* was successfully mapped to “*NCBITaxon:46228”*. Any remaining microbes were then manually mapped to a given NCBI Taxonomy. The predicate between a microbe and disease was determined from the *“qualitative_outcome”* field, where the predicate *“biolink:associated_with_increased_likelihood_of”* was used for the *“Elevated”* outcome and *“biolink:associated_with_decreased_likelihood_of”* was used for the *“Reduced”* outcome.

### 1.11. Wallen et al.

Data from *“Supplementary Table 1”* from Wallen et al.^21^ was downloaded for extraction of relationships between microbes and PD. Microbe names were extracted from the *“Species”* column. Similar to Disbiome, a direct string match between the microbe name in Wallen et al. and that in NCBI Taxonomy was first attempted, followed by the microbe name with brackets around the first string, followed by manual mapping. PD was manually assigned the MONDO term *“MONDO:0005180”*. The direction of the relationship between a given microbe and PD was determined based on the *“RA in PD”* and *“RA in NHC”* columns, which specify the relative abundance of the microbe in the disease and control groups, respectively, using Metagenomic Phylogenetic Analysis (MetaPhlAn). The *“biolink:associated_with_increased_likelihood_of”* predicate was used when the abundance in the disease group was greater than in the control group, while the *“biolink:associated_with_decreased_likelihood_of”* predicate was used when the abundance in the control group was greater than in the disease group. Only significantly differentially abundant microbes between the disease and control groups are included in the KG, based on values in the *“FDR”* column (representing false discovery rate) that are less than 0.05. These values indicate statistical significance, as determined using the Microbiome Multivariable Association with Linear Models (MaAsLin2) method^22^.

### 1.12. Comparative Toxicogenomics Database (CTD)

The latest release (January 2025) of chemical-disease associations was downloaded from the CTD^23^ data downloads page (*“CTD_chemicals_diseases.tsv.gz”*)C7/23/26 1:43:00 PMC^24^. The *“DiseaseID”* column contains Medical Subject Headings (MeSH) IDs that are mapped to MONDO terms using the *“xref”* fields of the MONDO OWL  file. Chemicals are extracted from the *“CasRN”* field or the *“ChemicalID”* field, which returns CAS-RN or MeSH IDs, respectively. Those IDs are mapped to ChEBI IDs using the Node Normalization API v1.4, which returns equivalent identifiers of chemicals from a variety of ontologies or standardized resources (Table 1). Chemicals are linked to diseases via the *“biolink:associated_with”* predicate.

### 1.13. Disease Taxon Expansion and Contingency Construction

To assess butyrate-producer enrichment among disease-associated taxa (Figure 6C), each microbe linked to IBD or PD through *“biolink:associated_with_increased_likelihood_of”* or *“biolink:associated_with_decreased_likelihood_of”* in KG-Microbe-Biomedical-Function was expanded to its pan-genome by recursively traversing *“biolink:subclass_of”* edges over NCBI Taxonomy. For each disease-associated taxon, all strain-level descendants were enumerated; when the strain set was empty, meaning no strain-rank children were present in NCBI Taxonomy, species-level descendants were used as a fallback. The expanded set was then deduplicated to a single record per NCBITaxon identifier across all parent taxa, producing the “A2” contingency. Any descendant reached from parents asserting conflicting directions of association was removed, corresponding to 9,580 descendants for IBD and 1,029 for PD. The resulting per-descendant table, with each row classified as a butyrate producer or non-producer based on the organismal and genomic trait representation in Figure 4A, is the deduplicated A2 contingency reported as the published Figure 6C statistic: IBD χ² = 497.26 and PD χ² = 918.80, with p = 3.7 × 10⁻¹¹⁰ and 8.0 × 10⁻²⁰², respectively. We confirmed robustness of the result’s direction and statistical significance under an alternative contingency that retains cross-direction conflict descendants in both bins and the association remains significant, with IBD χ² = 223.69 and PD χ² = 587.73, both p < 10⁻⁵⁰.

**Supplementary Table 1**

Comparison of microbiome-related knowledge graphs.

| **Resource** | **Total Taxa** | **Total Sources** | **Total Classes** |
| --- | --- | --- | --- |
| KG-Microbe | 1,012,214 | 17 | 39 |
| MetagenomicKG^25^ | 113,217 | 7 | 14 |
| MicrobiomeKG^26^ | >1000 | 1 (literature) | 28 |
| MiKG4MD^27^ | 45 | 1 (literature) | 7 |
| Pre-/Probiotics^28^ | Not specified | 1 (literature) | 10 |

**Supplementary Table 2**

Timing metrics for KG-Microbe construction steps.

| **Process** | **Time** | **Hardware** |
| --- | --- | --- |
| KG-Microbe download | 3m50.551s | MacBook M1 |
| KG-Microbe NCBI taxonomy trimming and transform | 2m58.057s | MacBook M1 |
| KG-Microbe transforms | 43m11.067s | MacBook M1 |
| KG-Microbe merge | 5m51.477s | MacBook M1 |
| Uniprot transform | 144m34.532s | NERSC compute node |
| KG-Microbe-Function and KG-Microbe-Biomedical-Function merges | 13m29.950s | NERSC login node |

**References**

1.	The UniProt Consortium *et al.* UniProt: the Universal Protein Knowledgebase in 2023. *Nucleic Acids Research* **51**, D523–D531 (2023).

2.	Hegde, H. & Joachimiak, M. P. uniprot2s3.

3.	Reimer, L. C. *et al.* Bac *Dive* in 2022: the knowledge base for standardized bacterial and archaeal data. *Nucleic Acids Research* **50**, D741–D746 (2022).

4.	Weinstein, M. P. & Lewis, J. S. The Clinical and Laboratory Standards Institute Subcommittee on Antimicrobial Susceptibility Testing: Background, Organization, Functions, and Processes. *J Clin Microbiol* **58**, e01864-19 (2020).

5.	Giske, C. G., Turnidge, J., Cantón, R. & Kahlmeter, G. Update from the European Committee on Antimicrobial Susceptibility Testing (EUCAST). *J Clin Microbiol* **60**, e00276-21 (2022).

6.	Madin, J. & Nielsen, D. Bacteria-Archaea-Traits.

7.	Buttigieg, P. *et al.* The environment ontology: contextualising biological and biomedical entities. *J Biomed Sem* **4**, 43 (2013).

8.	Hastings, J. *et al.* ChEBI in 2016: Improved services and an expanding collection of metabolites. *Nucleic Acids Res* **44**, D1214–D1219 (2016).

9.	Cooper, L. & Jaiswal, P. The Plant Ontology: A Tool for Plant Genomics. in *Plant Bioinformatics* (ed. Edwards, D.) vol. 1374 89–114 (Springer New York, New York, NY, 2016).

10.	Dooley, D. M. *et al.* FoodOn: a harmonized food ontology to increase global food traceability, quality control and data integration. *npj Sci Food* **2**, 23 (2018).

11.	PATO Ontology Consortium. PATO: Phenotypic Quality Ontology. https://github.com/pato-ontology/pato.

12.	Mungall, C. J., Torniai, C., Gkoutos, G. V., Lewis, S. E. & Haendel, M. A. Uberon, an integrative multi-species anatomy ontology. *Genome Biol* **13**, R5 (2012).

13.	Cébron, A. *et al.* BactoTraits – A functional trait database to evaluate how natural and man-induced changes influence the assembly of bacterial communities. *Ecological Indicators* **130**, 108047 (2021).

14.	Koblitz, J. *et al.* Media *Dive* : the expert-curated cultivation media database. *Nucleic Acids Research* **51**, D1531–D1538 (2023).

15.	Bansal, P. *et al.* Rhea, the reaction knowledgebase in 2022. *Nucleic Acids Research* **50**, D693–D700 (2022).

16.	Jackson, R. *et al.* OBO Foundry in 2021: operationalizing open data principles to evaluate ontologies. *Database* **2021**, baab069 (2021).

17.	Federhen, S. The NCBI Taxonomy database. *Nucleic Acids Research* **40**, D136–D143 (2012).

18.	Jackson, R. C. *et al.* ROBOT: A Tool for Automating Ontology Workflows. *BMC Bioinformatics* **20**, 407 (2019).

19.	INCATools. Ontology Access Kit (OAK).

20.	Janssens, Y. *et al.* Disbiome database: linking the microbiome to disease. *BMC Microbiol* **18**, 50 (2018).

21.	Wallen, Z. D. *et al.* Metagenomics of Parkinson’s disease implicates the gut microbiome in multiple disease mechanisms. *Nat Commun* **13**, 6958 (2022).

22.	Mallick, H. *et al.* Multivariable association discovery in population-scale meta-omics studies. *PLoS Comput Biol* **17**, e1009442 (2021).

23.	Davis, A. P. *et al.* Comparative Toxicogenomics Database’s 20th anniversary: update 2025. *Nucleic Acids Research* **53**, D1328–D1334 (2025).

24.	Comparative Toxicogenomics Database. CTD Data Downloads. https://ctdbase.org/downloads/.

25.	Ma, C., Liu, S. & Koslicki, D. MetagenomicKG: a knowledge graph for metagenomic applications. Preprint at https://doi.org/10.1101/2024.03.14.585056 (2024).

26.	Goetz, S. L., Glen, A. K. & Glusman, G. MicrobiomeKG: Bridging Microbiome Research and Host Health Through Knowledge Graphs. Preprint at https://doi.org/10.1101/2024.10.10.617697 (2024).

27.	Liu, T. *et al.* Predicting the relationships between gut microbiota and mental disorders with knowledge graphs. *Health Inf Sci Syst* **9**, 3 (2021).

28.	Liu, T., Lan, G., Feenstra, K. A., Huang, Z. & Heringa, J. Towards a knowledge graph for pre-/probiotics and microbiota–gut–brain axis diseases. *Sci Rep* **12**, 18977 (2022).
